# High-content CRISPR activation screens identify synthetically lethal RNA-based mechanisms to sensitize cancer cells to targeted T cell cytotoxicity

**DOI:** 10.1038/s41588-026-02561-7

**Published:** 2026-04-07

**Authors:** Reece Villarin Akana, Jeehyun Yoe, Olivia Laveroni, Chang Sun, Young-Min Kim, Livnat Jerby

**Affiliations:** 1 Department of Genetics, Stanford University, Stanford, CA USA; 2 Cancer Biology Program, Stanford University, Stanford, CA USA; 3 Stanford Cancer Institute, Stanford University School of Medicine, Stanford, CA USA; 4 Chan Zuckerberg Biohub, San Francisco, CA USA

**Keywords:** Immunosurveillance, Synthetic biology, Gene expression profiling, High-throughput screening

## Abstract

T cells recognize their target cells through the T cell receptor (TCR). Combining gain-of-function, single-cell and optical high-content screens, we identified RNA-based mechanisms that selectively sensitize target cells to TCR-specific T cell cytotoxicity. First, CRISPR activation screens in melanoma cells identify functionally diverse regulators of TCR-specific cytotoxicity, including *SAFB*, *KHDRBS1*, *MYC*, *CD44*, *WNT3A*, *WNT1* and others. Expressing sensitizing hits in cancer and virally infected cells restores TCR-specific cytotoxicity. Next, we developed in situ Perturb-seq for optical pooled genetic screens with in situ detection of perturbations and spatial transcriptomic readouts. Perturb-seq and in vivo–in situ Perturb-seq show that the hits converge on shared cell-autonomous and intercellular mechanisms, map gene–environment interactions and reveal that Wnt ligands activate T cells. Introducing a scalable approach to decode gene function at the cell and tissue level, the study uncovered context-specific gene functions to restore targeted T cell-based elimination of dysfunctional cells via synthetically lethal, RNA-based interventions.

## Abstract (web summary)

A series of in vitro and in vivo CRISPR activation screens combined with in situ Perturb-seq identify regulators of T cell-dependent cytotoxicity in melanoma.

## Main

Despite advances in immunotherapy and other cancer treatments, there are still ~600,000 cancer-related deaths a year in the USA alone^1^. Most immunotherapies leverage the remarkable ability of CD8 T cells to selectively eradicate cancer cells^2–5^, but cancer cells evolve to evade^4,6–9^. Cancer immune evasion has been comprehensively studied via CRISPR knockout screens, demonstrating that inhibition of antigen presentation^10–12^, interferon γ (IFNγ) responses^10–12^ and adhesion genes^13,14^ can drive evasion, while double-stranded RNA molecules^15^ and transposable elements^16^ can trigger certain immune responses. High-content genetic screens with single-cell transcriptomic^17^ and proteomic^13^ readouts have also helped uncover underlying mechanisms and investigate intercellular regulation by preserving spatial information^18,19^.

Studying immune evasion through the lens of gene knockout has helped identify immune evasion drivers to target via traditional drugs such as monoclonal antibodies and small molecule inhibitors^12,20^. Given the pivotal advances in RNA medicine^21,22^, gain-of-function screens in cancer cells can identify genes that selectively enhance targeted immune responses and uncover what we refer to as ‘immune RNA-based synthetic lethality’ for the development of RNA-based immunotherapies, as we propose and demonstrate here.

Combining CRISPR activation (CRISPRa) screens with Perturb-seq and pooled optical screens we identified RNA-based mechanisms that selectively enhance targeted T cell cytotoxicity, revealed a suite of immunomodulators, which are distinct from those identified via gene knockouts, and put forward high-throughput approaches to decode gene function at scale, opening avenues for precise enhancement of targeted T cell cytotoxicity.

## Results

### CRISPRa screens identified T cell cytotoxicity regulators

T cells recognize cancer (neo)antigens via the T cell receptor (TCR) and, on binding, release granzymes and perforins via the cancer–T cell-immune synapse to elicit cell death only in the target cells. TCR-specific T cell cytotoxicity provides one of the most precise mechanisms to eliminate cancer cells.

**Fig. 1:**
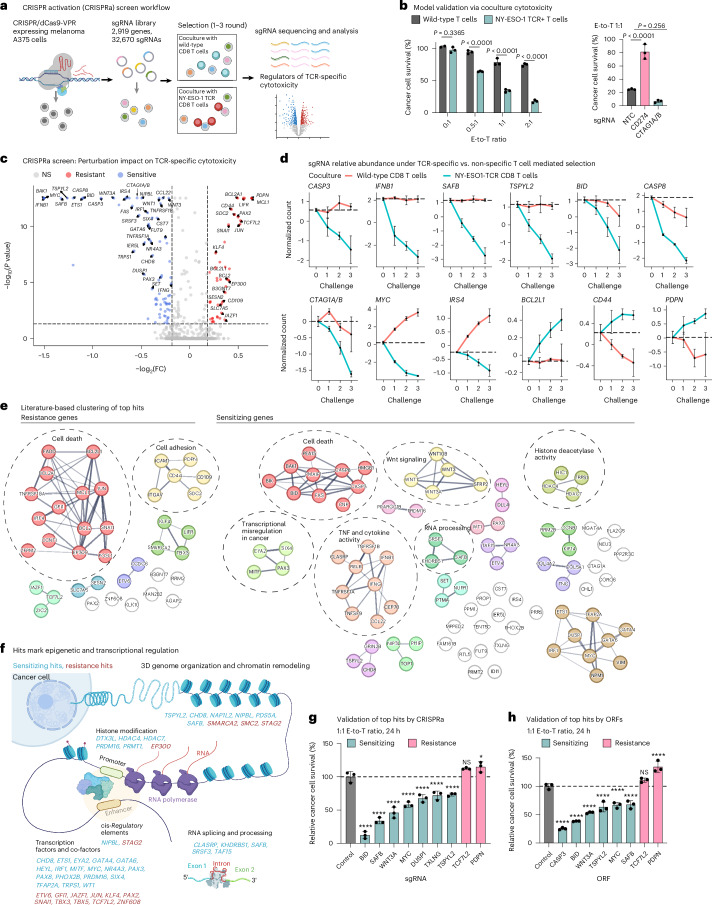
A CRISPRa screen in melanoma cells identified regulators of TCR-specific cytotoxicity. **a**, Overview of the CRISPRa screen. **b**, Model validation: TCR-specific cytotoxicity recorded in melanoma A375 cells cocultured for 24 h with wild-type or NY-ESO-1 TCR T cells (from the same donor) at different E-to-T ratios (left) and in A375 cells with CRISPRa of *CD274* (encoding for PD-L1), *CTAG1A/B* (encoding for the antigen NY-ESO-1) or NTC sgRNA at a 1:1 E-to-T ratio (right). Data are presented as mean ± s.d. with each data point representing a technical replicate (*n* = 3 per group). Groups were compared via two-way analysis of variance (ANOVA) and Šídák’s multiple-comparison test (left) and one-way ANOVA and Dunnett’s multiple-comparison test (right). **c**, Summary statistics of the CRISPRa screen results identifying perturbations that result in a selective (dis)advantage specifically in coculture with NY-ESO-1 TCR T cells versus wild-type T cells from the same donor, shown as an adjusted *P* value (BH FDR, MAGeCK; Methods and *y* axis) and fold-change (FC; *x* axis). **d**, Normalized counts of sgRNAs targeting the indicated genes in coculture with NY-ESO-1 TCR T cells or wild-type T cells (from the same donor). Normalized counts on the *y* axis are log_2_1p-transformed counts scaled to have a 0 value at challenge 0. Data are presented as mean ± s.d., *n* = 10 sgRNAs pre-target, except for *BCL2L1* with *n* = 20. The genes encoding for NY-ESO-1 (*CTAG1A* and *CTAG1B*) are targeted by the same ten sgRNAs and thus denoted as *CTAG1A/B*. **e**, CRISPRa top TCR-specific cytotoxicity hits (nodes) with edges denoting connections based on the STRING database^65^. **f**, Graphic depiction of the sensitizing (cyan) and resistance (red) hits involved in epigenetic and transcriptional regulation. **g**,**h**, Single hit validation with isogenic A375 cells. Relative survival of cancer cells in coculture with NY-ESO-1 TCR T cells versus wild-type T cells (from the same donor) is shown for A375 melanoma cells with CRISPRa-based (**g**) and ORF-based (**h**) overexpression of different hits. The values were normalized to the percentage survival observed in control cells and shown as mean ± s.d., with each data point representing a technical replicate (*n* = 3 per group). ^****^P < 0.0001, ^*^P < 0.05, NS, nonsignificant; one-way ANOVA, Dunnett’s multiple-comparison test. Illustrations in **a** and **f** created in BioRender; Jerby Lab https://biorender.com/qdelxmb (2026).

**Extended Data Fig. 1:**
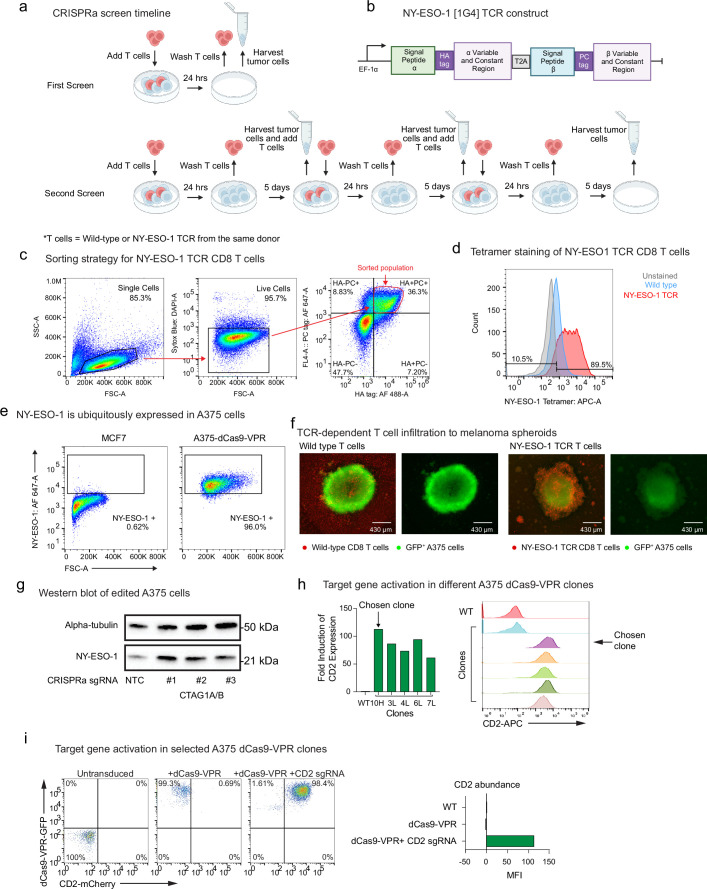
Combining TCR-engineering and CRISPRa to model and probe TCR-specific cytotoxicity. (**a**) Detailed schematics of the CRISPRa screen. (**b**) Schematic of the NY-ESO-1 [1G4] TCR construct used to engineer primary human CD8 T cells. (**c**) FACS-based isolation of live T cells transduced to express the NY-ESO-1 TCR by sorting for T cells double positive for HA (α chain) and PC (β chain) tags. (**d**) Staining of primary CD8 T cells transduced to express the NY-ESO-1 TCR with a tetramer of MHC-1 bound to the NY-ESO-1 peptide. (**e**) NY-ESO-1 intracellular protein levels, measured via flow cytometry in A375 cells. (**f**) GFP^+^ A375 spheroids in coculture with wild-type T cells (left) and NY-ESO-1 TCR T cells (right) isolated from the same donor. The experiment to derive microscopy images to depict this phenomenon was conducted once, depicting a representative pattern observed in this and other similar 3D cancer-T-cell coculture models. (**g**) Western blots confirming CRISPRa of *CTAG1A/B* (encoding for the NY-ESO-1 antigen) in A375 cells stably expressing CRISPR/dCas9-VPR using three different *CTAG1A/B* sgRNAs. This quantification was conducted once to complement flow cytometry-based validation of on-target CRISPRa-base gene activation and repeated quantification of target RNA levels at bulk and single cell level via qPCR and scRNA-seq, respectively. (**h**) Levels of CD2 activation in different A375 dCas9-VPR clones based on CD2 gene expression measured via qPCR (left) and protein levels on the cell surface measured via flow cytometry (right). (**i**) Target gene activation in the selected A375 clone, shown with flow cytometry gating (left) and Mean Fluorescence Intensity (MFI) in the sorted subsets (right). Illustrations in **a** and **b** created in BioRender; Jerby Lab https://biorender.com/qdelxmb (2026). Source data

To uncover genes that (de)sensitize cancer cells to TCR-specific cytotoxicity, we conducted a CRISPRa screen in melanoma cells under selection with TCR-specific and TCR-nonspecific T cells (Fig. 1a and Extended Data Fig. 1a). In brief, we isolated human primary CD8 T cells from blood samples and transduced them to express a TCR with clinical activity^23–25^ that recognizes the NY-ESO-1 antigen, constitutively expressed by A375 melanoma cells (Fig. 1b and Extended Data Fig. 1b–f). We transduced A375 cells to stably express CRISPR–dCas9-VPR^26,27^ (Extended Data Fig. 1g–i) and a library of 32,670 single-guide RNAs (sgRNAs), targeting a total of 2,919 genes (10 sgRNAs per gene) and 560 nontargeting control (NTC) gRNAs (Supplementary Table 1), enriched with genes associated with immunotherapy response in patients with melanoma^28^. We challenged the melanoma cells with up to three rounds of coculture (0.25:1 effector-to-target (E-to-T) ratio for 24 h) with either the NY-ESO-1 TCR or wild-type T cells from the same donor (Fig. 1a and Extended Data Fig. 1a).

**Extended Data Fig. 2:**
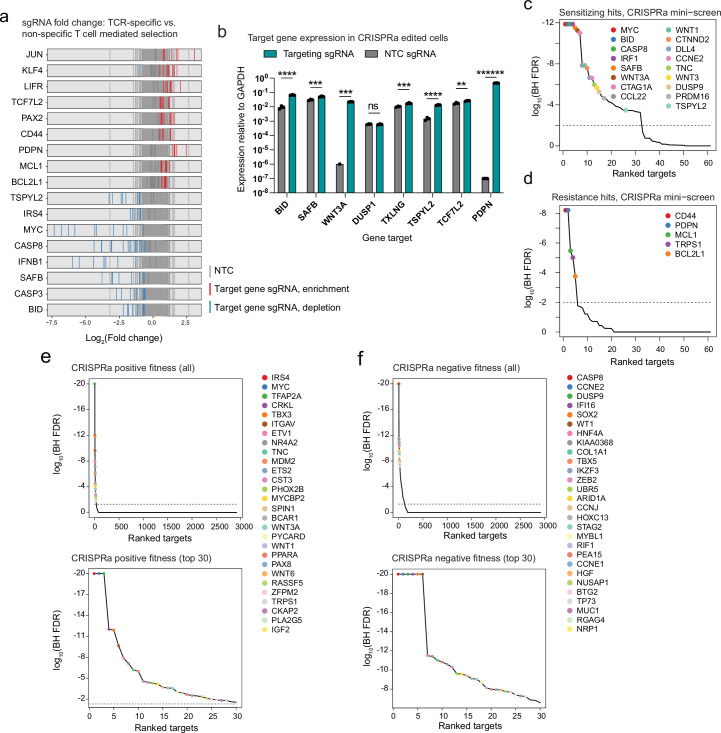
Detection and validation of TCR-specific cytotoxicity regulators identified via CRISPRa screens. (**a**) Log fold change in relative abundance of sgRNAs in coculture with NY-ESO-1 TCR vs. wild-type T cells, shown for top resistance and sensitizing hits. Grey lines mark NTC sgRNAs. (**b**) Expression of top hits in A375 cells transduced to express top hits via CRISPRa sgRNAs compared to control (NTC) A375 cells; expression is quantified via qPCR and normalized to *GAPDH* expression. Data are presented as mean values (*n* = 3 technical replicates per group). ^****^q < 0.0001, ^***^q < 0.001, ^**^q < 0.01, unpaired one-tailed t-test, Benjamini, Krieger, and Yekutieli (BKY) FDR. (**c**, **d**) CRISPRa follow-up mini-screen results, showing TCR cytotoxicity sensitizing (c) and resistance (d) hits based on BH FDR (Fisher combined MAGeCK test, Methods; y axis). Hits (BH FDR < 0.05) are marked and listed. (**e**, **f**) Baseline fitness CRISPRa hits: Positive (e) and negative (f) hits ranked (x axis) based on BH FDR (Fisher combined MAGeCK test, Methods; y axis), depicting all 2,919 genes (top) or only the top 30 hits (bottom).

A total of 38 and 90 genes showed significant enrichment and depletion specifically in the context of TCR-specific cytotoxicity and were denoted as resistance and sensitizing hits, respectively (Benjamini–Hochberg (BH) false discovery rate (FDR) <0.05, MAGeCK^29,30^; Fig. 1c–f, Extended Data Fig. 2a, Supplementary Table 2 and Methods). Resistance and sensitizing sgRNAs were monotonically enriched and depleted with every challenge, respectively (Fig. 1d) and *CTAG1A*/*B* (encoding for NY-ESO-1) were sensitizing hits (BH FDR < 1 × 10^−17^, MAGeCK; Fig. 1c,d). Validating the results of the screen, isogenic A375 lines, each transduced with a single sgRNA or open reading frame (ORF) to overexpress one of a selected set of top hits (Extended Data Fig. 2b), confirmed the expected increase or decrease in TCR-specific cytotoxicity (Fig. 1g,h) and a follow-up mini-screen focused on top hits recapitulated the top hits identified in the large screen (Extended Data Fig. 2c,d).

Comparing the hits identified here to those identified in a collection of eight cancer immune evasion CRISPR knockout screens^10–12,16,31–34^, nine of the CRISPRa-resistance hits (*P* = 2.31 × 10^−4^, hypergeometric test: *BCL2*, *BCL2L1*, *CD44*, *EP300*, *FADD*, *ITGAV*, *JUN*, *MAN2B2* and *MCL1*) and 13 of the sensitizing hits (*P* = 1.64 × 10^−4^, hypergeometric test: *CASP3*, *CASP8*, *CHD8*, *CLASRP*, *ETS1*, *FAS*, *IRF1*, *KHDRBS1*, *NIPBL*, *PPP2R3C*, *SAFB*, *TNFRSF1A* and *TNFRSF1B*) were previously reported to sensitize or confer resistance on knockout, respectively, indicating the distinction between knockout and activation-based assessment of gene function. Comparing the hits identified here to those identified in an in vitro CRISPRa screen previously conducted in A375 cells to identify genes that confer resistance to T cell cytotoxicity^31^, only five of the resistance hits overlap (*P* = 6.27 × 10^−3^, hypergeometric test, five overlapping hits: *BCL2A1*, *COL4A3*, *LIFR*, *MCL1* and *SDC2*), indicating the distinction between general and TCR-specific T cell cytotoxicity.

The hits identified here are functionally diverse (Fig. 1e,f), spanning the full spectrum of transcriptional and epigenetic regulation (BH FDR **<** 7.54 × 10^−5^, hypergeometric test), including regulators of three-dimensional (3D) DNA architecture, chromatin and histone modifiers, transcription factors and RNA processing or splicing genes (for example, resistance: *EP300*, *ETV6*, *JAZF1* and *TCF7L2*; and sensitizing: *ETS1*, *EYA2*, *GATA4*, *GATA6*, *KHDRBS1*, *NIPBL*, *NR4A3*, *PRDM16*, *SAFB*, *SRSF3* and *TSPYL2*; Fig. 1f). Many of the hits are involved in developmental processes (for example, resistance: *GFI1*, *PAX2*, *SNAI1*, *TCF7L2* and *ZIC2*; and sensitizing: *MITF*, *PAX8*, *PHOX2B* and *TNC*) and insulin sensing (for example, resistance: *TCF7L2* and *JAZF1*; and sensitizing: *IRS4* and *PEA15*) and include a suite of receptors, cell surface and secreted proteins (for example, resistance surface glycoproteins: *CD44* and *PDPN*; and sensitizing: *WNT1*, *WNT3*, *WNT3A* and *TNC*).

As expected, the resistance hits are enriched for anti-apoptotic genes (BH FDR = 2.58 × 10^−5^, hypergeometric test: *BCL2A1*, *MCL1*, *BCL2L1* and *BCL2*), whereas the sensitizing hits are enriched for pro-apoptotic genes (BH FDR = 2.37 × 10^−4^, hypergeometric test: for example, *BAK1*, *BID*, *CASP3*, *CASP8* and *FAS*). The expression of resistance and sensitizing hits in The Cancer Genome Atlas melanoma cohort (>400 patients)^35^ is associated with worse and better overall survival, respectively (BH FDR < 0.05, Cox’s regression; Supplementary Fig. 1a and Supplementary Table 2a).

Based on baseline fitness effects (irrespective of T cell cytotoxicity), the screen resulted in 115 and 33 positive and negative fitness hits (Extended Data Fig. 2e,f and Supplementary Table 2a,c), which are enriched for oncogenes and tumor suppressors, respectively (*P* = 5.61 × 10^−6^, 0.017, hypergeometric test, respectively) and distinct from the TCR cytotoxicity hits (*r* = −0.056, −0.037, *P* = 0.002, 0.048, Pearson’s correlation). Of the 90 sensitizing hits, 68 did not show a significant connection to fitness at baseline, indicating that these perturbations are synthetically lethal with TCR-specific cytotoxicity. These TCR-specific sensitizing hits include central cell death regulators, such as *CASP3*, *BID* and *BAK*.

**Fig. 2:**
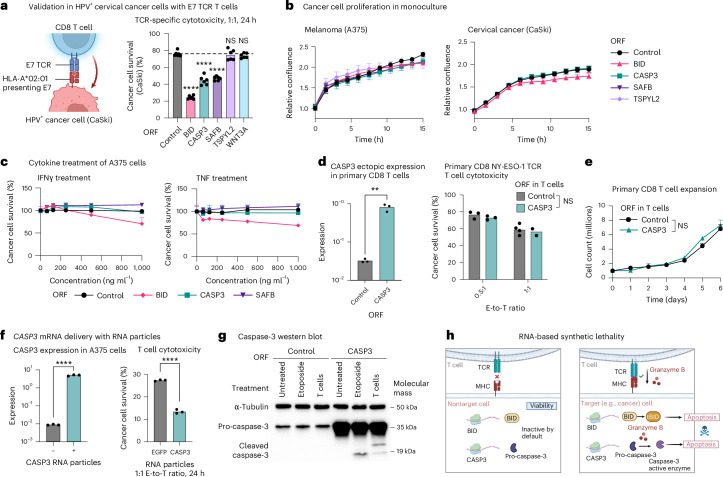
Immune RNA-based synthetic lethality as a mechanism to selectively eliminate target cells, demonstrated with *CASP3* overexpression. **a**, Left: illustration of the coculture used to test top hits in a different cancer type and with a different TCR and antigen. HPV16^+^ CaSki cells express the HPV16 oncoprotein E7 and present it on major histocompatibility complex (MHC)-I (HLA-A2 allele), allowing E7 TCR-specific cytotoxicity. Validations of this model are depicted in Extended Data Fig. 3. Right: percentage of surviving CaSki cells (*y* axis) in coculture with E7 TCR T cells, shown for CaSki cells transduced to express different ORFs (mean ± s.d., *n* = 6 technical replicates per group). The *P* value is from two-way ANOVA and Dunnett’s multiple-comparison test. **b**, Confluency of different A375 (left) and CaSki ORF (right) lines (that is, transduced with different ORFs for constitutive expression of different hits) measured via Incucyte. Cells were monitored for 16 h, with images taken every 1.5 h (mean ± s.d., *n* = 4–6 technical replicates per group). **c**, Viability of A375 ORF lines measured via PrestoBlue after 24-h treatment with varying doses (*x* axis) of IFNγ (left) or TNF (right) (mean ± s.d., *n* = 2–3 technical replicates per group). **d**,**e**, Primary CD8 T cells transduced with lentivirus for ORF-based overexpression of *CASP3*. *CASP3* expression was measured via quantitative PCR (qPCR) and normalized to *GAPDH* (**d**, left); cytotoxicity was measured in 24-h coculture with A375 cells at 0.5:1 and 1:1 E-to-T ratio (**d**, right). Data are presented as mean values (*n* = 3 technical replicates per group). ^**^P < 0.01, two-tailed Student’s *t*-test. **e**, T cell counts over 6 d in monoculture (mean ± s.d., *n* = 2–3, technical replicates per group). NS, two-way ANOVA. **f**, Delivery of *CASP3* RNA via cationic lipid-based or polymer-based nanoparticle in A375 cancer cell coculture with NY-ESO-1 T cells. *CASP3* expression in the A375 cells was measured via qPCR and normalized to *GAPDH* (left) and cancer cell survival with RNA delivery in coculture versus monoculture measured via PrestoBlue (right). Data are presented as mean values (*n* = 3 technical replicates per group). ^****^P < 0.0001, unpaired, two-tailed Student’s *t*-test. **g**, Western blot of pro-caspase-3 and cleaved caspase-3 protein levels measured in control and CASP3^OE^ A375 cells that were untreated in monoculture, treated with etoposide (24 h at 2.5 μM) in monoculture or untreated in cocultured with NY-ESO-1 TCR CD8 T cells (1:1 E-to-T ratio, 24 h; Methods). The experiment was conducted once to demonstrate this protein-level property of caspase-3 in accordance with the screen data and other experiments conducted here. **h**, Proposed model of *CASP3* RNA-based synthetic lethality with TCR-specific cytotoxicity. Illustrations in **a** and **h** created in BioRender; Jerby Lab https://biorender.com/qdelxmb (2026). Source data

**Extended Data Fig. 3:**
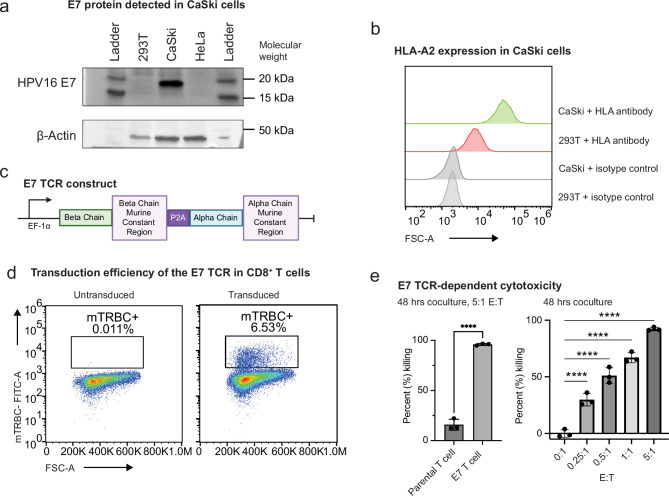
Generating and validating E7 TCR T cells. (**a**) Western blot of the HPV16 E7 protein in CaSki cells. 293 T and HeLa cells serve as negative controls. This readout was obtained once to complement E7 presentation by CaSki cells, confirmed repeatedly via E7-TCR-dependent cytotoxicity. (**b**) Flow cytometry analysis of HLA-A2 in CaSki cells. 293 T cells serve as positive controls. (**c**) E7 TCR construct. (**d**) Representative FACS plots of T cells transduced to express the E7 TCR construct and stained with an mTRBC antibody to isolate E7 TCR T cells. (**e**) Percentage of CaSki cancer cells killed (mean ± SD, *n* = 3 technical replicated per group, y axis) after 48 h in coculture with parental or E7 TCR T cells at 5:1 E:T (left) or with E7 TCR T cells at varying E:T ratios (x axis; right). Left: ^****^P < 0.001, unpaired, two-tailed t-test. Right: ^****^P < 0.001, ordinary one-way ANOVA, Dunnett’s multiple comparisons test. Illustration in **c** created in BioRender; Jerby Lab https://biorender.com/qdelxmb (2026). Source data

Further investigating *CASP3*, we demonstrated the concept of ‘immune RNA-based synthetic lethality’, where the expression of a gene is not lethal at baseline, but enhances TCR-specific cytotoxicity. First, melanoma (A375) and cervical cancer (CaSki) cells with ORF-based overexpression of *CASP3* (CASP3^OE^) were significantly more sensitive to TCR-specific cytotoxicity (Figs. 1h and 2a and Extended Data Fig. 3) but showed no significant change in their viability and proliferation (Fig. 2b). Second, CASP3^OE^ A375 cells were not more susceptible to tumor necrosis factor (TNF), IFNγ and a Bcl-2 inhibitor compared to control A375 cells (Fig. 2c and Supplementary Fig. 1b–d). Third, ORF-based overexpression of *CASP3* in primary CD8 T cells did not impact T cell viability, expansion and cytotoxicity (Fig. 2d,e). Fourth, *CASP3* RNA delivery to a coculture of wild-type A375 and NY-ESO-1 TCR T cells via a cationic lipid-based or polymer-based nanoparticle significantly enhanced TCR-specific cytotoxicity (Fig. 2f). Mechanistically explaining this immune RNA-based synthetic lethality, CASP3^OE^ A375 cells maintain the protein in its inactive pro-caspase-3 form in monoculture, which is cleaved to its active form in coculture with targeting T cells (Fig. 2g,h). In contrast, cleaved caspase-3 is undetectable in the control A375 cells, both in monoculture and in coculture (Fig. 2g), demonstrating how restoration of *CASP3* expression restores cancer cell responsiveness to T cell cytotoxicity.

### Perturb-seq reveals converging effects across hits

**Fig. 3:**
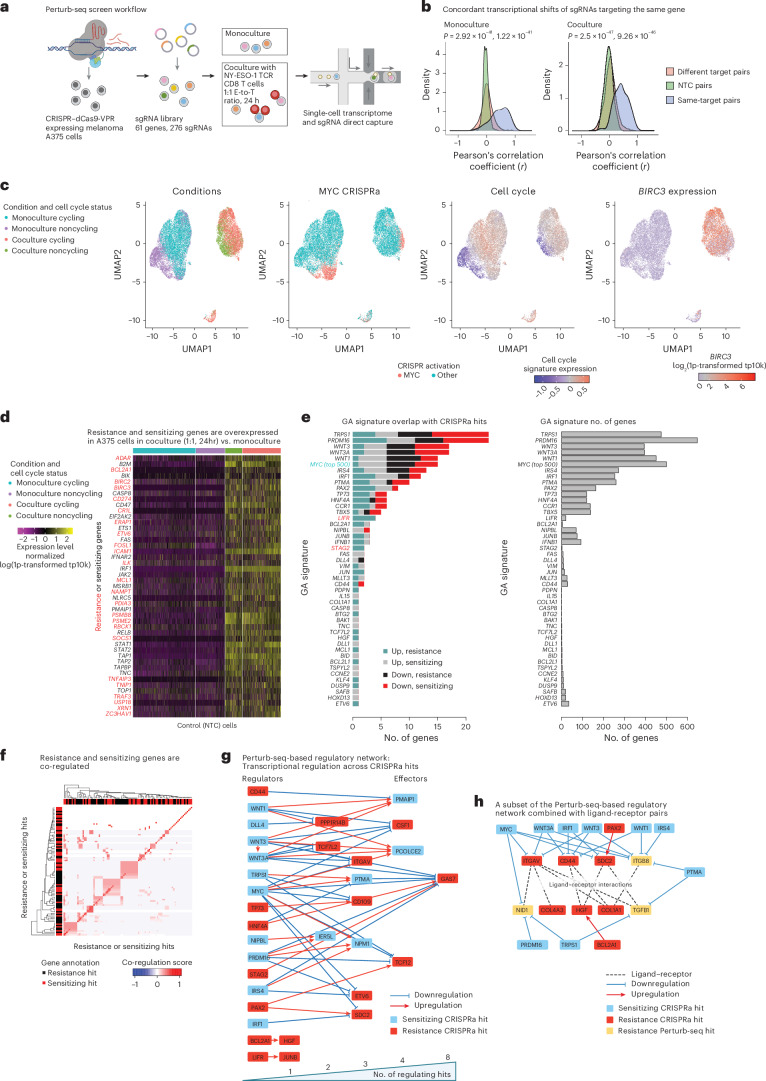
Perturb-seq screen reveals convergence of hits. **a**, Overview of Perturb-seq screen. **b**, Concordant transcriptional shifts of sgRNAs targeting the same gene. Pearson’s correlation coefficients of the transcriptional shifts (*z*-scores) of pairs of sgRNAs stratified to sgRNAs targeting the same or different genes and to pairs of NTC sgRNAs (Methods). The coefficient distributions are shown when computed based on the monoculture (left) and coculture (right) Perturb-seq data. **c**, Uniform Manifold Approximation and Projection (UMAP) of Perturb-seq single-cell gene expression profiles, with cells colored based on (from left to right) culture conditions, *MYC* sgRNA detection, expression of a cell-cycle signature^66^ and expression of the anti-apoptotic gene *BIRC3* (log_2_1p-transformed transcript per 100,00 (tp100K); Methods). **d**, Normalized expression values (centered and scaled log1p-transformed tp10k) shown across the control (NTC) cells (columns) in monoculture or 24-h coculture with NY-ESO-1 TCR T cells at a 1:1 E-to-T ratio. They were further stratified based on cell-cycle status (top horizontal bar). The genes depicted are immune-resistance (red) and immune-sensitizing (black) hits identified based on this and prior CRISPR screens^10–12,16,31–34^, which are also overexpressed in the control cells in coculture compared to monoculture. **e**, Left: the number of CRISPRa-sensitizing and CRISPRa-resistance hits included in each of the 61 GA signatures, shown for both upregulated and downregulated subsets of each signature. Right: the total number of genes (*x* axis) included in each GA signature (*y* axis). **f**, Clustering of sensitizing and resistance genes based on their co-regulation in the Perturb-seq screen (Supplementary Note 1). **g**, Regulatory Perturb-seq-based network. Nodes represent resistance (red) and sensitizing (light-blue) hits; edges denote that the source node is upregulating (red) or downregulating (blue) the transcription of the target node based on the Perturb-seq data. Hits that regulate other hits are to the left and those that are regulated by other hits are to the right, such that the more hits that regulate the gene (that is, a higher number of incoming edges), the further it is to the right (bottom bar). Importantly, only the CRISPRa hits that were perturbed in the Perturb-seq screens can have outgoing edges. This network thus captures only a subset of the regulatory interactions between CRISPRa hits. **h**, Combining the regulatory Perturb-seq-based network (annotations as in **g**) with ligand–receptor interactions (dashed lines) demonstrating that multiple hits either regulate or interact with a ligand–receptor hub. Nodes include CRISPRa-resistance (red) and CRISPRa-sensitizing (light-blue) hits, as well as resistance Perturb-seq hits (yellow), which are annotated as such because they are downregulated by two or more CRISPRa-sensitizing hits. Illustration in **a** created in BioRender; Jerby Lab https://biorender.com/qdelxmb (2026).

As hits were enriched for transcriptional and epigenetic regulators (BH FDR < 7.54 × 10^−5^, hypergeometric test; Fig. 1f), we turned to investigate underlying mechanisms via a CRISPRa Perturb-seq screen^17^ (276 sgRNAs targeting 61 genes or hits; Supplementary Table 3). CRISPRa A375 cells, cultured in monoculture or with the NY-ESO-1 TCR T cells (24-h coculture at 1:1 E-to-T ratio), were profiled via 5ʹ-droplet-based single-cell RNA sequencing (scRNA-seq) together with direct capture and sequencing of the sgRNA spacer^36^ (Fig. 3a), resulting in a total of 20,207 high-quality scRNA profiles from monoculture (12,640 cells) and coculture (7,567 cells), with 2,660 control cells (that is, carrying NTC sgRNAs; Fig. 3b,c).

T cells substantially impacted the cancer cell transcriptome (Fig. 3c), both priming and protecting the cancer cells from the immune attack (Fig. 3d and Supplementary Table 4). Genes overexpressed in coculture compared to monoculture (denoted as co-UP genes, log(fold-change) >0.25, BH FDR < 0.01, edgeR^37,38^; Methods) include pathways required for TCR-based target cell recognition—antigen presentation, cell adhesion (for example, *ICAM1*), cytokines (for example, *CXCL1*, *CXCL11*, *CXCL9* and/or *CXCL10*), JAK-STAT pathway and IFN response genes (for example, *IRF1*, *IRF2*, *IRF7*, *IRF9, ISG15*, *ISG20*, *JAK2*, *STAT1*, *STAT2*, *STAT3*)—potentially explaining why further activation of these pathways via CRISPRa did not sensitize the cancer cells to TCR-specific cytotoxicity. Co-UP genes are enriched for both immune-sensitizing (*P* = 8.88 × 10^−16^, hypergeometric test) and immune-resistance genes (*P* < 1 × 10^−17^, hypergeometric test; Fig. 3d) identified here and in previous CRISPR screens^10–12,16,31–33^. Co-UP genes are enriched for positive regulators of apoptosis (*P* < 1 × 10^−17^, hypergeometric test) but also include central anti-apoptotic genes (for example, *BCL2A1*, *MCL1*, *BCL3*, *BIRC2* and *BIRC3*; *P* < 1 × 10^−17^, hypergeometric test; Fig. 3c,d). Genes underexpressed in coculture versus monoculture (denoted as co-DOWN genes, log(fold-change) < −0.25, BH FDR < 0.01, edgeR) are associated with tissue development and cell differentiation (*P* < 1 × 10^−17^, hypergeometric test; Supplementary Table 4).

**Extended Data Fig. 4:**
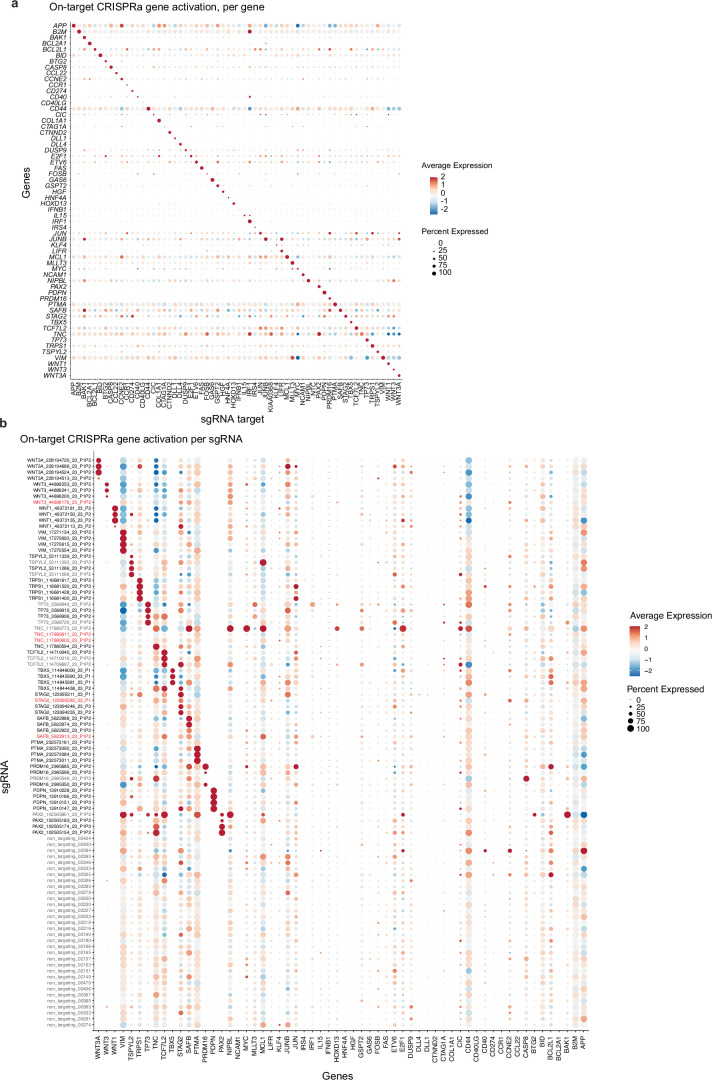
Perturb-seq screen confirms CRISPRa on-target gene activation (part 1). (**a**) Expression level (color bar, shown as centered and scaled log1p-transformed average tp10k values) and percentage of cells expressing each of the target genes (rows) in cells stratified based on the sgRNA target (columns). (**b**) Expression levels as in (a) and percentage of cells expressing each of the target genes (columns) in cells stratified based on the individual sgRNA (rows), shown for a subset of the sgRNAs. In red are sgRNAs that do not show a statistically significant overexpression of the target gene (BH FDR > 0.05, edgeR test).

**Extended Data Fig. 5:**
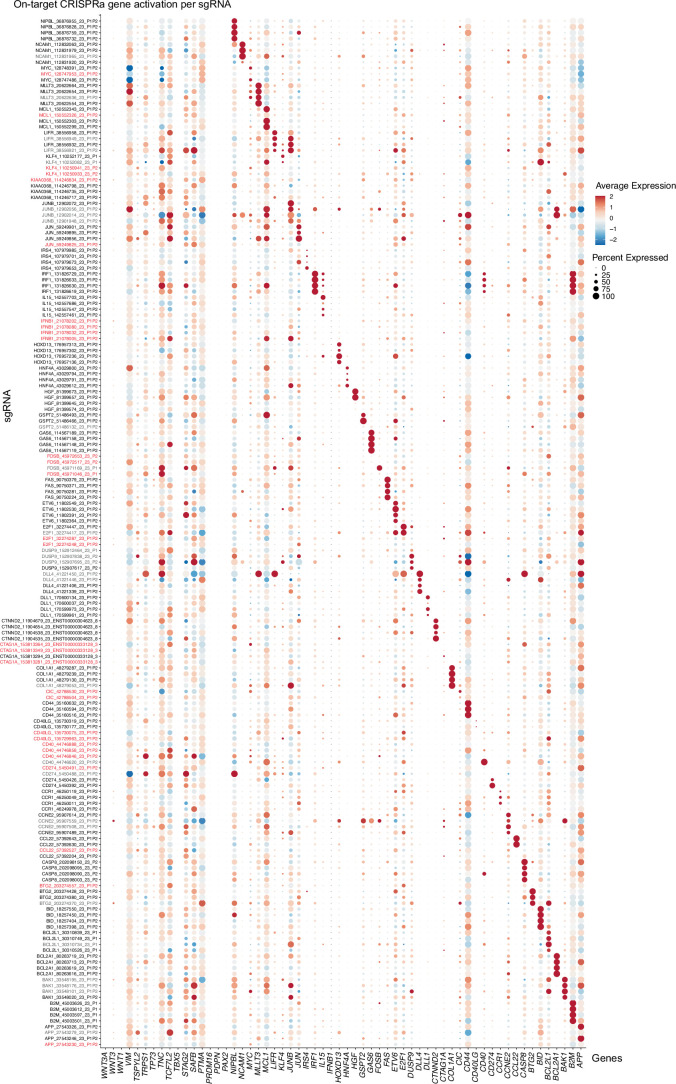
Perturb-seq screen confirms CRISPRa on-target gene activation (part 2). Expression level (color bar, depicting average expression based on centered and scaled log1p-transformed tp10k values) and percentage of cells expressing each of the target genes (columns) in cells stratified based on the individual sgRNA (rows), shown for the rest of the sgRNAs not shown in Extended Data Fig. 4b. In red are sgRNAs that do not show a statistically significant overexpression of the target gene (BH FDR > 0.05, edgeR test).

**Extended Data Fig. 6:**
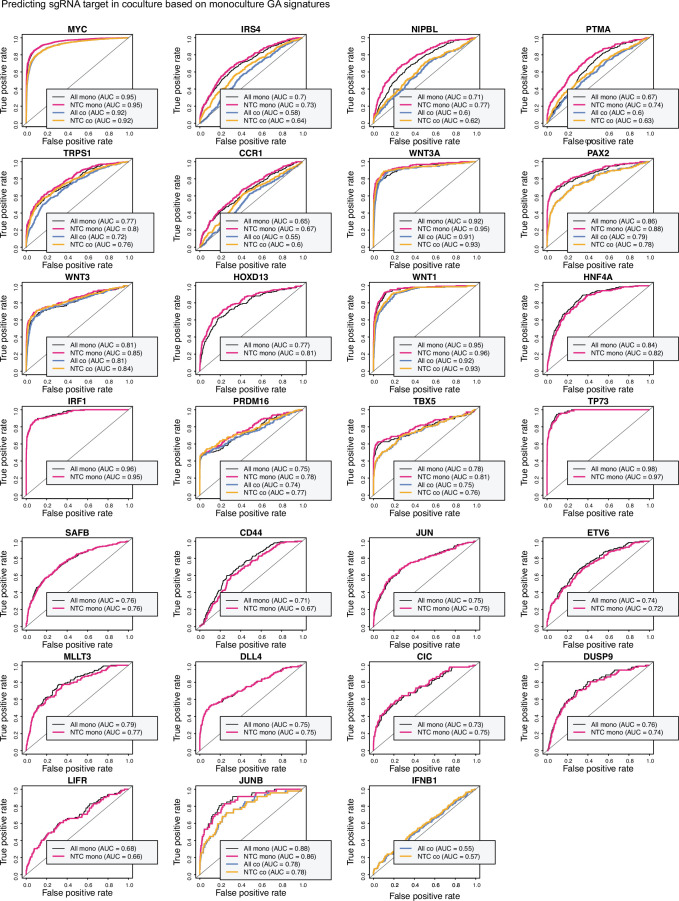
Perturb-seq coculture GA signature validation in monoculture. GA signatures derived separately from monoculture (black and pink) or coculture (blue and yellow) were applied to classify cells as subject to the pertaining gene activation, when considering all cells in the Perturb-seq monoculture dataset, or only control (NTC) and cells with the pertaining gene activation based on sgRNA detection in monoculture. Only GA signatures with at least 5 genes were used as predictors. Mono (monoculture), co (coculture).

**Extended Data Fig. 7:**
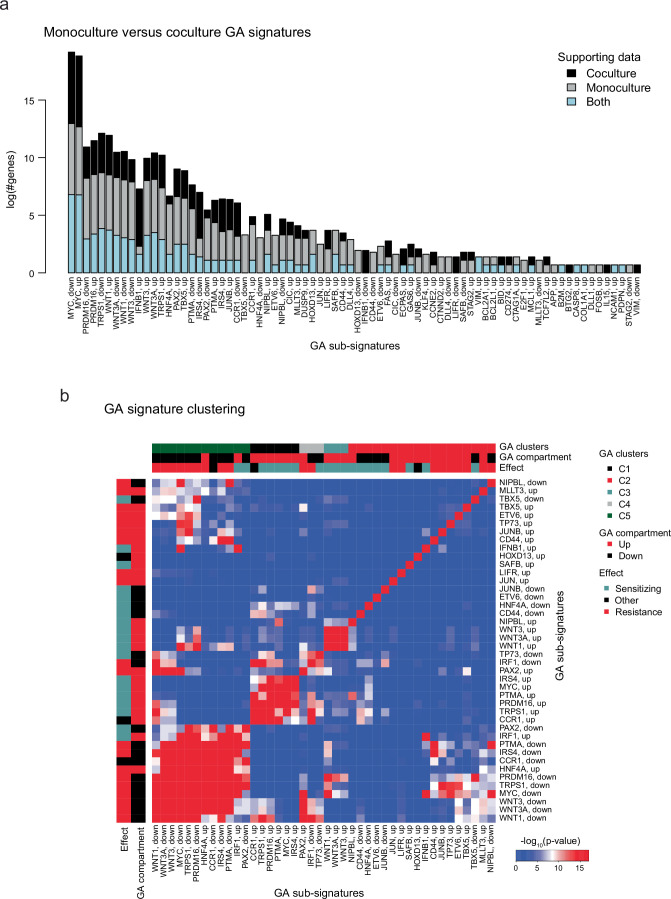
GA signature overlaps. (**a**) GA signatures stratified to their up- and down-regulated compartments (x axis, denoted as GA sub-signatures) and the log-transformed number of genes (y axis) that are differentially expressed only in monoculture (grey), only in coculture (black), or in both (cyan) conditions. (**b**) Overlap between pairs of GA sub-signatures (quantified as log_10_-transformed one-sided hypergeometric p-value, without correction for multiple hypothesis testing, bottom right color bar) shown as a clustered heatmap. The sub-signatures assignment to clusters is noted (C1-C5). The up- and down-regulated sub-signatures of sensitizing and resistance hits, respectively, are annotated as having a “sensitizing” effect. The up- and down-regulated sub-signatures of resistance and sensitizing hits, respectively, are annotated as having a “resistance” effect.

**Extended Data Fig. 8:**
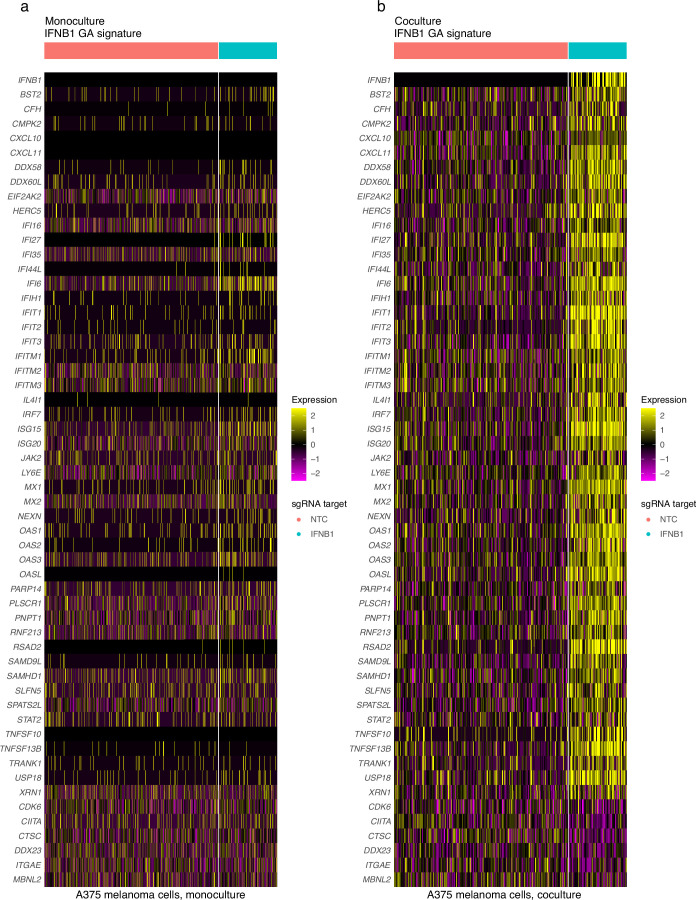
*IFNB1* GA signature identified in coculture Perturb-seq data. (**a**, **b**) Normalized expression values (centered and scaled log1p-transformed tp10k) of IFNB1 GA signature genes (identified in coculture Perturb-seq data; rows) across the NTC and IFNB1 CRISPRa cells (columns) in (**a**) monoculture and (**b**) coculture conditions.

**Extended Data Fig. 9:**
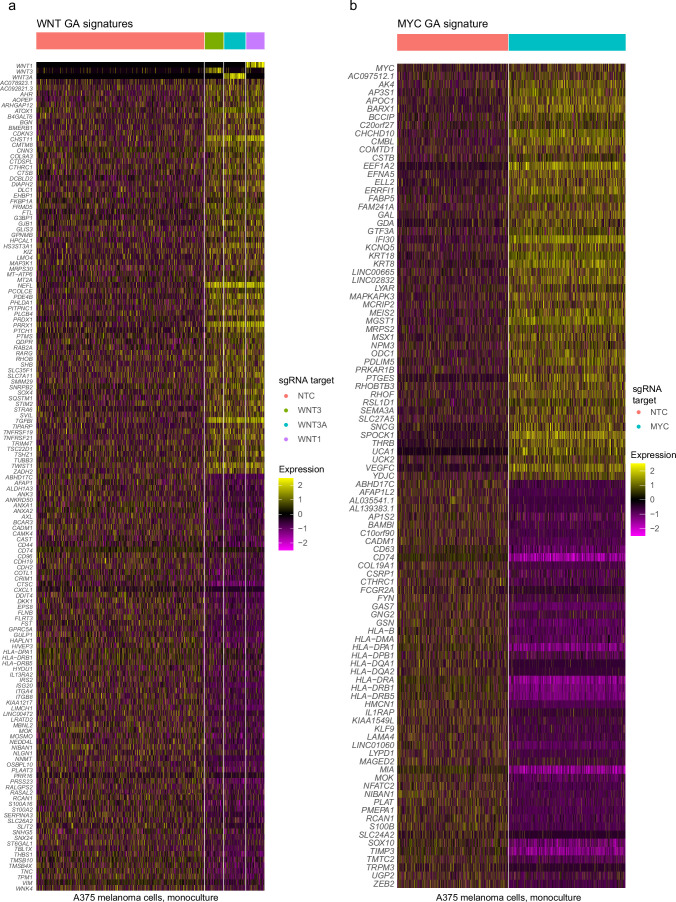
*WNT* and *MYC* GA signatures. Normalized expression values (centered and scaled log1p-transformed tp10k) of (**a**) genes included in all three WNT GA signatures, as well as the genes *WNT1*, *WNT3*, and *WNT3A* (rows), across the control and WNT1, WNT3, and WNT3A CRISPRa cells (columns and topmost color bar) in monoculture, and (**b**) MYC GA signature genes (rows) across the NTC and MYC CRISPRa cells (columns and topmost color bar) in monoculture.

Next, we identified a gene activation (GA) signature per perturbation, consisting of all the genes significantly overexpressed or underexpressed in the perturbed cells (expressing one of the sgRNAs targeting the pertaining gene) compared to the control cells (BH FDR < 0.01, edgeR test; Extended Data Figs. 4–9). Four lines of evidence testify to the validity of the GA signatures. First, for 84% of the sgRNAs, the cells where the sgRNA was detected significantly overexpress the target gene compared to the control cells (*P* < 0.05, edgeR; Extended Data Figs. 4 and 5 and Supplementary Table 5). Second, sgRNAs targeting the same gene had similar transcriptional effects (Fig. 3b and Supplementary Table 6). More specifically, the transcriptional shifts measured by a normalized *z*-score per sgRNA (Methods) were most correlated when considering sgRNAs targeting the same gene (Fig. 3b) and the GA signature identified for a given gene based on two out of its three sgRNAs was significantly overexpressed (*P* < 0.05, one-sided Student’s *t*-test) in the cells carrying the left-out sgRNA compared to the control cells in 81% of the cases (average area under the receiver operating characteristic curve (AUROC) = 0.75; Supplementary Figs. 2 and 3, Supplementary Table 6 and Methods). Third, the GA signatures defined based on monoculture data generalized to the coculture data, and vice versa (*P* < 0.05, one-sided Student’s *t*-test, in 100% of the cases and AUROC > 0.7 in 70% of the cases; Extended Data Fig. 6 and Supplementary Fig. 4; here the target genes were removed from the GA signatures to avoid favorably skewing the results). Of note, *IFNB1* was an exception, because CRISPRa *IFNB1* led to *IFNB1* overexpression and transcriptional alterations only in coculture (Extended Data Figs. 6, 7a and 8 and Supplementary Fig. 4). Fourth, the GA signatures generalized to a published CRISPRa screen conducted in K562 cells (Supplementary Fig. 5a)^39^.

GA signature size varied and was proportional to other measures of perturbational transcriptional shifts^40^ (Fig. 3e, Supplementary Figs. 5b–e and 6–9 and Supplementary Table 7a). Overlap between GA signatures defined clusters of similar signatures (Extended Data Fig. 7b and Supplementary Table 7b). As expected, *WNT1*, *WNT3* and *WNT3A* GA signatures showed the most significant overlap (Jaccard index >0.1, *P* < 1 × 10^−17^, hypergeometric test; Extended Data Fig. 9a). However, similar to the transcriptional response to T cells, many perturbations upregulated both immune-sensitizing and immune-resistance genes and downregulated both immune-sensitizing and immune-resistance genes (Fig. 3e and Supplementary Fig. 5e). Defining a co-regulation score for each pair of genes based on the number of perturbations where the two genes are both upregulated or both downregulated further shows that co-regulation modules include a mix of sensitizing and resistance genes (Fig. 3f). The Perturb-seq data thus reveal a regulatory network, but additional information is needed to conclude which downstream effects can explain the resistance or sensitizing effects of the perturbations.

We thus introduced the concept of ‘Perturb-seq hits’—genes that are regulated by multiple hits in a manner that supports their involvement exclusively in either positive or negative regulation of the phenotype of interest, in this case, TCR-specific cytotoxicity (Methods). Genes upregulated and downregulated by multiple resistance and sensitizing CRISPRa hits, respectively, were nominated as candidate resistance Perturb-seq hits. Likewise, genes upregulated and downregulated by multiple sensitizing and resistance CRISPRa hits, respectively, were nominated as candidate-sensitizing Perturb-seq hits. Genes that were nominated as only resistance or only sensitizing candidate Perturb-seq hits were defined as the final set of Perturb-seq hits.

Pruning the Perturb-seq-based regulatory network to include only CRISPRa hits that are also Perturb-seq hits reveals that CRISPRa hits regulate other CRISPRa hits in a manner that can explain their phenotype (Fig. 3g). At the center of this network (Methods) is the neuronal development gene *GAS7*, a resistance CRISPRa hit that is upregulated by two other resistance CRISPRa hits (*HNF4A* and *STAG2*) and downregulated by six sensitizing CRISPRa hits (*MYC*, *IRS4*, *PTMA*, *WNT3*, *WNT3A* and *PRDM16*). In accordance with this, both resistance and sensitizing CRISPRa hits are enriched for neuronal development genes (for example, *OLIG2*, *NEURL1*, *NR4A2* and *TNC*; *P* < 0.005, hypergeometric test), potentially related to melanoma cells originating from the neuronal crest lineage and processed of de- or trans-differentiation.

CRISPRa hits and Perturb-seq hits converge to a shared set of ligand–receptor interactions: 53 ligands and receptors interact with both CRISPRa and Perturb-seq resistance hits (*P* = 3.27 × 10^−9^, hypergeometric test) and 16 ligands and receptors interact with both CRISPRa-sensitizing and Perturb-seq-sensitizing hits (*P* = 3.48 × 10^−4^, hypergeometric test). Refining the Perturb-seq-based regulatory network to include all CRISPRa and Perturb-seq hits and integrating ligand–receptor interactions^41–43^ yield a regulatory ligand–receptor network. At the center of this network is a hub of 14 ligand–receptor interactions linking 9 resistance hits (CRISPRa-resistance hits: *CD44*, *COL1A1* and *COL4A3*; Perturb-seq hits: *ITGB8*, *TGFB1* and *NID1*; both CRISPRa and Perturb-seq hits: *SDC2*, *ITGAV* and *HGF*; Fig. 3h). At the center of this hub are collagen–integrin interactions and *CD44*, a stem-cell marker known for its role in cancer cell stemness, self-renewal, tumor initiation and progression^44–46^.

### *MYC* and *SAFB* as regulators of TCR-specific cytotoxicity

**Fig. 4:**
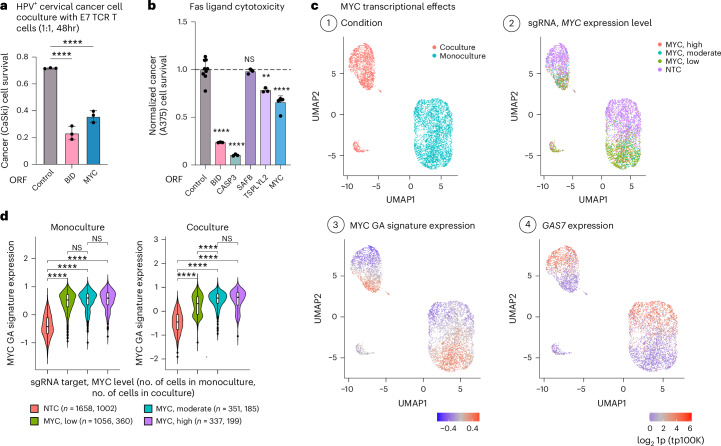
*MYC* and *SAFB* robustly and selectively sensitize cancer cells to TCR-specific cytotoxicity. **a**, *MYC* ORF-based overexpression sensitizes cervical cancer cells to T cell cytotoxicity. The fraction of surviving HPV^+^ cervical cancer cells (*y* axis) in coculture with E7 TCR T cells (1:1, 48 h), is shown for CaSki cells transduced to express a control, *BID* or *MYC* ORF (mean ± s.d., *n* = 3 technical replicates per ORF). ^****^P < 0.001, ordinary one-way ANOVA, Dunnett’s multiple-comparison test. **b**, Fold-change in A375 cell viability after 24-h treatment with FasL (200 ng ml^−1^), shown for A375 cells with ORF-based overexpression of different sensitizing hits compared to A375 cells with a control ORF (mean ± s.d., *n* = 3–6 technical replicates per ORF). ^****^P < 0.0001, ^**^P < 0.01, ordinary one-way ANOVA, Dunnett’s multiple-comparison test, compared to control cells. **c**, UMAP of Perturb-seq data of control (NTC) and *MYC* CRISPRa cells, colored based on: (1) culture conditions, (2) sgRNA and *MYC* expression level, (3) the expression of the MYC GA signature and (4) the expression of the CRISPRa-resistance hit *GAS7* (log_2_1p-transformed tp100k). **d**, Expression of *MYC* GA signature in control (NTC) cells and cells with *MYC* CRISPRa sgRNAs, further stratified based on *MYC* expression in monoculture (left) and coculture (right). The number of cells in each group is shown in parentheses (*n*). Boxplots: the middle line shows the median, the box edges show the 25th and 75th percentiles and the whiskers show the most extreme points that do not exceed ±1.5× the interquartile range (IQR). Further outliers are marked individually with circles (minima or maxima). ^****^P < 0.0001, one-tailed Student’s *t*-test.

To test the hits in vivo, we performed a focused, in vivo CRISPRa screen spanning the 61 genes perturbed in the Perturb-seq screen. We engrafted the pool of CRISPRa A375 cells in immunocompromised (NSG) mice (*n* = 18) and treated a subset of the mice with NY-ESO-1 TCR T cells (Methods). MAGeCK analyses comparing tumors from treated and untreated mice identified five T cell resistance (*BCL2L1*, *CD44*, *E2F1*, *IRF1* and *JUN*) and three sensitizing hits (*SAFB*, *TCF7L2* and *TSPYL2*, BH FDR < 0.05, with *MYC* showing mixed effects; Supplementary Table 8), further supporting the role of the regulators identified here, including *CD44*, the nucleosome assembly protein *TSPYL2* and the chromatin regulator *SAFB*. *MYC* and *SAFB* ORF-based overexpression also sensitized human papilloma virus (HPV)-positive CaSki cells to T cells with a TCR targeting the E7 HPV oncoprotein^47^ (Figs. 2a and 4a and Extended Data Fig. 3), thus generalizing to virally infected cells with a different TCR and a different (viral) antigen.

**Extended Data Fig. 10:**
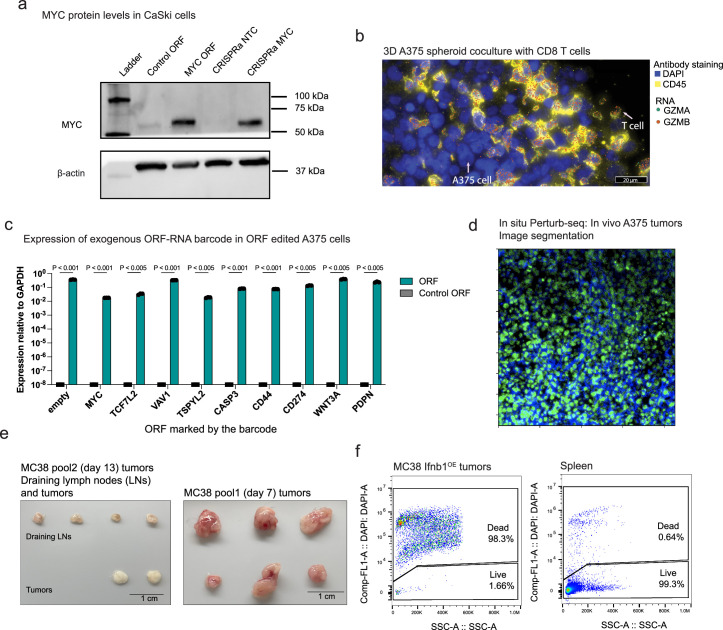
In situ Perturb-seq. (**a**) Representative western blot of MYC protein in CaSki cells transduced to express a control or *MYC* ORF as well as CRISPR/dCas9 CaSki cells with NTC or *MYC* sgRNAs. The results were confirmed in an additional experiment. (**b**) In situ Perturb-seq in 3D coculture of A375 spheroids with CD8 T cells confirms proper detection of CD8 T cells and single RNA molecule detection, as shown from *GZMB* and *GZMA*. This is a representative view of data captured across 78 FOVs. (**c**) qPCR quantification of the ORF-barcodes in A375 transduced with the respective ORF (turquoise) or with a control ORF (grey). Data are presented as mean values (*n* = 3 technical replicates per group). P-values were computed via unpaired one-tailed t-test with BH correction for multiple hypothesis testing. (**d**) Image segmentation of a representative FOV from the A375 in vivo in situ Perturb-seq data. White contours depict the cell boundaries. The data includes 754 FOVs, all segmented and visualized in the same manner. (**e**) Tumor formation patterns in MC38 Pool2 versus Pool1. MC38 Pool2 (left) and Pool1 (right) cells were engrafted into a single flank of C57BL/6 mice and collected at the indicated day post-engraftment. In Pool2, draining inguinal lymph nodes (LNs) were collected (*n* = 4, top) and white tumor masses were observed at the injection site (bottom). In Pool1, typical MC38 tumors were observed and collected (*n* = 6). Scale bars were added after image acquisition based on the diameter of the tissue container. (**f**) Flow cytometry plots of white tumor mass and spleen samples excised from C57BL/6 mice (*n* = 2) inoculated with a single subcutaneous engraftment of Ifnb1^OE^ MC38 cells into the flank and harvested 13 days post-engraftment. Tumor and spleen tissues were processed in parallel. Plots show live (DAPI^−^) versus dead (DAPI^+^) cell populations. Source data

*MYC* is a major oncogene that has been shown to support immune evasion^48–54^. Its sensitizing effects observed here (Figs. 1c,d,g,h and 4a,b and Extended Data Fig. 10a) are aligned with its role as a positive regulator of apoptosis^55,56^. It has been previously suggested that a modest increase in *MYC* expression triggers proliferation, whereas a dramatic increase triggers apoptosis^56^. Yet, at the RNA level, only 1.85% of the cells with *MYC* CRISPRa show *MYC* expression levels higher than those observed in control cells, and similar transcriptional alterations are found in *MYC* CRISPRa cells also on modest or low *MYC* overexpression (*r*_s_ > 0.82, *P* < 1 × 10^−10^, Spearman’s correlation; Fig. 4c,d and Methods). Both high and low *MYC* CRISPRa overexpression significantly upregulated sensitizing CRISPRa hits (*P* = 7.38 × 10^−3^, hypergeometric test; for example, *BAK1*, *BID*, *POLR1A* and/or *POLR3A*, *SAFB* and *WNT3*) and downregulated resistance CRISPRa hits (*P* = 2.27 × 10^−3^, hypergeometric test; for example, *BCL2A1*, *CD109*, *CSF1*, *ETV6*, *FADD*, *MCL1* and *SMARCA2*), most notably *GAS7* (Figs. 3g and 4c,d). In accordance with previous studies^48–54^, *MYC* CRISPRa downregulated antigen presentation genes (human leukocyte antigen (HLA) genes), *STAT1*, *STAT2* and IFNγ response genes also in A375 cells (Supplementary Fig. 10a and Supplementary Table 7a), further indicating that these processes are not limiting TCR-specific cytotoxicity in this context. *MYC* ORF-based overexpression had no significant impact on A375 cell susceptibility to IFNγ and TNF (Supplementary Fig. 10b) but sensitized the cells to Fas ligand (FasL; Fig. 4b). Pan-caspase inhibition rescued *MYC*-overexpressing A375 cells from NY-ESO-1 TCR T cell cytotoxicity (Supplementary Fig. 10c). These findings call for further investigation into the potentially context-dependent role of *MYC* in immune evasion.

*SAFB* (scaffold attachment factor B) emerged as a robust and TCR-specific sensitizing hit (Figs. 1c–h, 2b,c, 3e and 4b). In contrast to *MYC*, *SAFB* CRISPRa overexpression showed no impact on cell proliferation or fitness at baseline (Fig. 1d). *SAFB* encodes a DNA-binding and RNA-binding protein that attaches the base of chromatin loops to the nuclear matrix^57,58^. Although depletion of *SAFB* has been shown to alter 3D genome organization and decrease genomic compartmentalization^57,58^, *SAFB* CRISPRa had minimal impact on the transcriptome of A375 cancer cells based on our Perturb-seq data (Fig. 3e). Validating *SAFB* synthetic lethality, ORF-based overexpression of *SAFB* in A375 cells had no impact on cell viability and proliferation (Fig. 2b) or susceptibility to IFNγ and TNF (Fig. 2c). In contrast to *CASP3*, *BID* and *MYC*, *SAFB* overexpression also had no impact on cancer cell susceptibility to FasL (Fig. 4b). Collectively, these findings demonstrate that SAFB plays a uniquely specific and potentially therapeutically relevant role in facilitating TCR-specific, granzyme- and perforin-based cytotoxicity, manifesting immune RNA-based synthetic lethality.

### In situ Perturb-seq for pooled optical genetic screens

**Fig. 5:**
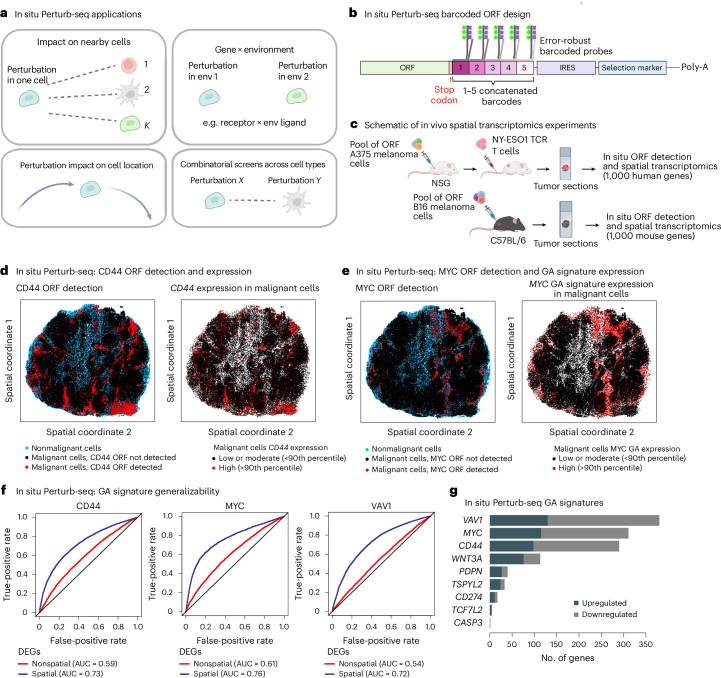
In situ Perturb-seq and its application to in vivo melanoma models. **a**, Schematics of key questions that in situ Perturb-seq can be used to address in a multiplexed manner. **b**, In situ Perturb-seq design. Each ORF or sgRNA is matched to one to five concatenated barcodes, such that each barcode is designed to align an error-robust probe for high-plex detection of different perturbations. **c**, Experimental workflow of in situ Perturb-seq applied here. Top: pooled ORF-expressing A375 melanoma cells engrafted in NSG mice for in situ ORF detection and spatial transcriptomics with a 1,000 human gene panel. Bottom: pooled ORF-expressing B16 melanoma cells engrafted in C57BL/6 mice and profiled for in situ ORF detection and spatial transcriptomics with a 1,000 mouse gene panel. **d**–**g**, In situ Perturb-seq applied to A375 xenografts in NSG mice. **d**, In situ Perturb-seq detection of the genetic perturbation (*CD44* ORF) in the intact tumor tissue matching the expression of the target gene (*CD44* RNA levels). **e**, In situ Perturb-seq detection of *MYC* ORF matching the overall expression of the *MYC* GA signature. **f**, Expression of GA signatures distinguishing between control and perturbed cells in unseen tumors, as shown when defining the GA signature based on differentially expressed genes that are supported (blue) or not supported (red) by spatially aware differential gene expression statistical models. The classification performances are shown based on a leave-one-tumor-out crossvalidation procedure (LOOCV; Supplementary Note 1). **g**, Size of the GA signatures (*x* axis) identified via in situ Perturb-seq for different ORFs (*y* axis). DEGs, differentially expressed genes; env, environment. Illustrations in **b** and **c** created in BioRender; Jerby Lab https://biorender.com/qdelxmb (2026).

To investigate intercellular mechanisms, we developed in situ Perturb-seq for pooled optical genetic screens with in situ detection of genetic perturbations and spatial transcriptomics readouts at single-molecule resolution (Fig. 5a,b, Extended Data Fig. 10b–d and Methods). We first applied in situ Perturb-seq to immunocompromised (NSG) mice with engraftment of A375 cancer cells (Fig. 5c–g). A375 melanoma cells were transduced to overexpress one of the seven top hits (*WNT3A*, *MYC*, *TSPYL2*, *CASP3*, *PDPN*, *CD44* and *TCF7L2*), *CD274* as a positive control, *VAV1* or an empty control ORF with barcode only (Extended Data Fig. 10c). Xenograft tumors containing the pool of genetically modified melanoma cells were engrafted, collected and profiled via CosMx^59^ for detection of 1,000 human genes at single-molecule resolution along with in situ detection of the ORF barcodes (Fig. 5b,c, Methods and Extended Data Fig. 10d).

In total, 950,988 cells were profiled, including 351,915 cancer cells carrying one of the 10 ORFs, confirming robust ORF-based gene overexpression (Fig. 5d). Cancer cells expressing the same ORF were often spatially clustered together as a clone, creating ‘perturbational zonation’ within the tumor and thus mitigating crossperturbation confounding effects (Fig. 5d,e and Methods; *P* < 0.01 for all ORFs, empirical permutation tests). Controlling for spatial location in the statistical model of differential gene expression resulted in more robust in vivo GA signatures (Fig. 5f and Methods). These in situ Perturb-seq GA signatures (Fig. 5g and Supplementary Table 9) significantly overlapped the Perturb-seq-based ones (*P* = 2.16 × 10^−12^, Fisher’s test). The *WNT3A* GA signature supported by both datasets showed enrichment for overexpression of genes involved in embryonic development (for example, *SOX4*, *TWIST1* and *WNT5A*; *P* = 1.95 × 10^−3^) and the *MYC* GA signature was enriched for overexpression of *MYC* target genes (for example, *HSP90AB1*, *PTGES3* and *SRSF2*; *P* = 1.36 × 10^−3^, hypergeometric test; Fig. 5e).

Although a subset of the mice was treated with an NY-ESO-1 TCR T cell injection, only a small number of T cells were detected in the tumors (<0.3% of the cells per tumor), necessitating other model systems to study the perturbation impact on the tumor microenvironment.

### In situ Perturb-seq reveals intercellular mechanisms

**Fig. 6:**
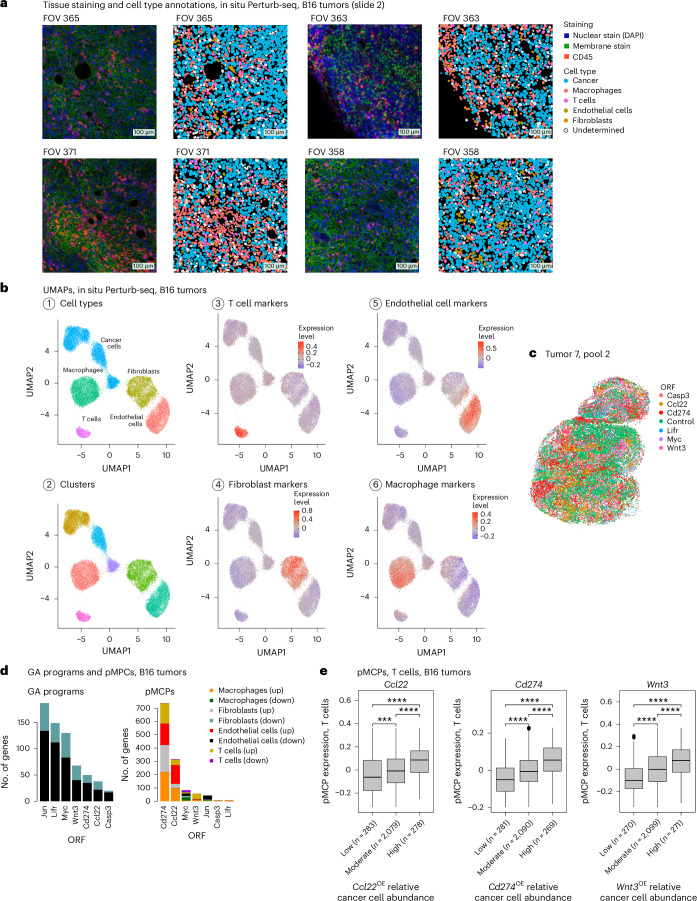
In situ Perturb-seq applied in vivo to syngeneic B16 tumors in immunocompetent mice. **a**, Representative immunofluorescence and matching cell segmentation and cell-type annotations of 4 (out of 637) fields of view (FOVs) from B16 tumors. **b**, UMAP of the B16 in situ Perturb-seq data, with cells colored based on (1) cell-type annotations, (2) cluster assignment and (3–6) expression of cell-type signatures defined in previous single-cell studies^28,67,68^. **c**, A section from a B16 tumor (tumor 7, pool 2). Each dot corresponds to a cancer cell, colored based on ORF detection and plotted on the *x*–*y* spatial coordinates. **d**, GA signatures (left) and pMCPs (right) identified in the B16 in situ Perturb-seq data for the seven ORFs, stratified to upregulated and downregulated genes in the different cell types (Methods). **e**, Expression of the T cell compartment of the *Ccl22* (left), *Cd274* (middle) and *Wnt3* (right) pMCPs in the T cells (*y* axis), stratified based on the relative abundance of cancer cells with the respective ORF in the T cell micro-FOV (*x* axis). ^****^P < 0.0001, one-tailed, unpaired Student’s *t*-test. The number of micro-FOVs is shown in parenthesis per group. Boxplots: the middle line shows the median, the box edges show the 25th and 75th percentiles and the whiskers show the most extreme points that do not exceed ±1.5× the IQR. Further outliers are marked individually with circles (minima or maxima).

In situ Perturb-seq can help us track how a perturbation in one cell impacts other cells around it and how the response of a cell to a perturbation changes as a function of its environment (that is, cell–cell and gene–environment interactions; Fig. 5a). To leverage this, we applied in situ Perturb-seq with a 1,000 mouse gene panel in immunocompetent (C57BL/6) mice engrafted with pooled B16 syngeneic melanoma cells subject to ORF-based overexpression of one of the top hits (*Casp3*, *Ccl22*, *Jun*, *Lifr*, *Myc* and *Wnt3*), *Cd274* or a control ORF (Fig. 6). Tumors were collected on reaching a critical mass (~200 mm^3^) and profiled together.

The data include a total of 503,180 high-quality annotated cells, 446,547 of which were annotated as cancer cells (Fig. 6a–c). To process the data and accurately annotate the cells, we devised a scheme that is based on the generation of high-quality reference maps (Methods), resulting in cell-type-specific clusters annotated as cancer cells, T cells, macrophages, fibroblasts and endothelial cells based on their expression of cell-type markers and signature (Fig. 6a,b). ORF detection matched the overexpression of the target gene (Supplementary Table 10a) and, once again, cancer cells harboring the same ORF co-localized (Fig. 6c and Methods; *P* < 0.001 for all ORFs, empirical permutation tests).

Demonstrating gene–environment interactions, overexpression of the receptor *Lifr* had a different impact on the cancer cell transcriptome depending on the expression levels of its ligands in nearby cells. Lifr^OE^ cells overexpressed acute inflammation and other Lifr-related genes (for example, *Stat3* and *Cxcl1*), overexpressed interleukin-6 (IL-6) production regulators (for example, *Cd36*, *Klf2* and *Nod2*) in microenvironments with high *Clcf1* expression, overexpressed steroid-related genes (for example, *Apoc1*, *Stat5b* and *Tnf*) in microenvironments with high *Osm* expression and overexpressed IFNγ response genes (for example, *Csf2rb*, *Cxcl9* and *Ifi44l*) in microenvironments with high *Lif* expression (*P* < 0.01, mixed-effects models; Methods and Supplementary Table 10b).

Following our previous work, where we demonstrated the concept of multicellular programs (MCPs, that is, different genes across different cell types that are co-expressed when the cells are next to each other in the tissue)^60^, we used in situ Perturb-seq to identify perturbation MCPs (pMCPs), consisting of genes that are differentially expressed in a given cell type on ‘exposure’ to cancer cells with the pretraining perturbation (Fig. 6d, Supplementary Table 10c and Methods). The pMCPs identified in the in situ Perturb-seq B16 data include both cell-type-invariant and cell-type-specific effects, such that the size of the pMCP signature diverges from the size of the matching GA signature, potentially capturing cell-autonomous versus intercellular modes of action (Figs. 5a and 6d).

**Fig. 7:**
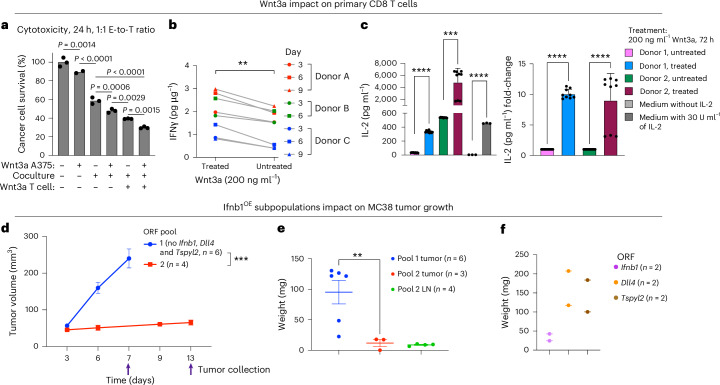
Sensitizing Wnt and IFN hits show noncell autonomous effects. **a**–**c**, Wnt3a impacts T cell cytotoxicity, IFNγ and IL-2 secretion. **a**, A375 cancer cells (top row) or NY-ESO-1 TCR T cells (bottom row) were treated with recombinant human Wnt3a (200 ng ml^−1^) for 24 h. Wnt3a was then washed off, and A375 cells were cocultured or monocultured (middle row) with the NY-ESO-1 TCR T cells (1:1) for 24 h. Cancer cell viability was measured via PrestoBlue (*y* axis). Data are presented as mean values (*n* = 2–3 technical replicates per group, compared via ordinary one-way ANOVA). **b**, Primary CD8 T cells stimulated with CD3 or CD28 beads and treated with recombinant human Wnt3a (200 ng ml^−1^) every 3 d. IFNγ secretion was measured by ELISA. The results are shown for T cells derived from three different donors (*n* = 3 biological replicates per timepoint and treatment group). ^***^P < 0.0001, two-tailed, paired Student’s *t*-test. **c**, Primary CD8 T cells derived from two donors cultured for 72 h with and without treatment with recombinant human Wnt3a (200 ng ml^−1^). IL-2 secretion was measured by ELISA. Data are presented as mean ± s.d. (*n* = 9 technical replicates per condition and donor). ^****^P < 0.0001, ^***^P < 0.001, two-sided, unpaired Welch’s *t*-test. **d**–**f**, *Ifnb1* overexpressing MC38 cancer cells preventing tumor growth and resulting in a white mass even when comprising only 10% of the cancer cell population. **d**, MC38 colorectal cancer cells transduced to overexpress ORFs of sensitizing and resistance hits. The cells were then pooled into two pools, each with ten different ORFs, such that *Ifnb1*, *Dll4* and *Tspyl2* ORFs were included in pool 2 but not in pool 1 (Methods). Tumor volume (mean ± s.e.m., *y* axis) measured at the indicated times (*x* axis) is shown, with arrows marking tumor collection times. ^***^P = 0.0008, two-tailed, unpaired Welch’s *t*-test comparing final tumor volume measurements. **e**, Tumor weight (shown as mean ± s.e.m., *y* axis) was measured post-resection for the tumors shown in **d**. The tumor-draining lymph nodes of tumors from pool 2 were enlarged and were also collected and weighed (Extended Data Fig. 10e). ^**^P = 0.0064, two-tailed, unpaired Welch’s *t*-test. **f**, The final tumor weight of MC38 tumors with ORF-based overexpression of a single sensitizing hit: *Dll4*, *Ifnb1* or *Tspyl2* (*n* = 2 per hit). Source data

*Cd274* (encoding PD-L1), *Ccl22* and *Wnt3* overexpression in the cancer cells was associated with shifts in the state of nearby immune and stromal cells, including T cells (Fig. 6d,e). As expected, T cell dysfunction genes were overexpressed in T cells residing in proximity to Cd274^OE^ B16 cancer cells (*P* = 8.15 × 10^−4^, hypergeometric test). T cell activation markers, including the co-stimulatory gene *Cd28*, were overexpressed in T cells in proximity to Wnt3^OE^ cancer cells (Fig. 6e). To test this, we performed ex vivo experiments and found that, indeed, exposure of primary human CD8 T cells to soluble Wnt3a significantly increased T cell cytotoxicity (Fig. 7a), IFNγ secretion (both at baseline and under chronic activation, Fig. 7b) and IL-2 secretion (more than a ninefold increase; Fig. 7c). This demonstrates how in situ Perturb-seq can help uncover intercellular regulation and ways to reinvigorate T cells via sensitizing hits identified in cancer cells.

## Discussion

Existing immunotherapies can be very potent but are limited in the face of cancer cell intrinsic resistance and involve the risk of mild-to-severe adverse effects^7,8,61^. Here we report distinct mechanisms and genes more exclusively involved in TCR-specific cytotoxicity, laying the groundwork for more precise interventions to selectively eliminate cancer and potentially other dysfunctional cells. With a focus on transcriptional activation, we identified a suite of previously underappreciated and functionally diverse genes that control cancer cell susceptibility to TCR-specific cytotoxicity and introduced a scalable approach to decode the function of such regulators by combining highly controlled CRISPRa screens with Perturb-seq and in situ Perturb-seq.

In situ Perturb-seq provides a high-throughput method to study multicellular regulation and gene–cell–environment interactions in this and other contexts. It supports high-plex testing of dozens of different perturbations at a time and can be used to perform combinatorial screens across different cell types with genetic perturbations applied to more than one cell type (Fig. 5a).

The immunomodulators that we identified can provide leads for interventions as targeted RNA or small interfering RNA delivery and cancer gene therapy^62–64^. In certain cases (for example, *CASP3*, *SAFB* and *TSPYL2*), the impact is expected to be primarily on the cells receiving the molecular cargo, whereas, in other cases (for example, *IFNB1* and Wnt ligands), drug delivery to a subset of cancer cells may unleash an immune response against the whole tumor. Supporting this, we find that it is sufficient to have only 10% of cancer cells overexpressing the sensitizing hit *Ifnb1* to trigger a substantial enlargement of the tumor-draining lymph nodes and completely prevent MC38 tumor growth in immunocompetent mice (Fig. 7d–f and Extended Data Fig. 10e,f).

By linking hits identified in cancer cells to either cognate receptors on the T cells or T cell transcriptional states using in situ Perturb-seq, we can identify T cell perturbations that enhance T cell functionality. The impact that Wnt3a has on T cells demonstrates the potential of this approach and opens directions for ex vivo production of T cell therapies with Wnt supplements or engineering T cells to express Wnt ligands or synthetically modified Wnt receptors.

As for study limitations, although we validated specific hits in HPV^+^ cells using a different TCR and antigen, repeating the CRISPRa screens conducted here in additional types of cancer and noncancerous cells will be important to determine hit generalizability. Further investigation is required to delineate the molecular mechanisms and therapeutic potential of top hits.

We anticipate that the study will catalyze additional CRISPRa screens focused on precise interrogation of TCR-specific cytotoxicity across cellular contexts, as well as mechanistic studies and combinatorial and protein engineering screens focused on the top hits identified here. The work presents a systematic stepwise approach to uncover and decode gene function across scales, identifying and leveraging context-specific effects of gene activation to restore TCR-specific cytotoxicity.

## Methods

### Ethical regulation

The study received institutional regulatory approvals. All experiments were conducted in accordance with Stanford University Environment, Health and Safety Committee guidelines and approved under protocol nos. APB-3910-L.J.0921 and APB-5416-L.J.0724.

All research using human-derived materials was performed under Stanford University Institutional Review Board (IRB) approval (protocol no. IRB-13942). De-identified buffy coats were received from the Stanford Blood Center (IRB protocol no. 13942). The Stanford Blood Center operates in a fully Health Insurance Portability and Accountability Act-compliant manner, adhering to privacy regulations and Department of Health and Human Services regulatory obligations.

All animal experiments were carried out in accordance with Stanford University Institutional Animal Care and Use Committee policies and approved under protocol no. APLAC-34218. The maximal permitted tumor burden was defined as a tumor diameter not exceeding 17 mm in adult mice, with earlier euthanasia required if animals showed signs of predefined clinical and morbidity criteria. In all in vivo experiments conducted in this study, animals were closely monitored throughout the study and approved tumor burden limits were not exceeded. Immunocompromised mice were housed in a -pathogen-free facility. All mice were housed under controlled environmental conditions, in 12-h light-to-dark cycles at temperatures ranging from 20 °C to 26 °C and humidities between 30% and 70%.

### Cell culture

The melanoma cell line A375 and breast adenocarcinoma cell line MCF7 were obtained from American Type Culture Collection (ATCC, cat. no. CRL-1619) and Sigma-Aldrich (cat. no. 86012803), respectively. A375 and MCF7 cells were cultured in Dulbecco’s modified Eagle’s medium (DMEM) high glucose (Sigma-Aldrich, cat. no. D6429) supplemented with 10% fetal bovine serum (FBS; Life Technologies, cat. no. A3840102) and 1× penicillin–streptomycin (Pen–Strep; Cytiva, cat. no. SV30010). The cervical cancer cell line CaSki (ATCC, cat. no. CRL-1550) was cultured in Roswell Park Memorial Institute (RPMI) medium with GlutaMAX (Gibco, cat. no. 72400-047) supplemented with 10% FBS and 1× Pen–Strep. The Lenti-X HEK293T cell line was obtained from Takara Bio (cat. no. 632180) and cultured in DMEM high glucose supplemented with 10% FBS. Peripheral blood mononuclear cells were obtained from buffy coats (Stanford Blood Center) by Ficoll-Paque (Cytiva, cat. no. 17544202) in SepMate Tubes (STEMCELL Technologies, cat. no. 85450) following the manufacturer’s protocol. CD8^+^ T cells were isolated from peripheral blood mononuclear cells using immunomagnetic negative selection (STEMCELL Technologies, cat. no. 17953) and cultured in RPMI-1640 medium with GlutaMAX and Hepes (Gibco, cat. no. 72400-047), supplemented with 10% FBS, 1% human serum type AB (Sigma-Aldrich, H4522), 5 mM MEM Non-Essential Amino Acids (Corning, cat. no. 25-025-Cl), 5 mM sodium pyruvate (Corning, cat. no. 25-000-Cl), 50 μM 2-mercaptoethanol (Sigma-Aldrich, cat. no. M6250), and 30 U of recombinant IL-2 (National Institutes of Health (NIH)). The B16-OVA MO4 cell line (Sigma-Aldrich, cat. no. SCC420) was cultured in RPMI-1640 medium supplemented with 10% FBS, 5 mM MEM Non-Essential Amino Acids, 50 μM 2-mercaptoethanol and 1 mg ml^−1^ of geneticin (Invivogen, cat. no. ant-gn-5). The MC38 cell line (Sigma-Aldrich, cat. no. SCC172) was cultured in DMEM high glucose supplemented with 10% FBS, 5 mM Non-Essential Amino Acids, 1 mM sodium pyruvate and 50 μg ml^−1^ of gentamicin sulfate (Sigma-Aldrich, cat. no. G1272). All cell lines were authenticated using ATCC by simple tandem repeat profiling. All cells were regularly tested for *Mycoplasma* spp. and confirmed negative (Boca Scientific, cat. no. 25235).

### TCR engineering

Primary CD8 T cells were transduced to express specific TCRs to model TCR-specific cytotoxicity.

For melanoma studies the TCR recognizing the HLA-A*-02-restricted cancer testis antigen NY-ESO-1 (NY-ESO-1:157–165 epitope) was used^23^ (Extended Data Fig. 1 and Supplementary Fig. 11a,b). A lentiviral plasmid containing the TCR with a hemagglutinin (HA)-tag and protein C (PC)-tag marking the α and β chains, respectively (Extended Data Fig. 1b), was generously provided by K. Wucherpfennig (Dana-Farber Cancer Institute)^69^. Then, 1 d after stimulation with CD3 or CD28 dynabeads, 2 × 10^6^ T cells were transduced with 300 μl of concentrated lentivirus and expanded for 3 d. Cells were then labeled with anti-HA and anti-PC antibodies and sorted for double-positive cells (Extended Data Fig. 1c and Supplementary Table 11). The sorted cells were expanded and used for experiments or frozen down for later use. TCR functionality was validated via peptide–MHC tetramers obtained from the NIH Tetramer Facility (Supplementary Note 1 and Extended Data Fig. 1d) and TCR-dependent cytotoxicity in coculture with A375 cells (Fig. 1b).

To test the findings in a different model with a different antigen and a different TCR, HPV^+^ CaSki cells and E7 TCR primary CD8 T cells were used. E7 TCR T cells were generated by transducing primary CD8 T cells to express a high-avidity TCR that targets HPV16 E7 antigen through recognition of the E7_11–19_ epitope complexed with HLA-A*02:01 (refs. ^47,70^). The expression of HPV E7 protein and HLA-A2 surface protein in CaSki cells was confirmed by western blotting and flow cytometry, respectively (Supplementary Note 1, Extended Data Fig. 3a,b, Supplementary Fig. 11c and Supplementary Table 11). A retroviral plasmid carrying the E7 TCR construct was obtained from Addgene (cat. no. 122728; Extended Data Fig. 3c). T cells were transduced with concentrated retrovirus 1 d after stimulation. Transduced T cells were then sorted using an anti-mouse TCR β chain antibody (BioLegend, cat. no. 109215) to sort E7 TCR T cells (Extended Data Fig. 3d and Supplementary Fig. 11d). The sorted cells were confirmed to have E7 TCR-specific cytotoxicity in coculture with CaSki cells (Extended Data Fig. 3e).

### In vitro 2D coculture assays

Coculture assays were performed in either 96-well or 24-well plates. For the 96-well plate, 1 × 10^4^ or 2 × 10^4^ cells were seeded in 100 μl of growth medium per well. For the 24-well plate, 5 × 10^4^ or 1.2 × 10^5^ cells were seeded in 500 μl of growth medium per well. Then, 4 h after seeding, CD8 T cells were added in T cell medium at the appropriate E-to-T ratio and cocultured for 24 h, 48 h or 72 h. T cells were washed with phosphate-buffered saline (PBS) and cancer cell viability was measured with PrestoBlue using the Infinite M1000 plate reader (Tecan). Cell survival was computed by normalizing to the signal obtained in monoculture or in coculture, with nonspecific T cells obtained from the same donor when using the same cancer cell line with the same genetic perturbation.

### Coculture CRISPRa screens

An A375 cell line stably expressing dCas9-VPR was established as follows: A375 cells were transduced with a lentiviral construct containing dCas9-VPR-t2a-GFP at a multiplicity of infection (MOI) of ~0.2. GFP^high^ or GFP^low^ cells were sorted as single cells into two 96-well plates. The activity of dCas9-VPR in clones was assessed by transducing cells with a *CD2* sgRNA and examining CD2 expression by qPCR and flow cytometry (Supplementary Table 11). A clone was selected for future experiments based on high induction of CD2 expression (Extended Data Fig. 1h,i and Supplementary Fig. 11b) as well as similar growth characteristics to wild-type A375 cells.

Pooled lentivirus sgRNA library (Addgene, cat. no. 83981) targeting a total of 2,921 genes, with a total of 32,670 sgRNAs (10 sgRNAs per gene and 560 control gRNAs; Supplementary Table 1), was amplified via liquid culture, then transduced into dCas9-VPR-expressing A375 cells at an MOI < 0.3. Cells were selected with puromycin (1 μg ml^−1^) for 3 d, then allowed to recover and expand for 7 d before freezing at 20 × 10^6^ cells per vial.

CRISPRa screens were conducted with A375 cells under two coculture conditions: (1) coculture with wild-type CD8 T cells and (2) coculture with NY-ESO-1 TCR CD8 T cells obtained from the same donor. In all cases an E-to-T ratio of 0.25:1 was used. Before adding the T cells, 20 × 10^6^ library cells were seeded into T-225 flasks for 4 h. In the first screen, A375 cells were cocultured with either NY-ESO-1 TCR or wild-type T cells for 24 h before T cells were removed by washing twice with PBS. In the second screen, A375 cells were subject to three rounds of coculture with either NY-ESO-1 TCR or wild-type T cells at a 0.25:1 E-to-T ratio, with 5 d of recovery between each round (Extended Data Fig. 1a). A coverage of 500× of the library was maintained throughout the screens. Both screens were performed in triplicate.

Genomic DNA (gDNA) was extracted with the DNA Blood Maxi Kit (QIAGEN, cat. no. 51194) according to the manufacturer’s protocol. The library was prepared for sequencing following established protocols^71^, as described here. Briefly, sgRNAs were captured and amplified from the gDNA samples using two rounds of PCR. After the second PCR, the products were gel purified and verified pure via a bioanalyzer. PCRs were run on the VeritiPro 96-well Thermocycler (Applied Biosystems). Samples were sequenced using the Illumina NextSeq 550 with >200× library read coverage per sample.

### Coculture CRISPRa screen data analyses

CRISPRa screen data were analyzed using MAGeCK (v0.5.9.4)^30^. Fastq files were processed to align the reads to the sgRNA spacer sequences, generating a counts matrix of sgRNA by samples. The variance of read counts was estimated by fitting a negative binomial (NB) across all sgRNAs, using the NTC sgRNAs to normalize the data and mitigate technical variation across the samples. The resulting NB model was then used to test whether the abundance of sgRNAs targeting a given gene differs significantly between coculture with NY-ESO-1 TCR and coculture with wild-type T cells.

Here tests were performed for each screen separately. In the case of the second screen, the comparisons were done in a paired time-matched manner, namely comparing the first, second and third challenges with NY-ESO-1 TCR T cells to the first, second and third challenges with wild-type T cells, respectively. The pairwise tests across the different screens were combined with Fisher’s *P* values as the summary statistics and corrected for multiple hypothesis testing using the Benjamini–Hochberg correction. Only genes that were consistently observed as hits (BH FDR < 0.05) were considered as top sensitizing or desensitizing hits.

### *CASP3* mRNA delivery

DNA templates containing the T7 promoter were generated via PCR from a plasmid and then converted into messenger RNA using the HiScribe T7 ARCA mRNA Kit (NEB, cat. no. E2060S) and purified (NEB, cat. no. T2050S). A day before transfection, cells were seeded at 80% confluency. On the day of transfection, mRNA was mixed with Lipofectamine MessengerMAX (Thermo Fisher Scientific, cat. no. LMRNA001) or TransIT-mRNA Transfection Kit (Mirus Bio, cat. no. 2225) and introduced into cells following the manufacturer’s protocol. Coculture experiments were performed in the presence of the RNA-carrying particles.

### Perturb-seq screen

The Perturb-seq sgRNA library, consisting of 276 CRISPRa sgRNA (~4 sgRNAs per gene, targeting a total of 61 genes, and 32 NTC sgRNAs; Supplementary Table 3), was cloned into the pMCB320 backbone and transduced via lentivirus into A375 dCas9-VPR cells at an MOI < 0.3. Transduced cells were seeded at 5 × 10^5^ cells per well in 6-well plates, then either cocultured with NY-ESO-1 TCR T cells at an E-to-T ratio of 0.5:1 or monocultured for 24 h. Cells were harvested at ~1,000 cells μl^−1^, then loaded on to the Chromium chip with 10,000 cells per condition. DNA libraries were prepared following the manufacturer’s protocol (10X Genomics, CG000511 Rev D). Libraries were sequenced with the NextSeq 550 at a 10:1 ratio of gene expression to CRISPR libraries. Two independent Perturb-seq experiments were performed, with independent lentiviral transduction of A375 cells.

### Perturb-seq data processing

Perturb-seq data were processed similarly to previous studies^67,72^, as described hereinafter. Reads were aligned to the human genome (GRCh38) and the sgRNA spacer sequences to convert fastq files to count matrices via the cellranger pipeline (10x Genomics Cell Ranger 7.1.0). Each sample was represented by two count matrices of (1) gene expression (gene by cell) and (2) guide detection (sgRNA by cell). Raw gene expression counts denote the number of unique molecular identifiers matched to a specific gene and unique cell identifier. The raw gene expression counts were converted to tp10k values and log_2_1p-transformed. Default cutoffs were used to remove technical noise from the guide detection matrix to avoid false sgRNA detection. Cells with more than one sgRNA were removed and not used in downstream analyses. Gene expression profiles were clustered using the Seurat (v5.3.1) and SeuratObjection (v5.2.0) R packages, as follows. The 5,000 topmost variable genes were identified via the ‘FindVariableFeatures’ Seurat function. The first 20 principal components (PCs) were computed based on the log1p-transformed tp10k values of the 5,000 topmost variable genes. A shared nearest neighbor graph was constructed (*k* = 10) based on these 20 PCs (‘FindNeighbors’ Seurat function with the default parameters). The original Louvain algorithm^73^ was applied to cluster the cells based on the shared nearest neighbor graph (Seurat ‘FindClusters’ function, resolution = 0.4 and default parameters). A CD8 T cell cluster was identified in the coculture conditions (with expression of *CD3*, *CD8A* and/or *CD8B* and other canonical T cell markers) and was removed from downstream analyses.

### Perturb-seq data differential gene expression analyses

Differential gene expression analyses of Perturb-seq data were performed using edgeR^37,38^ applied to pseudobulk samples generated by aggregating the expression of all the cells carrying the same sgRNA in the same condition (monoculture or coculture) and sequencing batch, resulting in six pseudobulk profiles per sgRNA. Using the edgeR R package (v3.40.2), a quasi-likelihood NB generalized log-linear model was fit to the pseudobulk count data while controlling for the sequencing batch: *y* ~ batch + *x*, where *y* denotes the pseudobulk expression of the gene examined and *x* denotes the groups to be compared (for example, control versus perturbed cells, coculture versus monoculture cells).

Cells carrying one of the NTC sgRNAs were considered to be control cells. Cells were considered to have a specific target gene activated only based on the detection of the respective sgRNA, as described above. GA signatures were identified for each of the 61 target genes by identifying genes that were differentially expressed in the cells with the respective gene activated compared to the control cells, in either the coculture or the monoculture conditions (FDR < 0.01). sgRNA signatures were identified in the same manner when considering only cells with the pertaining sgRNA compared to control cells (FDR < 0.01). Genes differentially expressed in coculture relative to monoculture were identified considering only the control cells (FDR < 0.01).

### Perturb-seq screen quality control and validations

In addition to on-target CRISPRa sgRNA-driven gene overexpression, sgRNAs were tested based on the consistency of the transcriptional shifts observed with different sgRNAs targeting the same gene.

#### SgRNA *z*-scores correlation

The *z*-scores for each sgRNA in each condition were computed following a previously developed procedure^74^ described below:

$${Z}_{k}=\frac{1}{{JI}}\mathop{\sum }\limits_{j=1}^{J}\mathop{\sum }\limits_{i=1}^{I}\left({P}_{k,j,i}-{C}_{j,i}\right)$$

where *P*_*k,j,i*_ is the pseudobulk profile of the cells with sgRNA *k* in condition *i* and sequencing batch *j* and *C*_*j,i*_ is the pseudobulk profile of the control cells in condition *i* and sequencing batch *j*. Pseudobulk profiles of a set of cells were computed as the average log1p-transformed tp100k of these cells. The sgRNA *z*-scores thus depict the deviation in gene expression compared to the control cells while controlling for batch effects. Pairwise sgRNA-to-sgRNA *z*-score, Pearson’s correlation coefficients (*r*) and *P* values were computed for every pair of sgRNAs. Wilcoxon’s rank-sum tests were conducted to compare the coefficients of sgRNA pairs targeting the same gene to those of sgRNA pairs targeting different genes and to those of NTC sgRNA pairs (Fig. 3b).

#### Leave-one-sgRNA-out crossvalidations

A LOOCV procedure was devised where, in each iteration, all the cells expressing one ‘left-out’ sgRNA were excluded from the analysis and the GA signature for the gene targeted by the left-out sgRNA was identified based on its other sgRNAs. The expression of each of the LOOCV GA signatures in the left-out sgRNA and control cells was computed and compared via a one-sided Student’s *t*-test. A univariate classifier that predicts cells as being subject to the perturbation if they have a high expression of the LOOCV GA signatures was tested on the left-out sgRNA cells versus control cells, with an increasing cutoff to generate an ROC curve. The AUROC was computed to evaluate prediction performances.

### CRISPR–Perturb-seq-based regulatory network analyses

The Perturb-seq data were used to generate a regulatory network with nodes denoting genes and directed signed edges *<A,B*,1*>* and *<A,B*,−1*>* denoting that CRISPRa of gene *A* results in upregulation and downregulation of gene *B*, respectively.

The network was then pruned to include the CRISPRa target genes and ‘Perturb-seq hits’, that is, genes that show a consistent link to either the positive or the negative regulation of the phenotype (that is, TCR-specific cytotoxicity).

More formally, Perturb-seq hits were defined by computing two scores for each gene *g*: *S*_sen*,g*_ = Sen_up,*g*_ + Res_down,*g*_ and *S*_res,*g*_ = Res_up,*g*_ + Sen_down,*g*_. Sen_up,*g*_ and Sen_down,*g*_ denote the number of CRISPRa-sensitizing hits that upregulate and downregulate *g*, respectively. Likewise, Res_up,*g*_ and Res_down,*g*_ denote the number of CRISPRa-resistance hits that upregulate and downregulate *g*, respectively. Gene upregulation and downregulation were based on the Perturb-seq data edgeR differential gene expression analyses. A gene was identified as a Perturb-seq sensitizing hit if *S*_sen,*g*_ ≥ 2 and *S*_res,*g*_ = 0 and as a Perturb-seq resistance hit if *S*_res,*g*_ ≥ 2 and *S*_sen,*g*_ = 0. The regulatory interactions between genes that are both CRISPRa and Perturb-seq hits were visualized (Fig. 3g).

All the nodes in the pruned network were then connected to their known ligands or receptors based on 2,678 predefined ligand–receptor pairs^41–43^. The binding pairs of sensitizing CRISPRa hits were compared to the binding pairs of sensitizing Perturb-seq hits and tested for enrichment via hypergeometric tests that consider only genes within the reference ligand–receptor pairs. The CRISPRa hits, Perturb-seq hits and ligand–receptors that bind to hits comprise the Perturb-seq-based regulatory-signaling network. Dense subnetworks connected to multiple hits provide multiple lines of evidence supporting the role of a gene in TCR-specific cytotoxicity (Fig. 3g,h).

### In vivo CRISPRa screen

Two in vivo CRISPRa screens were conducted using male NSG mice aged 7–8 weeks (NOD.Cg-*Prkdc*^*scid*^ *l2rg*^*tm1Wjl*^/SzJ) purchased from the Jackson Laboratory (cat. no. 005557) with NY-ESO-1 TCR T cells derived from two independent donors.

In both screens, A375 dCas9-VPR cells transduced to express the Perturb-seq sgRNA library were injected subcutaneously (screen 1: *n* = 12, 1 × 10^6^ cells; screen 2: *n* = 6, 2 × 10^6^ cells). A subset of the mice was then treated with human NY-ESO-1 TCR T cells injected via the tail vein 10 d (screen 1: *n* = 2, 7 × 10^6^ cells) or 7 d (screen 1: *n* = 3, 4 × 10^6^ cells; screen 2: *n* = 3, 5 × 10^6^ cells) post-engraftment. All tumors were harvested 5–7 d after T cell injection, with tumors from matching untreated control mice harvested at the same time post-engraftment for comparison. In the second screen, library cells were also cultured in vitro in parallel and for the same duration (13 d) as the engrafted cells for comparison. Tumors were dissociated, cell suspensions were washed once with PBS, gDNA was extracted via the DNA Blood Maxi Kit and library preparation was performed as described above. MAGeCK (v0.5.9.4)^30^ was used to compare the treated group to the matching untreated group from the same screen and collection time and to the in vitro samples. *P* values were combined with Fisher’s *P* values as the summary statistics and corrected for multiple hypothesis testing using the Benjamini–Hochberg correction to identify the final hits (BH FDR < 0.05).

### In vivo in situ Perturb-seq screen

To generate barcoded ORFs, gene fragments were synthesized (Twist Bioscience) and cloned into the pLV-EF1a-IRES-Puro backbone using NEBuilder HiFi DNA Assembly (NEB, cat. no. E5520S). Barcodes were designed as the reverse complement of CosMx probes. Lentivirus for each construct was generated (Supplementary Note 1) and transduced into A375 cells at an MOI of ~1. All barcoded ORF cells were pooled together in even proportions. Female hIL2-NOG mice aged 6–8 weeks (NOD.Cg-*Prkdc*^*scid*^ *Il2rg*^*tm1Sug*^ Tg(CMV-IL2)4-2Jic/JicTac, immunocompromised mice that express the human IL-2 cytokine, *n* = 4) were obtained from Taconic (cat. no. 13440-F). Mice were subcutaneously engrafted with 1 × 10^6^ pooled barcoded ORF cells. Then, 12 d post-engraftment, 3 out of the 4 mice were intravenously injected with 2.5 × 10^6^ (*n* = 1) or 5 × 10^6^ (*n* = 2) NY-ESO-1 TCR T cells; 7 d after the T cell injection, mice were euthanized by CO_2_ inhalation. Tumors were harvested, formalin fixed, paraffin embedded and sectioned. Tissue sections were profiled for spatial transcriptomics via CosMx platform with the 1,000-plex panel Human Universal Cell Characterization RNA, following the manufacturer’s protocol (no. MAN-10159-02).

To perform in vivo–in situ Perturb-seq in immunocompetent mice, barcoded mouse ORF constructs were generated as described above. B16-OVA cells were individually transduced with the ORF lentivirus at an MOI ~ 1 for 24 h. After 24 h, the virus was removed and the cells were selected with 1 μg ml^−1^ of puromycin for 3 d (Invivogen, cat. no. ant-pr-1). The cells were then pooled and 1 × 10^6^ cells were injected subcutaneously into a single flank of female C57BL/6J mice aged 13–14 weeks (*n* = 7; Jackson Laboratory, cat. no. 000664). Tumors were collected when reaching ~250 mm^3^ (9–11 d), and then formalin fixed, paraffin embedded and sectioned. Tissue sections were profiled for spatial transcriptomics via CosMx platform with CosMx Mouse Universal Cell Characterization RNA panel according to the manufacturer’s protocol (no. MAN-10184-05).

### In situ Perturb-seq data processing and analyses

Whole-cell segmentation was performed using a deep learning-based, segmentation image processing algorithm (Mesmer^75^) applied to immunofluorescent images of DAPI and CD298–b2m for nuclear and cell membrane detection, respectively. Transcript coordinates were projected on to the cell segmentation maps to generate a cell-by-gene count matrix with matching spatial coordinates for each cell. Counts were converted to transcript per million values. Cells were annotated as carrying an ORF based on the ORF barcode detection.

In the NSG in situ Perturb-seq screen, the CosMx probes used target human genes. Cells were hence annotated as A375 cancer cells, CD8 T cells or mouse cells based on Louvain gene expression clustering and ORF detection. Mouse cells were removed from further analyses. To identify T cells, the expression of a previously defined T cell signature was computed. To enrich for T cells, a reference map was generated from a subset of the data that included 2,000 cells sampled from each cluster in each slide and all cells with a T cell signature expression >0.3. The reference map was clustered and embedded as described in above, but using all genes instead of only highly variable ones. All cells in the data were projected to the reference map using the Seurat functions ‘FindTransferAnchors’ and ‘MapQuery’. All cells assigned to the T cell cluster were annotated as T cells.

B16 in situ Perturb-seq data processing was conducted by generating a reference map from high-quality cells. High-quality cells were defined as those that are repeatedly assigned to the same cluster across multiple subsamples of the data. The reference map was clustered and embedded as described above, but using all genes instead of only highly variable ones. Cluster-specific markers were defined as genes overexpressed in the pertaining cluster compared to all other clusters (*P* < 0.05, Student’s *t*-test, and log_2_(fold-change) >0.25). Clusters were assigned to cell types based on the enrichment of cluster-specific markers with previously defined cell-type signatures^28,67,68^. All cells in the B16 Perturb-seq data were projected to the reference map using the Seurat functions ‘FindTransferAnchors’ and ‘MapQuery’. Cells that were projected to the reference map with a high confidence score (>0.8) were retained for downstream analyses.

### Identifying GA signatures with and without spatial covariates

Mixed-effect models were used to identify GA signatures while accounting for cell location (BH FDR < 0.1, mixed-effects test). To identify the GA signature of a specific ORF, only the cancer cells expressing the pertaining ORF and control cancer cells were considered. Considering only these cancer cells, the following model was fit to each gene: ‘*y* ~ (1|FOV) + ORF + complexity’, where *y* denotes the expression of the gene across the cells, FOV is the field of view that each cell was assigned to, ORF denotes whether the cells express the ORF barcode, and complexity denotes the total number of genes detected in each of the cells. The lme4 (v1.1-35.4)^76^ and lmerTest (v3.1-3) R packages^77^ were used to fit the models using the restricted maximum likelihood method and to compute *P* values.

To examine whether spatial information improves the detection of GA signatures, GA signatures were also identified via the Model-based Analysis of Single-cell Transcriptomics (MAST) test^78^—a hurdle model tailored to scRNA-seq data using the MAST R package (v1.36.0). Genes that were only supported by the MAST model were considered as the nonspatial GA signature. A LOOCV procedure was conducted, where each time one of the tumors was left out and the spatial and nonspatial GA signatures were identified based on the data of three out of the four A375 tumors and tested on the left-out tumor. The expression of each GA signature was computed per cell and AUROCs were computed to determine whether the signatures were predictive of the cells with the target gene ORF versus control ORF in the unseen test tumor.

### Perturbation MCPs and gene–environment interactions

Perturbation MCPs were identified by generating pseudobulk profiles per cell type per micro-FOV and computing the ‘perturbation abundance’ in each micro-FOV, defined as the fraction of cancer cells with the perturbation. Partial Spearman’s correlation was computed for each cell type to identify genes that are positively and significantly (BH FDR < 0.1, Spearman’s correlation) correlated with the abundance of each perturbation while controlling for the abundance of other perturbations.

To identify gene–environment interactions between *Lifr* ORF-based overexpression and its ligands in the microenvironment, the following mixed-effects model was fit:

$$y \sim (1|\mathrm{FOV})+\mathrm{comp}+x+\mathop{\sum }\limits_{i}{L}_{i}+\mathop{\sum }\limits_{i}{L}_{i}x$$

where *x* denotes whether the cell expressed the *Lifr* ORF, *L*_*i*_ denotes whether ligand *i* (*Clcf1*, *Osm* or *Lifr*) is expressed at a high level (>median) in the micro-FOV and *L*_*i*_*x* is the interaction term between the two. Of note, this model can also be used to quantify the transcriptional response to any other perturbation *x* as a function of microenvironmental factors represented by *L*_*i*_.

### In vivo study of hits in MC38 syngeneic tumors

C57BL/6NCrl mice (Charles River Laboratories, cat. no. 027) were used to test the hits in the immunogenic MC38 syngeneic tumor model. MC38 cells were transduced with lentivirus of barcoded ORFs at an MOI ~ 1 with 3 μg ml^−1^ of polybrene (MilliporeSigma, cat. no. TR-1003) for 48 h. After 48 h, the virus was removed and the cells were selected with 5 μg ml^−1^ of puromycin for 4 d (Invivogen, cat. no. ant-pr-1). The cells were pooled into two pools: (1) control plasmid (no ORF, only barcode), *Cd274*, *Ccl22*, *Casp3*, *Cd44*, *Wnt3*, *Jun*, *Lifr*, *Myc* and *Safb* (*n* = 6 mice); and (2) control plasmid, *Dll4*, *Ifnb1*, *Tspyl2*, *Cd44*, *Wnt3*, *Jun*, *Lifr*, *Myc* and *Safb* (*n* = 4). Cells, 3 × 10^6^, from each pool were injected subcutaneously into a single flank of male mice aged 9–10 weeks. Tumors were harvested when reaching ~250–300 mm^3^ between days 6 and 10 or on day 13 if they did not reach the target size. As pool 2 did not form tumors in the same manner, the experiment described above was repeated with separate engraftment of 3 × 10^6^ MC38 cells of the Ifnb1^OE^, Tspyl2^OE^ and Dll4^OE^ lines into a single flank of female mice aged 11–12 weeks (*n* = 2 per ORF). Tumors were harvested, weighed and processed for flow cytometry (Supplementary Note 1, Supplementary Fig. 11e and Supplementary Table 11).

### Statistics and reproducibility

Statistical significance was assessed as indicated in the accompanying text or figure legend. Depending on the experimental design, Student’s and Welch’s *t*-tests or ANOVA with either Šídák’s or Dunnett’s multiple-comparison tests were performed to compare unimodal readouts (for example, cytotoxicity, cytokine secretion) across different experimental groups. As described in detail above, CRISPR screen data were analyzed via MAGeCK^30^ and differential gene expression analyses of Perturb-Seq and in situ Perturb-Seq data were performed using edgeR^38^ and mixed-effect models, respectively. Unless indicated otherwise the Benjamini–Hochberg correction was used to control for multiple hypothesis testing and to determine the FDR. No statistical method was used to predetermine sample size. No data were excluded from the analyses other than in downstream Perturb-seq analyses that were performed only with cells that pass quality control criteria as described above. Investigators were not blinded to experimental group allocation in experiments validating or investigating specific hits or treatments. The bulk of the study was performed via multiplexed experiments and screens, which are inherently randomized, such that the investigators were blinded to the allocation of cells to experimental groups.

### Reporting summary

Further information on research design is available in the Nature Portfolio Reporting Summary linked to this article.

## Online content

Any methods, additional references, Nature Portfolio reporting summaries, source data, extended data, supplementary information, acknowledgements, peer review information; details of author contributions and competing interests; and statements of data and code availability are available at 10.1038/s41588-026-02561-7.

## Supplementary information

Supplementary Information

Reporting Summary

Supplementary Tables Supplementary Tables 1–11 (description of each table is provided in Supplementary Information).

## Source data

Source Data Fig. 2 Unprocessed western blot.

Source Data Fig. 7 Statistical source data.

Source Data Extended Data Fig. 1 Unprocessed western blot.

Source Data Extended Data Fig. 3 Unprocessed western blot.

Source Data Extended Data Fig. 10 Unprocessed western blot.

## Data Availability

The data collected in this study are available via figshare at the following: the CRISPRa screen data at 10.6084/m9.figshare.31128976 (ref. ^79^), Perturb-seq data at 10.6084/m9.figshare.31119196 (ref. ^80^), and in situ Perturb-seq data at 10.6084/m9.figshare.31119634 (ref. ^81^) and 10.6084/m9.figshare.31119607 (ref. ^82^). Gene expression data from The Cancer Genome Atlas were obtained from the International Cancer Genome Consortium repository (https://docs.icgc-argo.org/)^35^. Previously published CRISPRa Perturb-seq data were downloaded from the Gene Expression Omnibus https://www.ncbi.nlm.nih.gov/geo/ (accession no. GSE133344)^39^. Source data are provided with this paper.

## Code availability

Code to reproduce results and figures presented in this study is available via GitHub at https://github.com/Jerby-Lab/High_content_CRISPR_immunomodulators under BSD 3-Clause License (10.6084/m9.figshare.31119718)^83^.
