# Supplementary material for: High-content CRISPR activation screens identify synthetically lethal RNA-based mechanisms to sensitize cancer cells to targeted T cell cytotoxicity
Source: Nat Genet. 2026 Apr 7;58(4):841–53. doi: 10.1038/s41588-026-02561-7 (PMC13083246; doi:10.1038/s41588-026-02561-7)
Supplement: Supplementary file 1 — Supplementary Information [file 41588_2026_2561_MOESM1_ESM.pdf]

nature genetics

Supplementary information https://doi.org/10.1038/s41588-026-02561-7

High-content CRISPR activation screens identify synthetically lethal RNA-based mechanisms to sensitize cancer cells to targeted T cell cytotoxicity

In the format provided by the authors and unedited

Supplementary Information

Supplementary Figures

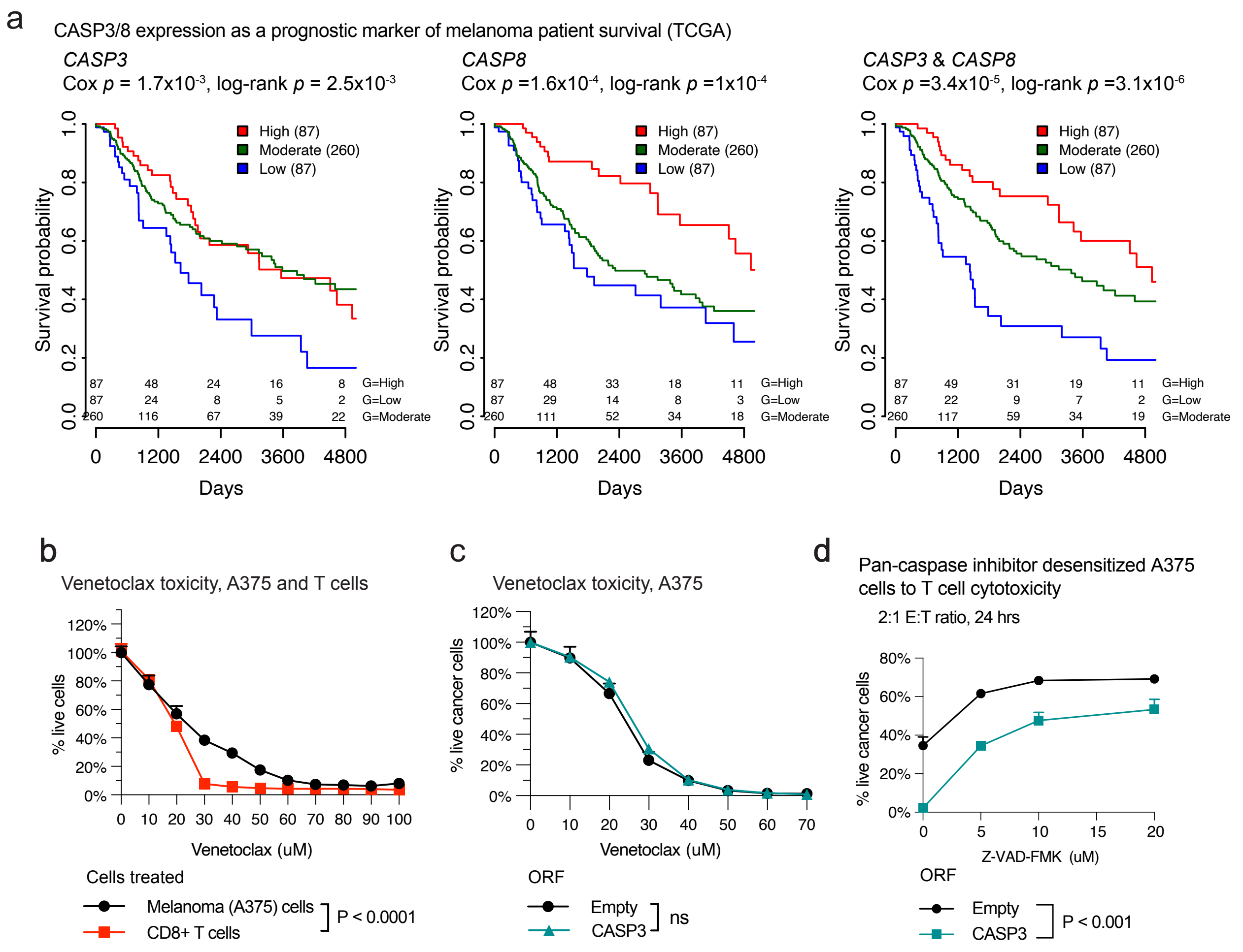

Supplementary Figure 1. The distinct effects of caspases overexpression and Bcl2 protein inhibition. (a) Kaplan–Meier overall survival curves of melanoma patients stratified by expression levels of *CASP3*, *CASP8*, and average expression of both *CASP3* and *CASP8* (high and low defined as top and bottom 20 percentiles, and moderate otherwise). The bottom rows show the number of individuals at risk in each group. Cox *p*-values were calculated from the Wald statistic of covariate-controlled Cox proportional hazards regression models applied to the expression (log1p-transformed tpm) values. The log-rank *p*-value was derived from comparing discretized predictors. **(b)** Viability (y axis) of A375 and primary CD8 T cells measured via PrestoBlue following 24 hours of treatment with the Bcl2 inhibitor Venetoclax at varying doses (x axis) for 24 hours in monoculture. **(c)** Viability (y axis) of control and $CASP3^{OE}$ A375 cells measured via PrestoBlue following 24 hours of treatment with Venetoclax at varying doses (x axis). **(d)** TCR-specific cytotoxicity of control and $CASP3^{OE}$ A375 cells in coculture with NY-ESO-1 TCR T cells (2:1 E:T, 24 hours) treated with pan-caspase inhibitor (Z-VAD-FMK) at varying doses (x axis) during coculture. (b-d) Data are represented as mean ± SD. P-values were calculated via Satterthwaite's ANOVA in a dose-controlled two-sided linear mixed-effects model (LMM).

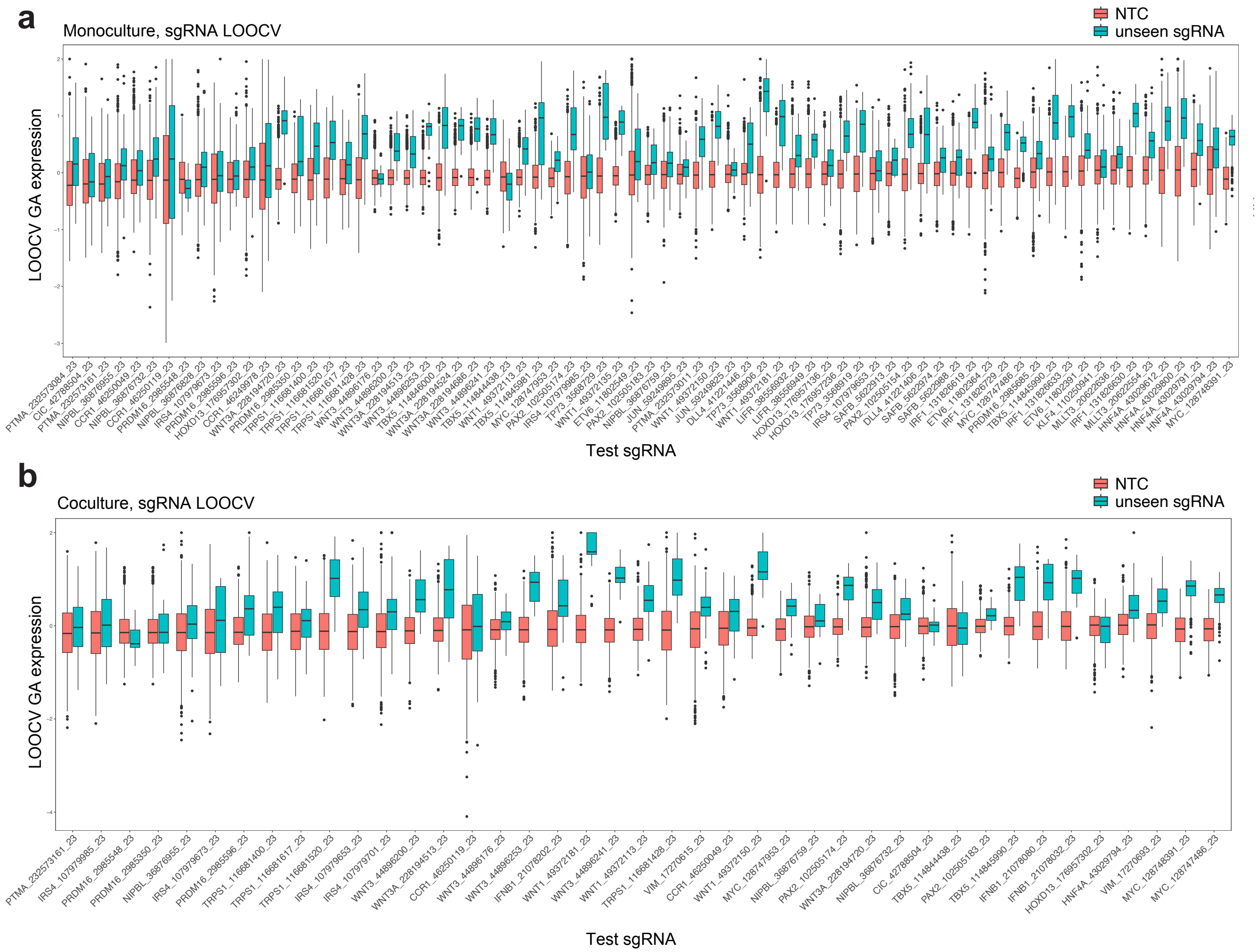

Supplementary Figure 2. sgRNAs targeting the same genes show consistent effects on the cell transcriptome. The GA signatures derived in the leave-one-sgRNA-out cross-validation procedure were tested in the pertaining left-out sgRNA (x axis) for overexpression (y axis) in the control (orange) versus sgRNA (turquoise) cells in monoculture **(a)** and coculture **(b)**. Boxplots middle line: median; box edges: 25th and 75th percentiles; whiskers: most extreme points that do not exceed ± IQR x 1.5; further outliers are marked individually with circles (minima/maxima). Statistical significance based on one-tailed t-test p-values are provided in **Supplementary Table 6**.

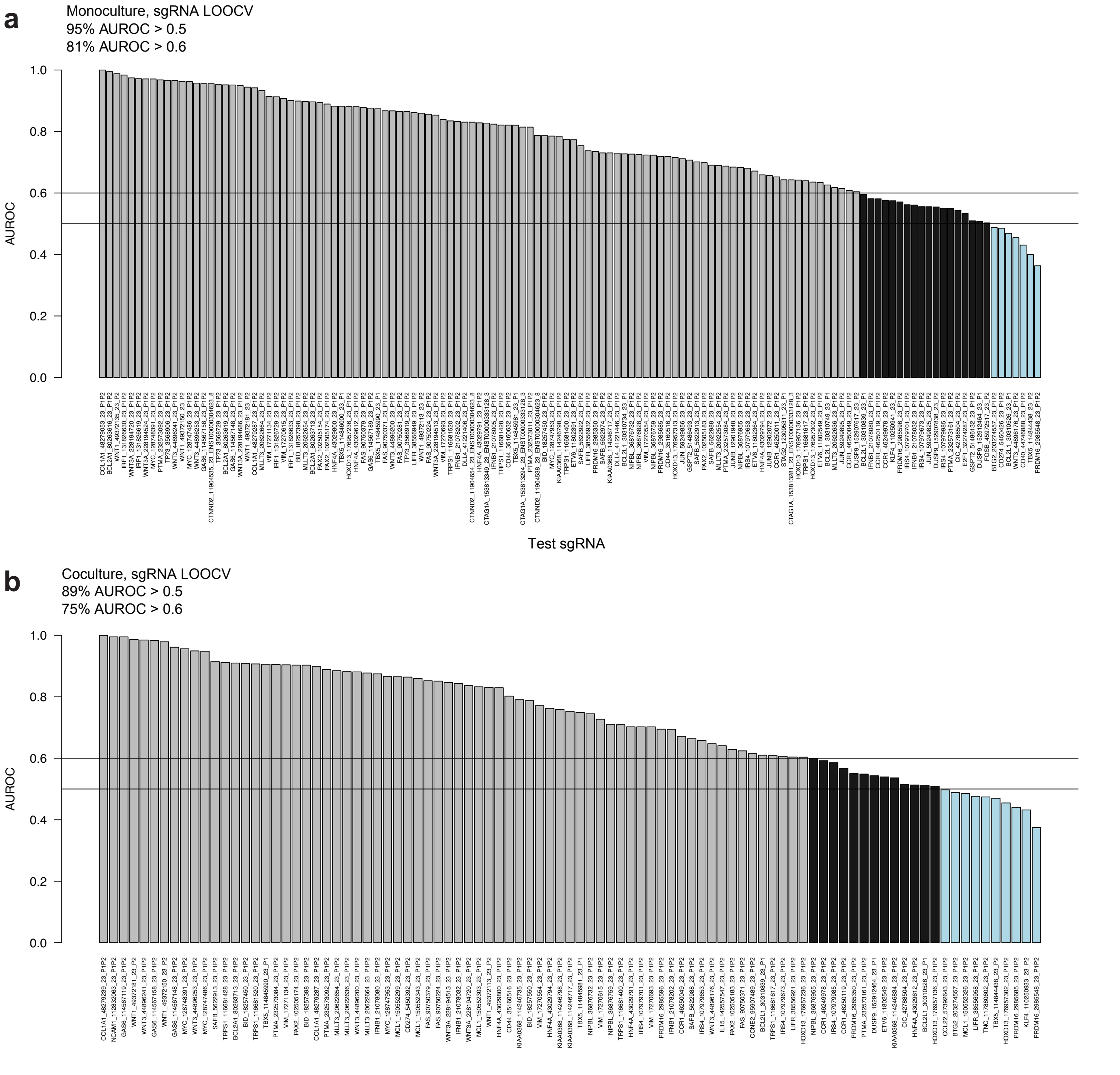

Supplementary Figure 3. GA signatures are predictive of perturbation with unseen data and sgRNAs. The AUROCs (y axis) obtained for each GA signature derived in the leave-one-sgRNA-out cross-validation procedure when tested in the pertaining left-out sgRNA (x axis) for its predictive value in distinguishing between control and sgRNA cells in monoculture **(a)** and coculture **(b)**. Grey, black, and cyan bars depict AUROC values > 0.6, between 0.5 and 0.6, and below 0.5, respectively.

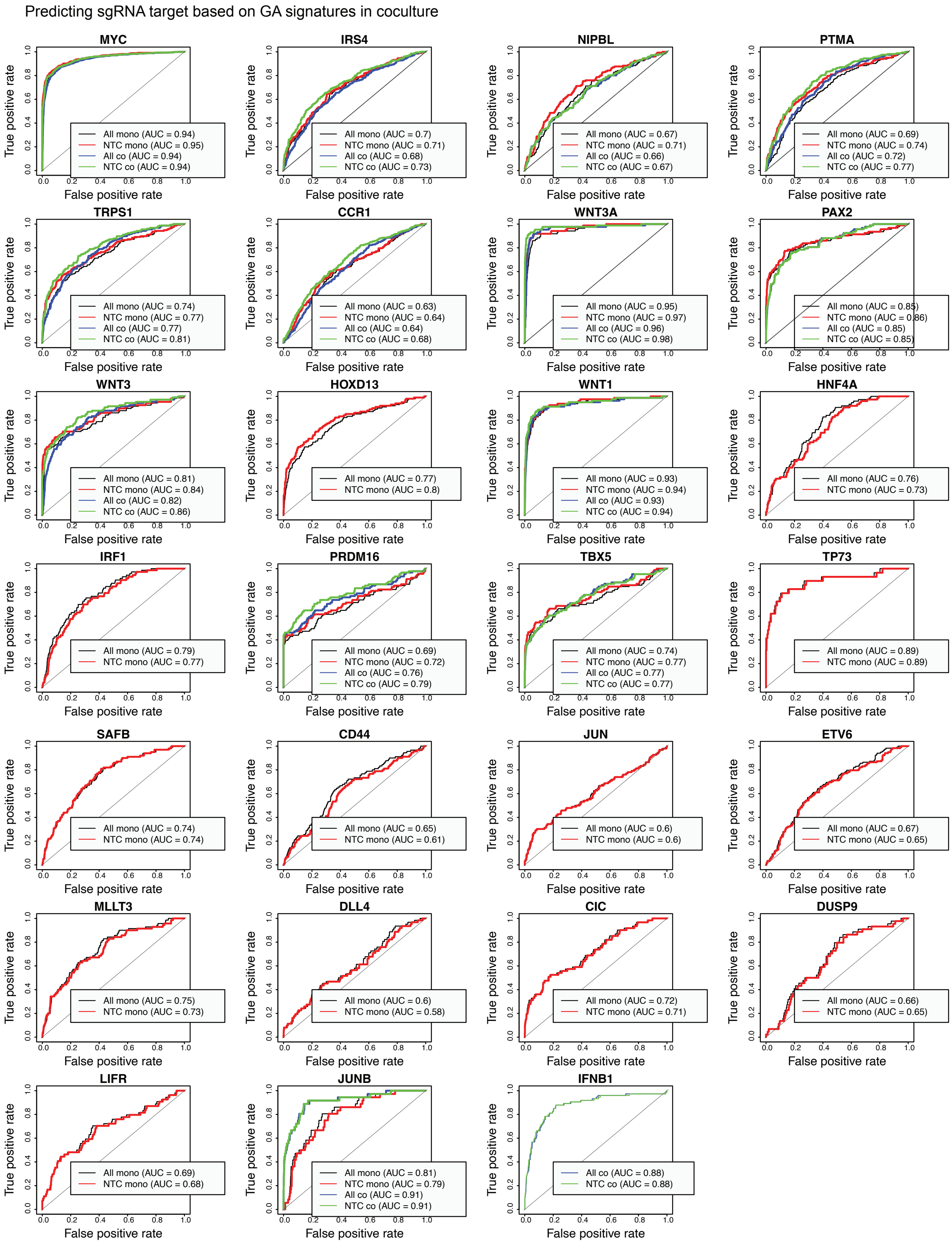

Supplementary Figure 4. Perturb-seq monoculture GA signatures validation in coculture. GA signatures derived separately from monoculture (black and red) or coculture (blue and green) were applied to classify cells as subject to the pertaining gene activation, when considering all cells in the Perturb-seq coculture datasets, or only control (NTC) and cells with the pertaining gene activation based on sgRNA detection. Only GA signatures with at least 5 genes were used as predictors. Mono (monoculture), co (coculture).

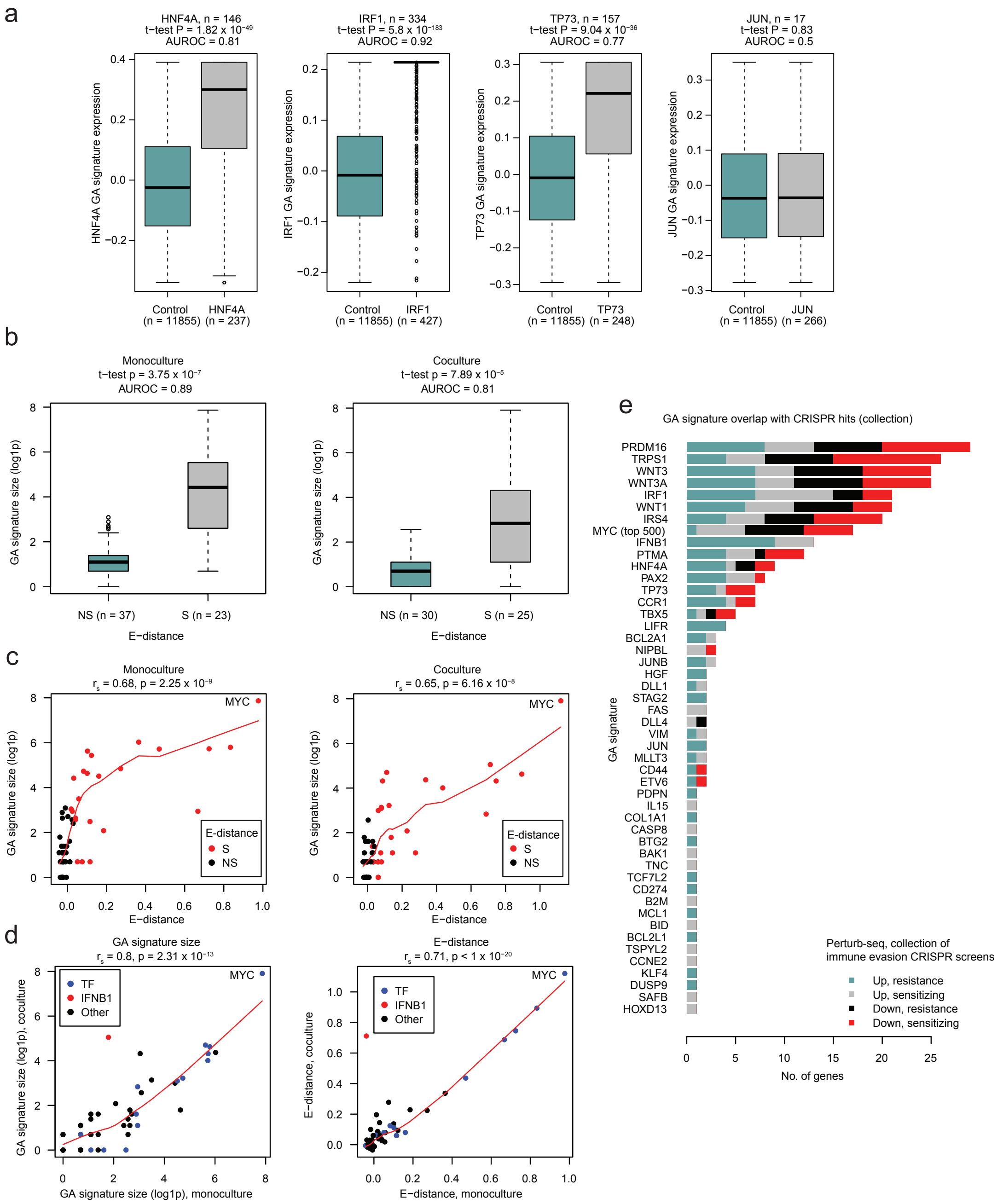

Supplementary Figure 5. Validation of GA signatures. (a) Testing the GA signatures in an external Perturb-seq CRISPRa data of K562 cells[1]: Expression of 4 GA signatures in control cells and cells carrying a CRISPRa sgRNA targeting the pertaining gene, shown for *HNF4A, IRF1, TP73*, and *JUN*. **(b)** The size of the GA signature (y axis) stratified to perturbations with significantly (S) large E-distances ($p < 0.05$, empirical permutation tests) or not significant (NS), when using the monoculture (left) or coculture (right) Perturb-seq data to identify the GA signatures and compute the E-distance (**Methods, Supplementary Note 1**). **(c)** GA signature size (x axis) as a function of E-distance (x axis) in monoculture (left) and coculture (right) Perturb-seq data. Significantly large E-distances ($p < 0.05$, empirical permutation tests) are marked in red. **(d)** Left: GA-signature size when identifying the signature only based on monoculture (x axis) or coculture (y axis) Perturb-seq data; Right: Perturbation E-distance in monoculture (x axis) or coculture (y axis) Perturb-seq data. TFs and *IFNB1* are marked in blue and red, respectively. Other perturbations are in black. **(e)** The number of sensitizing and resistance genes found in each of the 61 GA signatures, shown for both up- and down-regulated subsets of each signature. In contrast to **Fig. 3e**, here gene annotation is based on this study and a collection of immune evasion CRISPR screens[2–8]. (a-b), boxplots middle line: median; box edges: 25th and 75th percentiles; whiskers: most extreme points that do not exceed ± IQR x 1.5; further outliers are marked individually with circles (minima/maxima).

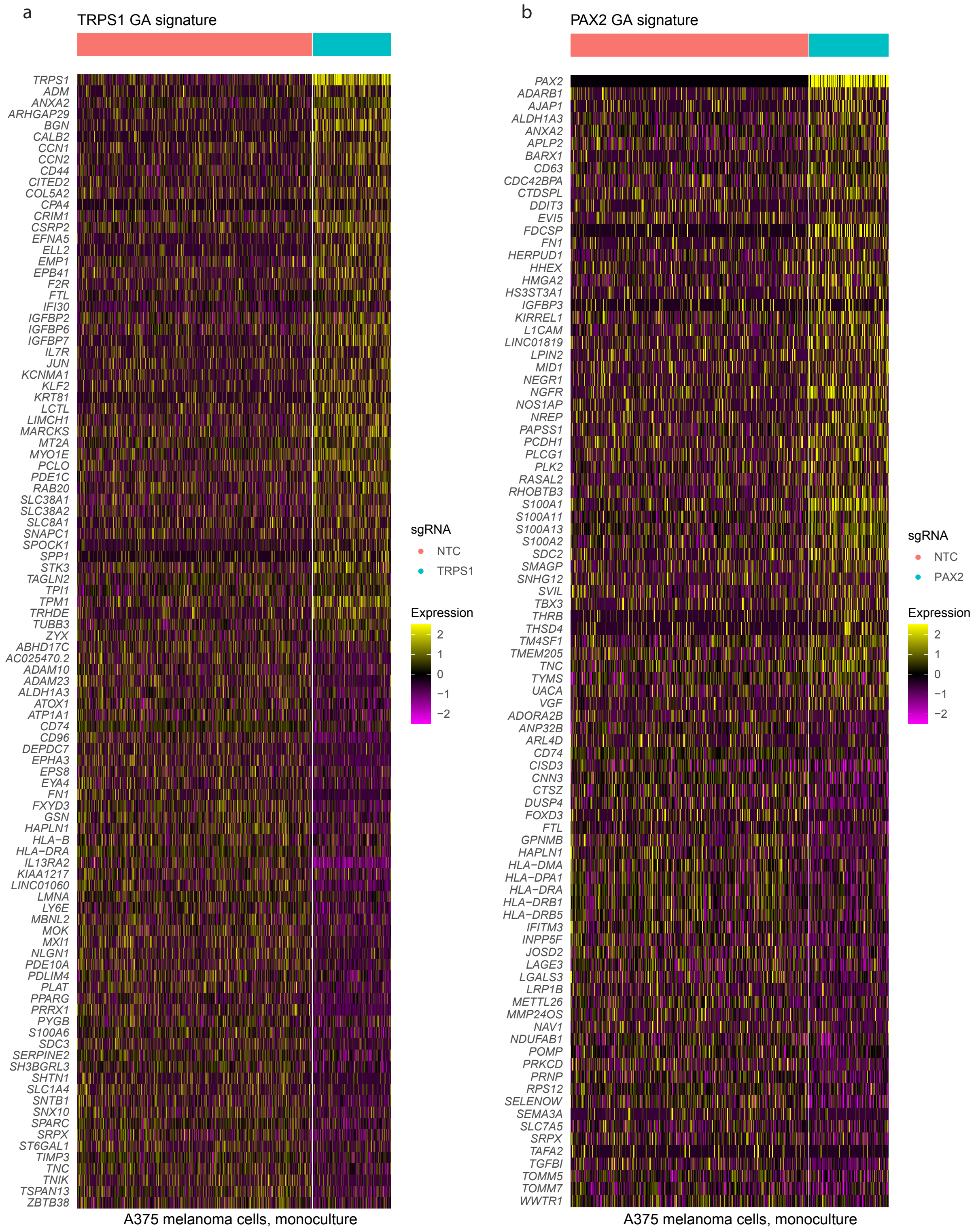

Supplementary Figure 6. *TRPS1* and *PAX2* GA signatures. Normalized expression values (centered and scaled log1p-transformed tp10k) of **(a)** *TRPS1* GA signature genes across the control and *TRPS1* CRISPRa cells (columns and topmost color bar) in monoculture, and **(b)** *PAX2* GA signature genes across the control and *PAX2* CRISPRa cells (columns and topmost color bar) in monoculture.

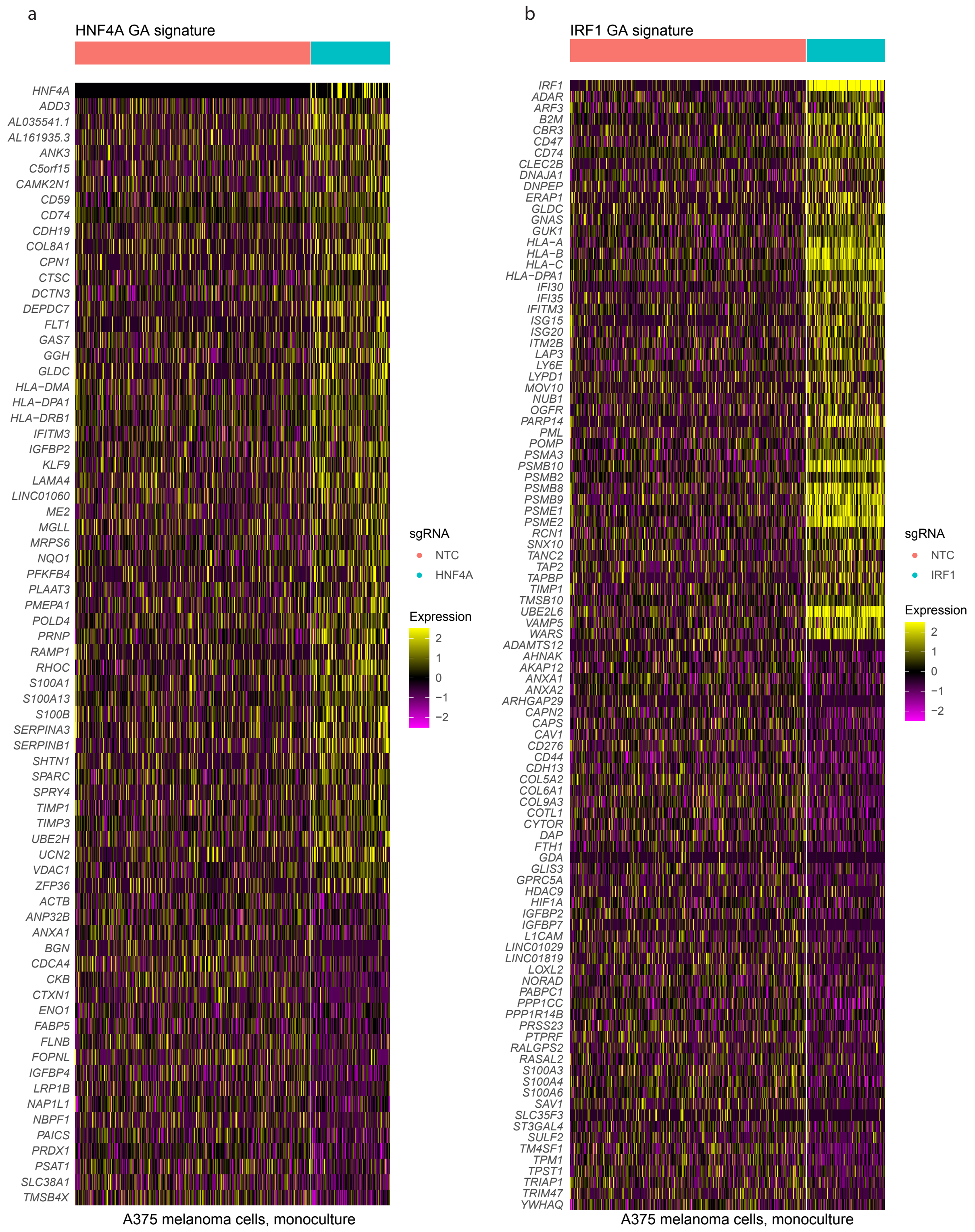

Supplementary Figure 7. *HNF4A* and *IRF1* GA signatures. Normalized expression values (centered and scaled log1p-transformed tp10k) of **(a)** *HNF4A* GA signature genes across the control and *HNF4A* CRISPRa cells (columns and topmost color bar) in monoculture, and **(b)** *IRF1* GA signature genes across the control and *IRF1* CRISPRa cells (columns and topmost color bar) in monoculture.

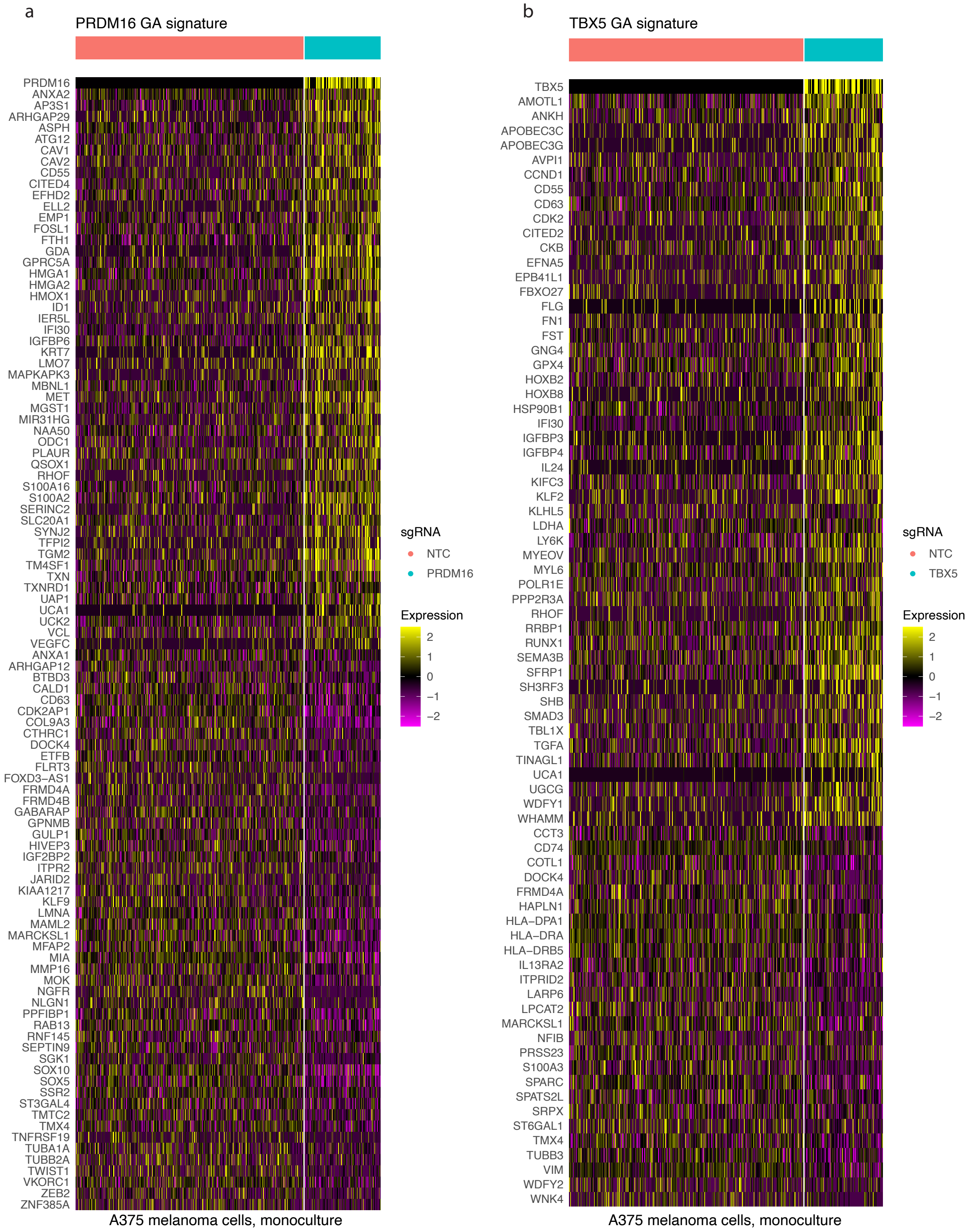

Supplementary Figure 8. *PRDM16* and *TBX5* GA signatures. Normalized expression values (centered and scaled log1p-transformed tp10k) of **(a)** *PRDM16* GA signature genes across the control and *PRDM16* CRISPRa cells (columns and topmost color bar) in monoculture, and **(b)** *TBX5* GA signature genes across the control and *TBX5* CRISPRa cells (columns and topmost color bar) in monoculture.

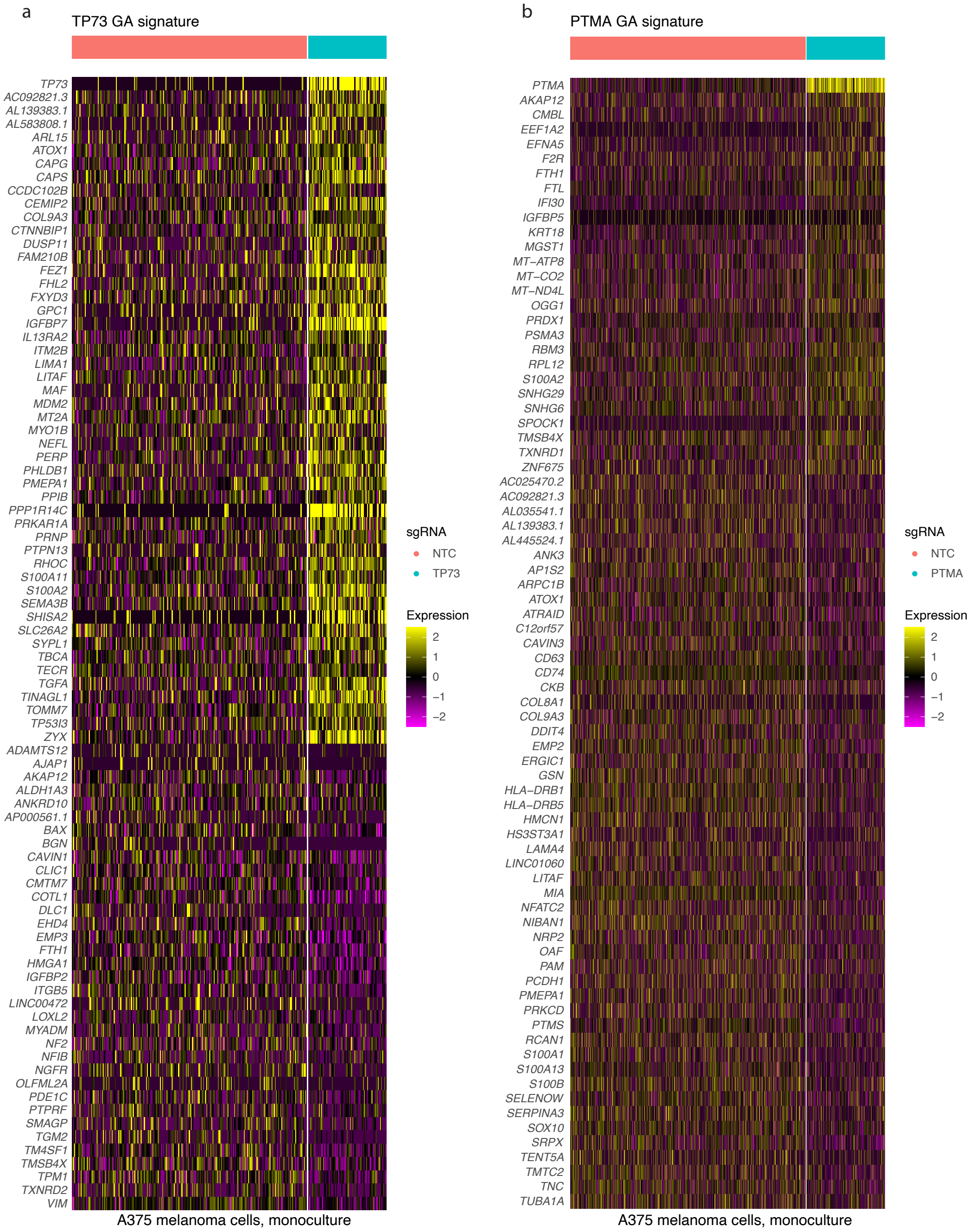

Supplementary Figure 9. *TP73* and *PTMA* GA signatures. Normalized expression values (centered and scaled log1p-transformed tp10k) of **(a)** *TP73* GA signature genes across the control and TP73 CRISPRa cells (columns and topmost color bar) in monoculture, and **(b)** *PTMA* GA signature genes across the control and PTMA CRISPRa cells (columns and topmost color bar) in monoculture.

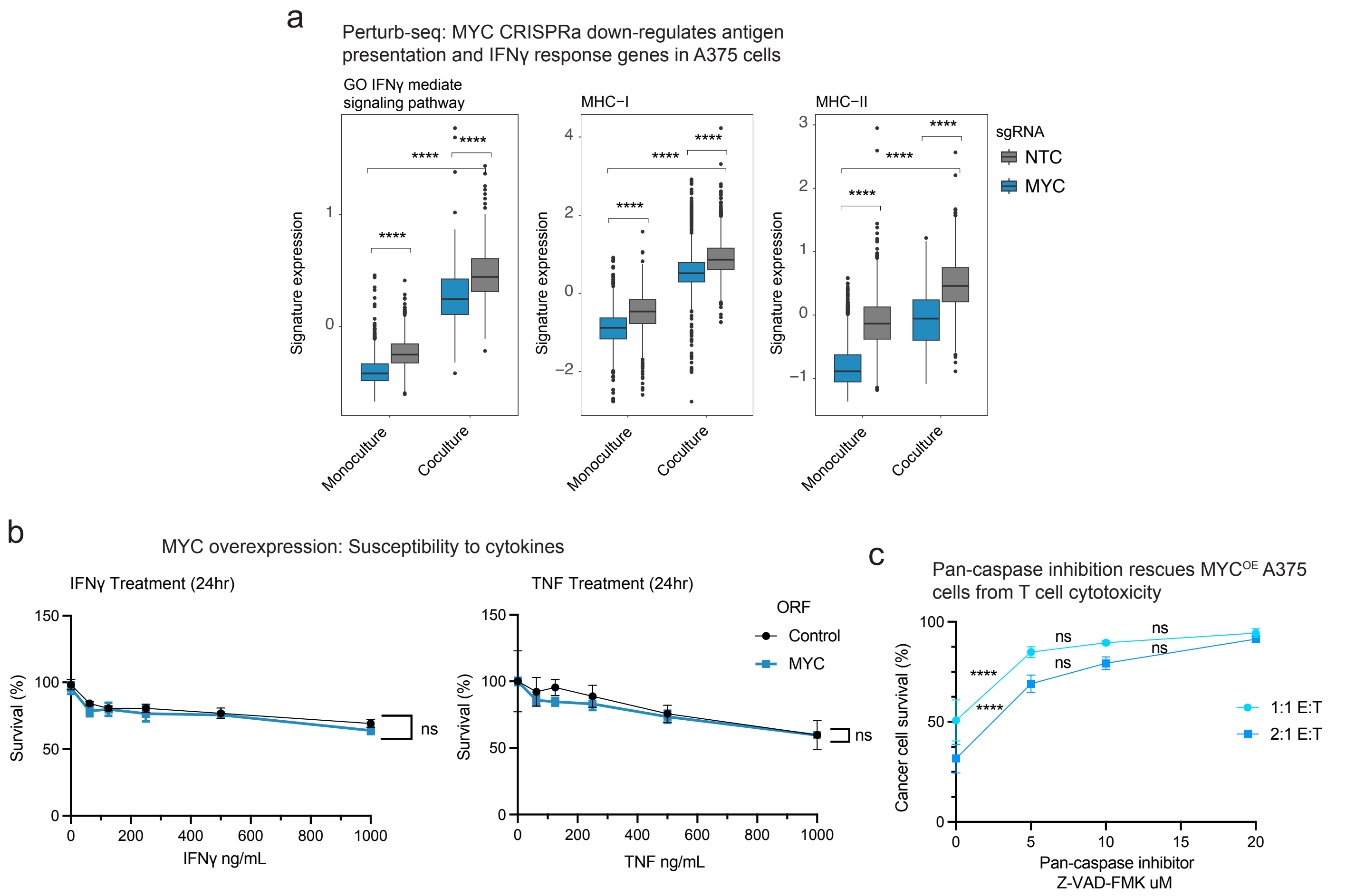

Supplementary Figure 10. Further investigation of MYC regulation of cancer cell immunity and susceptibility. (a) Expression of GO IFNγ-mediated signaling pathway, MHC-I, and MHC-II genes in control (grey) and *MYC* CRISPRa (blue) A375 cells in monoculture and coculture Perturb-seq data. $^{****}P < 0.0001$, t-test. Boxplots middle line: median; box edges: 25th and 75th percentiles; whiskers: most extreme points that do not exceed ± IQR x 1.5; further outliers are marked individually with circles (minima/maxima). **(b)** Viability of MYC^{OE} ORF A375 cells following 24-hour treatment with IFNγ or TNF at various concentrations (x axis). Data are presented as the mean ± SD. Statistical significance was evaluated using a two-way ANOVA, $n = 6$ technical replicates per ORF and timepoint. **(c)** MYC^{OE} A375 cell survival (y axis) in 24-hour coculture with NY-ESO-1 TCR T cells at 2:1 or 1:1 E:T and pan-caspase inhibitor (Z-VAD-FMK) treatment at varying doses (x axis) during coculture. Statistical significance was evaluated using an ordinary two-way ANOVA comparing between sequential doses for each E:T. $^{****}P < 0.0001$, $n = 3$ technical replicates per E:T and dose.

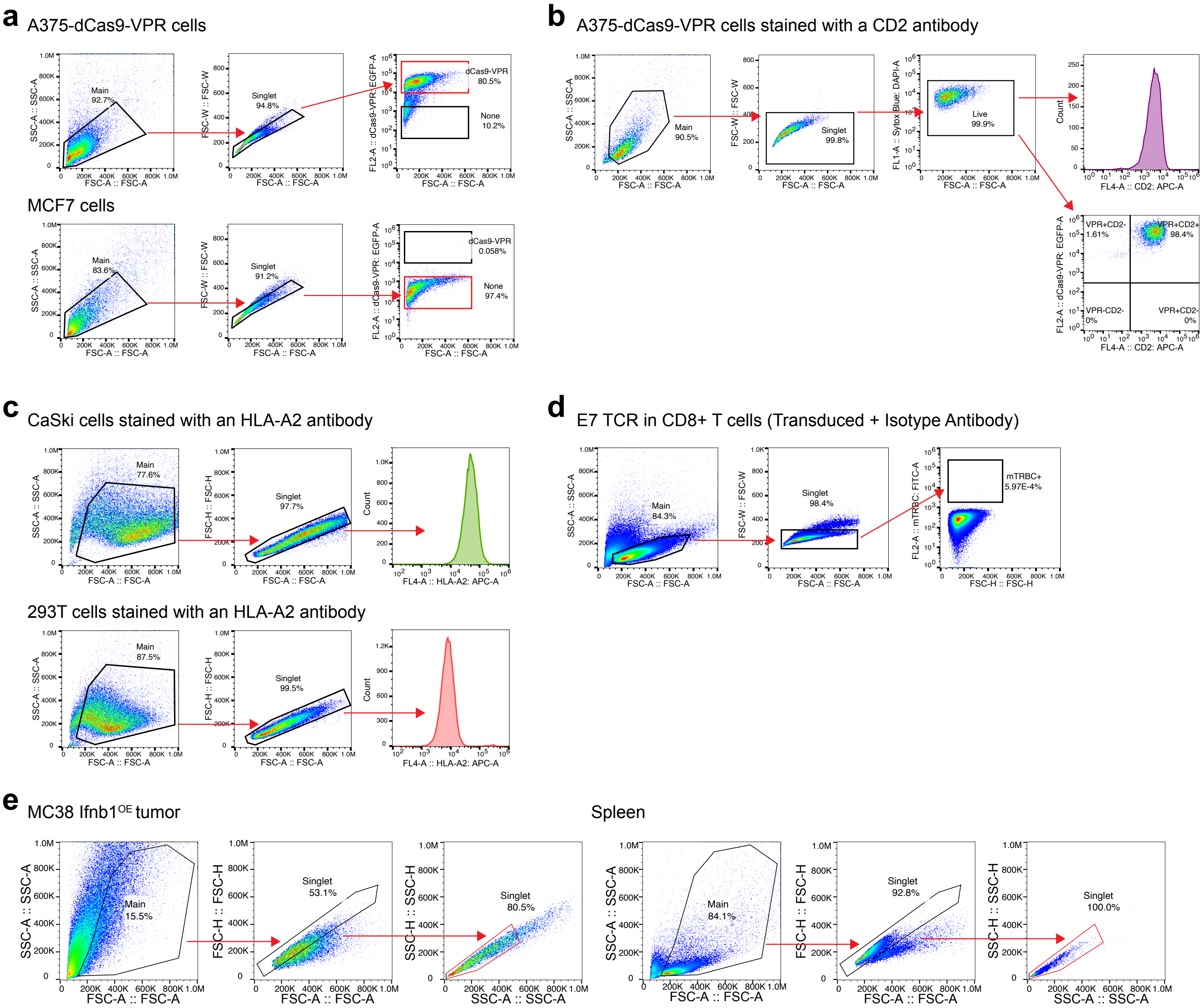

Supplementary Figure 11. Flow cytometry gating strategies. Gating strategies for **(a)** A375 dCas9-VPR (top) and MCF7 (bottom) cells, showing sequential gating prior to assessment of NY-ESO-1 TCR expression, corresponding to **Extended Data Fig. 1e**. **(b)** Selected A375 dCas9-VPR clone used to assess CD2 activation following sgRNA delivery, with final CD2 gates in histogram (top) or pseudocolor (bottom) plots, corresponding to **Extended Data Fig. 1h,i**, respectively. **(c)** HLA-A2 expression in CaSki (top) and Lenti-X^{TM} HEK293T (bottom) cells stained with anti-HLA-A2 antibody, corresponding to **Extended Data Fig. 3b**. **(d)** Transduced E7 TCR CD8 T cells stained with isotype control antibody, showing the gating used to define the E7 TCR-positive population that was sorted in **Extended Data Fig 3d**. **(e)** *Ifnb1* overexpressing MC38 tumors and spleens harvested from mice, shown immediately prior to DAPI gating, with final DAPI gates corresponding to **Extended Data Fig 10f**.

Supplementary Tables

Provided as a separate excel file.

Supplementary Table 1. CRISPRa sgRNA library used in the ex vivo screen.

Supplementary Table 2. Hits identified in the CRISPRa screens and matching summary statistics.

Supplementary Table 3. Perturb-seq and in vivo CRISPRa sgRNA library.

Supplementary Table 4. Results of single-cell differential gene expression analysis (edgeR) in control (NTC) A375 cells in co-culture with NY-ESO-1 TCR T cells versus monoculture.

Supplementary Table 5. Perturb-seq screen on-target CRISPRa overexpression. Differential gene expression analysis (edgeR) for on-target CRISPRa overexpression in the Perturb-seq data was conducted per sgRNA in monoculture **(a)** and coculture **(b),** and per target gene (across all its sgRNAs) in monoculture **(c)** and coculture **(d)**. See also **Extended Data Figs. 4-5**.

Supplementary Table 6. Perturb-seq screen consistency across sgRNAs. Leave one sgRNA cross-validation results conducted to examine the consistency between the transcriptional shifts observed with different sgRNAs targeting the same gene in monoculture **(a)** and coculture **(b)**.

Supplementary Table 7. GA signatures identified in the Perturb-seq screen: **(a)** GA signatures, **(b)** GA signature cluster analysis results (see also **Extended Data Fig. 7b**).

Supplementary Table 8. Hits identified in the in vivo CRISPRa screen and matching summary statistics.

Supplementary Table 9. GA signatures identified in the in situ Perturb-seq screen conducted in A375 xenografts in NSG mice.

Supplementary Table 10. In situ Perturb-seq screens in B16 engrafted in C57BL/6 mice: **(a)** GA signatures, **(b)** *Lifr* ligand-specific GA signatures, and **(c)** pMCPs.

Supplementary Table 11. Oligonucleotide and antibody information.

Supplementary Note 1

Supplemental Methods

Plasmids

To validate and investigate specific genes, sgRNAs were cloned into pMCB306[9] (GFP), pMCB307[10] (BFP), or pMCB320[9] (mCherry) at the BstXI and BlpI restriction sites. The pLV-EF1a-IRES-Puro (Addgene, #85132) construct was used for generating ORFs. The resulting plasmids were transformed into DH5a competent E. Coli (Zymo), then extracted using the GeneJET Plasmid Miniprep Kit. Plasmid sequences were confirmed through Sanger sequencing (MCLAB) or whole-plasmid sequencing (Plasmidsaurus).

Virus production

General virus production was conducted with Lenti-X™ HEK293T cells and the TransIT-Lenti (MirusBio, MIR 6604) transfection agent following the TransIT® Lentivirus System protocol. Lentivirus and retrovirus production for T cell transduction was performed as follows: one day before transfection, 12e6 Lenti-X™ 293T cells were seeded in a T-75 flask and cultured in 15 mL of Opti-MEM I Reduced Serum Medium (Gibco 31985088) supplemented with 1x GlutaMAX Supplement (ThermoFisher, Cat #35050061), 1mM Sodium Pyruvate, 5% FBS, and 5 mM MEM Non-Essential Amino Acids. For lentivirus, on the day of transfection, 2.8 mL of Opti-MEM was mixed with 10 ug psPAX2 (Addgene, #12260), 4.33 ug pMD2.G (Addgene, #12259), and 14 ug of transfer plasmid and mixed briefly. For retrovirus, 2.8 mL of Opti-MEM was mixed with 12.5 ug pUMVC (Addgene, #8449), 1.5 ug pCMV-VSV-G (Addgene, #8454), and 14 ug of transfer plasmid and mixed briefly. Next, 85 uL of TransIT-Lenti was added, mixed briefly, then incubated for 10 min at Room Temperature (RT). After removing 6.67 mL of media from the 293T cells, the transfection mixture was added dropwise. Six hours after transfection, the media was replaced with 15 mL of fresh media. Virus was harvested 24 hours and 48 hours after transfection, then combined and concentrated 100X using Lenti-X™ Concentrator (Takara Bio, 631232) following the manufacturer's protocol. Virus was either used immediately or aliquoted and frozen at -80°C for later use.

Lentivirus titration

To titrate lentivirus, 2e6 cells were seeded in 6 wells of a 12-well plate in growth media plus 8ug/mL polybrene. Immediately after, 0 uL, 2.5 uL, 5 uL, 10 uL, 20 uL, or 40 uL of lentivirus was added to the wells and mixed thoroughly. Twenty-four hours after transduction, cells from each condition were trypsinized and seeded into a 96-well plate at a concentration of 5e4 cells/well in 2 wells with antibiotic selection and in 2 wells without antibiotic selection. When the no-virus condition with antibiotic selection contained no viable cells, cell viability was measured with PrestoBlue (Invitrogen, Cat# A13261) following the manufacturer's protocol. The titer was calculated using the formula: Titer (TU/uL) = (N x P x D)/V, where *N* denotes the number of cells in each well used for infection, *P* denotes the proportion of live cells compared to the no-antibiotic condition, *D* denotes the dilution fold of the virus, *V* denotes virus volume used for infection in each well, in uL, and *TU* denotes the transduction unit.

Flow cytometry

Flow cytometry was performed on a Sony SH800, BD FACSAria Fusion, BD FACSAria II, or BD Influx flow cytometer. Briefly, for all flow cytometry procedures, cells were washed and resuspended in FACS staining buffer (PBS + 2 % FBS) and incubated with Fc receptor-blocking reagents (human or mouse, as appropriate) for 5 min at RT prior to antibody staining at indicated dilutions for 15-20 min on ice, unless specified otherwise, in the dark.

All antibodies and corresponding dilutions are listed in **Supplementary Table 11**. Cells were washed and stained with DAPI (Thermo Fisher Scientific, 62248, 1:10,000) according to the corresponding gating strategies shown in **Supplementary Fig. 11**.

To assess the purity of $CD8^+$ T cell populations, cells were labeled with fluorescent-conjugated anti-CD3, anti-CD4, and anti-CD8 antibodies. Two methods were employed to confirm the expression of the NY-ESO-1 TCR on the T cell surface. First, $CD8^+$ T cells were labeled with primary anti-HA and anti-PC antibodies at a 1:50 dilution for 1 hour at RT, followed by anti-mouse AF-488 (Cell Signaling Technology, 4408S) and anti-rabbit AF-647 (Cell Signaling Technology, 4414S) secondary antibodies at a 1:500 dilution for 30 minutes at RT in the dark, respectively. This labels the HA and PC tags that are present on the NY-ESO-1 TCR (**Extended Data Fig. 1c**). Second, in an independent experiment, T cells were labeled with an APC-labeled tetramer that binds to a TCR that specifically recognizes human HLA-A*02:01 conjugated with the NY-ESO-1 antigen SLLMWITQC (NIH Tetramer Facility, **Extended Data Fig. 1d**). E7 TCR T cells were sorted with the anti-mouse TCR β chain antibody (BioLegend, 109215, **Extended Data Fig. 3d**).

For MC38 tissues collected in vivo, tissues were excised, mechanically dissociated, and passed through a 100 µm cell strainer. Red blood cells were lysed using Red Blood Cell Lysing Buffer (Sigma, R7757) for 15 min at 4 °C.

Cells were pelleted, washed, passed through a 70 µm strainer, and resuspended in FACS staining buffer to generate single-cell suspensions. Cells were then incubated with mouse Fc receptor-blocking reagent (BioLegend, 101302, 1:50) and then stained with DAPI (Thermo Fisher Scientific, 62248, 1:10,000) to distinguish live ($DAPI^-$) from dead ($DAPI^+$) populations (**Extended Data Fig. 10f**).

In vitro proliferation assay

A375 and CaSki cells were seeded in 24-well plates and monitored for 16 hours via Incucyte. Phase-contrast images of cells were acquired every 1.5 hours, and cell confluence was calculated using the integrated software. Relative confluence was calculated by dividing confluence at each time point by the initial confluence.

Western blot

Total protein lysate was extracted from cells using Pierce RIPA buffer (Thermo Fisher Scientific, 89900) supplemented with Halt Protease Inhibitor Cocktail (Thermo Fisher Scientific, 87785). Protein concentration was measured using the Qubit Protein Assay Kit (Thermo Fisher Scientific, Q33211). 20 ug of lysate was mixed with Laemmli buffer, boiled at 95°C for 5 min, then either stored at -80°C for future use or immediately used in gel electrophoresis. Samples were run through an SDS-PAGE gel and then wet transferred to a PVDF or nitrocellulose membrane. Membranes were blocked with EveryBlot Blocking Buffer (Bio-Rad, 12010020) at RT for 5 min, and then incubated with primary antibody [alpha-tubulin (Cell Signaling Technology 2144S, 1:1000), NY-ESO-1 (Santa Cruz Biotechnology sc-53869, 1:500), Caspase-3 (Cell Signaling Technology 9662S, 1:1000), HPV16 E7 (Santa Cruz Biotechnology sc-65711, 1:50), c-MYC (BioLegend 626801, 1:1,000), or β-actin (Cell Signaling Technology 4970S, 1:5000)] in EveryBlot Blocking Bufferat 4°C overnight. The following day, membranes were washed and incubated with a secondary antibody [anti-rabbit IgG, HRP-linked (Cell Signaling Technology 7074S, 1:1000), anti-mouse IgG, HRP-linked (Cell Signaling Technology 7076S, 1:1000)] in EveryBlot Blocking Buffer at RT for 1 hour. The bands were detected by incubating the membrane with Clarity Western ECL Substrate (Bio-Rad 1705060) and exposing it to a ChemiDoc System (Bio-Rad).

All antibodies used and corresponding dilutions are listed in **Supplementary Table 11**.

Quantitative PCR

Total RNA was extracted using the Zymo Quick-DNA/RNA Miniprep Plus Kit (D7003). RNA was then converted into cDNA (Qiagen, 205311) before use in qPCR. qPCR was performed using Applied Biosystems PowerUp SYBR Green Master Mix (ThermoScientific, A25742) on a Bio-Rad CFX96 Touch Real-Time PCR Detection System. Relative RNA expression was determined by normalizing to *GAPDH* expression.

Primer used for qPCR and other purposes are listed in **Supplementary Table 11**.

Stably overexpressing hits in human cancer cell lines and T cells

Syngeneic A375 and CaSki cell lines with stable overexpression of top hits were generated as follows. sgRNA sequences were ordered as oligos and cloned into the pMCB320 plasmid using the BstXI and BlpI restriction sites. To generate the ORF plasmids, gene fragments were ordered from Twist Biosciences and cloned into the pLV-EF1a-IRES-Puro backbone using NEBuilder HiFi DNA assembly. Lentivirus was generated as described above. Cells were transduced with lentivirus at the indicated MOI for 24 hours. Three days after transduction, cells were selected using the appropriate selection antibiotic for three days or until control cells died. To examine the impact of *CASP3* overexpression on T cells, CASP3 and control ORFs were used to transduce primary CD8 T cells in the same manner as described above, but without drug selection, using qPCR to confirm ORF-based *CASP3* overexpression (**Fig. 2d**, left).

Cytokine and drug treatments

Cells were treated with Fc:FasL (AdipoGen AG-40B-0132-C010), IFNγ (PeproTech 300-02), TNF (PeproTech 300-01A), Z-VAD(OMe)-FMK (SantaCruz Biotechnology sc-311561), Venetoclax (Selleck Chemicals S8048), Etoposide (Selleck Chemicals S1225), or recombinant human Wnt3a protein (R&D Systems, 5036-WN-010/CF) at the indicated concentrations. In the Wnt3a experiments, A375 and NY-ESO-1 TCR T cells were treated before coculture, and cancer cell viability was measured with PrestoBlue after 24 hours of coculture. For the drug-only experiments, cells were cultured with the drug for 24 hours, then viability was measured with PrestoBlue. In the CD8 T cell chronic activation experiment, cells were treated every 3 days with Wnt3a. Supernatant was harvested at the indicated timepoints and assessed with human IFNγ ELISA (Biolegend, 430104) following the manufacturer's protocol.

Patient survival analyses

Gene expression and matching overall survival data of 434 TCGA[11] (The Cancer Genome Atlas) melanoma samples were downloaded from the ICGC (International Cancer Genome Consortium, https://docs.icgc-argo.org/). The log1p-transformed tpm values were used to quantify the prognostic value of each gene in predicting overall survival using a Cox proportional hazards regression model.

Computing signature expression

The expression of a gene signature was computed with additional normalizations to filter technical variation, as described below, following the procedure previously described in Yeh et al.[12]. Given a gene signature and a gene expression matrix *E* ($\log_2$1p-transformed tp100k), genes are binned into *G*/*N* expression bins according to their average expression across the cells ($\log_2$1p-transformed of average tp100k), wherein *G* is the number of unique genes quantified in the gene expression matrix. $N = 20$ was used for spatial data, and $N = 100$ was used for scRNA-Seq data.

The expression of signature *S* in cell *j* is then defined as:

$$\mathrm{SE_j} = \frac{\sum_{\mathrm{i}\in\mathrm{S}} \mathrm{E_{ij}^c}}{\mathbb{E}_{\tilde{\mathrm{S}}}\left[\sum_{\mathrm{i}\in\tilde{\mathrm{S}}} E_{\mathrm{ij}}^{c}\right]}$$

Where $\mathrm{E_{ij}^c}$ is the centered and scaled expression of gene *i* in cell *j*, defined as $\mathrm{E_{ij}^c} = \frac{(\mathrm{E_{ij}} - \mathbb{E}[\mathrm{E_i}])}{\sigma(E_i)}$, and $\tilde{\mathrm{S}}$ is a random S-compatible signature. Given a gene signature *S* that consists of *K* genes, with k_b genes in bin *b*, a random signature is *S-compatible* with signature *S* if it consists of overall *K* genes, such that in each bin *b* it has exactly k_b genes. The expected expression of a random S-compatible signature can be computed based on the centered expression data:

$$\mathbb{E}_{\tilde{S}}\left[\sum_{i\in\tilde{S}} E_{ij}^{c}\right] = \sum_{i\in\tilde{S}} \mathbb{E}\left[E_{ij}^{c}\right] = \sum_{b\in B} k_b * A_{bj}$$

Where A_{bj} denotes the average centered expression of genes from bin b in cell j.

The expression of a signature that includes both up (S_{up}) and down (S_{down}) regulated genes is computed as:

$$SE_{S,j} = SE_{S_{up},j} - SE_{S_{down},j}$$

The expression of a GA signature is defined as the expression of its upregulated gene subset minus the expression of its downregulated subset. As signature expression values follow a Gaussian distribution, t-tests were used to compare signature expression values across two cell populations.

GA signatures out-of-sample validations

GA signatures were validated based on an out-of-sample procedure where a GA signature identified based on one "train" subset of cells was tested for its generalizability with another "test" unseen out-of-sample subset of cells. The test dataset was used to examine if the GA signature was overexpressed in the cells with the GA compared to the control cells (one-sided t-tests), and a univariate classifier that predicts cells as being subject to the GA perturbation if having a high expression of the pertaining GA signature was tested on the test dataset with an increasing cutoff to generate a Receiver Operating Characteristic (ROC) curve. The Area Under the ROC curve (AUROC) was computed to evaluate the prediction performance.

Magnitude of perturbation-driven transcriptional shift

The magnitude of the perturbation-driven transcriptional shift was measured in two ways, based on the GA signature size and the previously defined Energy distance (E-distance)[13,14].

GA signature size is simply the number of genes that were found to be differentially expressed in cells with the pertaining gene activation (GA) when compared to the control cells via edgeR differential gene expression analyses (**Methods**). To examine the impact of sample size on the GA signature size, edgeR differential gene expression analyses were repeated with 20 subsets of the data. In each iteration, 80 cells were sampled per target gene, and GA signatures were identified per gene based on the subsampled dataset. The size of the GA signatures identified in the subsampled dataset (average size across 20 iterations) was highly correlated with the size of the GA signatures identified based on the full data ($r = 0.88$, $p = 7.94 \times 10^{-37}$, Pearson correlation; $r_s = 0.9$, $p = 8.89 \times 10^{-41}$, Spearman correlation).

The E-distance bias-corrected score[14] of a perturbation in a given condition (monoculture or coculture) was defined as the Euclidean distance between cells with a certain perturbation and control cells, when normalized to the Euclidean distances observed within each population of cells. More formally, let d be the number of genes in the data, $x_1, \ldots, x_N \in \mathbb{R}^d$ be the transcriptional profiles of cells with a specific gene activation, and $y_1, \ldots, y_M \in \mathbb{R}^d$ be the transcriptional profiles of control cells.

The distance between and within each population is defined as

$$\delta_{XY} = \frac{1}{NM}\sum_{i=1}^{N}\sum_{j=1}^{M}\left\|x_i - y_j\right\|$$

$$\sigma_X = \frac{1}{N(N-1)}\sum_{i=1}^{N}\sum_{j=1}^{N}\left\|x_i - x_j\right\|$$

And similarly, for σ_Y. The Energy distance is defined as the delta between the between-population and within-population distances, defined as

$$E(X,Y) = 2\delta_{XY} - \sigma_X - \sigma_Y$$

The within-population distance is bias corrected because when $i = j$, the distance is zero by definition and thus should not be counted, namely, only pairs of cells that mark different cells are considered.

To determine if the E-score is significantly larger than expected, a random set of N cells was selected from the population of non-control cells profiled under the same conditions, and the E-score was computed for this randomly selected set compared to the control cells. This procedure was repeated 20 times to compute an empirical p-value.

The E-distance bias-corrected scores and empirical *p*-values were computed for each of the 61 perturbations in our screen in each condition (monoculture and coculture) using the first 20 PCs, computed as described above.

Testing GA signatures in an external CRISPRa Perturb-seq dataset

CRISPRa Perturb-seq data collected in K562 cells[1] were downloaded from GEO (GSE133344). 8 target genes were activated via CRISPRa both in the K562 screen and the screen conducted here. Four out of the 8 genes had sufficiently large GA signatures (>10 genes): *HNF4A* (GA signature of 147 genes), *IRF1* (335 genes), *TP73* (158 genes), and *JUN* (18 genes). The target gene was removed from the GA signatures to avoid the on-target gene effects from favorably impacting the results. For each GA signature, it was tested if its expression in the K562 cells with the pertaining gene activation was significantly higher than its expression in the control K562 based on a one-sided t-test and AUROC.

Co-regulation modules

Co-regulation scores were defined for each pair of genes based on the number of GA signatures where the two genes were both up or both downregulated. Each gene was then represented based on its co-regulation scores with all other genes. Hierarchical clustering with Euclidean distance measures was performed using these profiles to identify co-regulation modules (**Fig. 3f**).

GA signature clustering

GA signatures were split into up- and down-regulated genes, resulting in two sub-signatures per GA signature. Sub-signatures with fewer than 10 genes were removed. The overlap between every pair of sub-signatures was computed via a one-sided hypergeometric test. The $\log_{10}$-transformed hypergeometric p-values were used to cluster the sub-signatures via hierarchical clustering ("hclust" function, *stats* R package, version 4.2.0, **Extended Data Fig. 7b**).

Statistical measure of spatial clonality

The following procedure was devised to quantify the magnitude and statistical significance of perturbation spatial clonality, that is, the tendency of cells carrying the same perturbation to co-localize. First, for each cancer cell, the fraction of cancer cells carrying the same perturbation in its FOV was computed as the cell co-localization score. The average co-localization score of all the cells carrying the same perturbation was defined as the raw spatial clonality value for that perturbation. To determine whether the raw values were significantly higher than expected by random, the perturbations were shuffled across the cancer cells per tumor, and the values were recomputed. This process was repeated 100 times to generate the null distribution per perturbation. The raw spatial clonality values obtained with the real data were compared to the null distributions to compute empirical *p*-values and log fold-change measures, comparing the true versus expected value in the randomized setting.

Quantifying Wnt3a effects on T cell IL-2 secretion

Wildtype CD8 T cells were treated with 200ng/mL of recombinant human Wnt3a protein (R&D Systems, 5036-WN-010/CF) for 72 hours. After 72 hours, the supernatant was collected, centrifuged at 400xg for 5 min, and stored at -80°C before the IL-2 ELISA assay. The concentration of IL-2 was quantified using the ELISA MAX

Deluxe Set Human IL-2 kit (Biolegend, 431804) according to the manufacturer's protocol. A standard curve was plotted on a log-log axis using GraphPad Prism's nonlinear regression (curve fit) analysis. The unknown analyte concentration was then calculated according to the manufacturer's protocol using the line of best fit.

Supplementary References

1. Norman, T. M. *et al.* Exploring genetic interaction manifolds constructed from rich single-cell phenotypes. *Science* **365**, 786–793 (2019).
2. Lawson, K. A. *et al.* Functional genomic landscape of cancer-intrinsic evasion of killing by T cells. *Nature* **586**, 120–126 (2020).
3. Patel, S. J. *et al.* Identification of essential genes for cancer immunotherapy. *Nature* **548**, 537–542 (2017).
4. Manguso, R. T. *et al.* In vivo CRISPR screening identifies Ptpn2 as a cancer immunotherapy target. *Nature* **547**, 413–418 (2017).
5. Griffin, G. K. *et al.* Epigenetic silencing by SETDB1 suppresses tumour intrinsic immunogenicity. *Nature* **595**, 309–314 (2021).
6. Joung, J. *et al.* CRISPR activation screen identifies BCL-2 proteins and B3GNT2 as drivers of cancer resistance to T cell-mediated cytotoxicity. *Nature Communications* **13**, 1606 (2022).
7. Sheffer, M. *et al.* Genome-scale screens identify factors regulating tumor cell responses to natural killer cells. *Nature Genetics* **53**, 1196–1206 (2021).
8. Dubrot, J. *et al.* In vivo CRISPR screens reveal the landscape of immune evasion pathways across cancer. *Nat Immunol* **23**, 1495–1506 (2022).
9. Han, K. *et al.* Synergistic drug combinations for cancer identified in a CRISPR screen for pairwise genetic interactions. *Nat Biotechnol* **35**, 463–474 (2017).
10. Levin-Konigsberg, R. *et al.* An SLC12A9-dependent ion transport mechanism maintains lysosomal osmolarity. *Dev Cell* **60**, 220-235.e7 (2025).
11. Genomic Classification of Cutaneous Melanoma. *Cell* **161**, 1681–1696 (2015).
12. Yeh, C. Y. *et al.* Mapping spatial organization and genetic cell-state regulators to target immune evasion in ovarian cancer. *Nature Immunology* https://doi.org/10.1038/s41590-024-01943-5 (2024) doi:10.1038/s41590-024-01943-5.
13. Replogle, J. M. *et al.* Mapping information-rich genotype-phenotype landscapes with genome-scale Perturb-seq. *Cell* **185**, 2559-2575.e28 (2022).
14. Peidli, S. *et al.* scPerturb: harmonized single-cell perturbation data. *Nat Methods* **21**, 531–540 (2024).
