# Supplementary material for: High-content CRISPR activation screens identify synthetically lethal RNA-based mechanisms to sensitize cancer cells to targeted T cell cytotoxicity
Source: Nat Genet. 2026 Apr 7;58(4):841–53. doi: 10.1038/s41588-026-02561-7 (PMC13083246; doi:10.1038/s41588-026-02561-7)
Supplement: Supplementary file 2 — Reporting Summary [file 41588_2026_2561_MOESM2_ESM.pdf]

nature portfolio

Corresponding author(s): Livnat Jerby

Last updated by author(s): Jan 22, 2026

Reporting Summary

Nature Portfolio wishes to improve the reproducibility of the work that we publish. This form provides structure for consistency and transparency in reporting. For further information on Nature Portfolio policies, see our Editorial Policies and the Editorial Policy Checklist.

Statistics

For all statistical analyses, confirm that the following items are present in the figure legend, table legend, main text, or Methods section.

n/a	Confirmed	
☐	☒	The exact sample size (*n*) for each experimental group/condition, given as a discrete number and unit of measurement
☐	☒	A statement on whether measurements were taken from distinct samples or whether the same sample was measured repeatedly
☐	☒	The statistical test(s) used AND whether they are one- or two-sided<br>*Only common tests should be described solely by name; describe more complex techniques in the Methods section.*
☐	☒	A description of all covariates tested
☐	☒	A description of any assumptions or corrections, such as tests of normality and adjustment for multiple comparisons
☐	☒	A full description of the statistical parameters including central tendency (e.g. means) or other basic estimates (e.g. regression coefficient) AND variation (e.g. standard deviation) or associated estimates of uncertainty (e.g. confidence intervals)
☐	☒	For null hypothesis testing, the test statistic (e.g. *F*, *t*, *r*) with confidence intervals, effect sizes, degrees of freedom and *P* value noted<br>*Give P values as exact values whenever suitable.*
☒	☐	For Bayesian analysis, information on the choice of priors and Markov chain Monte Carlo settings
☐	☒	For hierarchical and complex designs, identification of the appropriate level for tests and full reporting of outcomes
☐	☒	Estimates of effect sizes (e.g. Cohen's *d*, Pearson's *r*), indicating how they were calculated

Our web collection on statistics for biologists contains articles on many of the points above.

Software and code

Policy information about availability of computer code

Data collection

In situ Perturb-seq data collection was performed via Nanostring CosMx Spatial Molecular Imaging (SMI). Single cell RNA-Seq and Perturb-Seq data were collected via 10x Chromium Controller. Flow cytometry was performed on a Sony SH800, BD FACSAria Fusion, BD FACSAria II, or BD Influx flow cytometer.

Data analysis

The following software, packages, and open-source code were used to analyze the data for the following categories

Data visualization, graphing, and plotting
- FlowJo (version 10.10.0)
- FACSDiva software (version 8.0.1) BD BioScience
- ImageLab (version 6.1.0) Bio-Rad
- CFX Manager Software (version 3.1) Bio-Rad
- GraphPad PRISM GraphPad Software (version 10.2.3)
- ggplot2 (version 3.4.2)
- RColorBrewer (version 1.1-3)
- Adobe Illustrator software (version 2026)

Basic data/environment handling and statistical Analysis in R
- R (version 4.5.2)
- lmerTest (version 3.1-3)
- lme4 (version 1.1-35.4)
- RStudio (version 2023.12.1)

High Performance Computing
• Slurm Workload Manager SchedMD https://slurm.schedmd.com/

Analyses of CRISPR screens and CRISPR screens with scRNA-seq and spatial transcriptomics readouts
• MAGeCK (version 0.5.9.4; https://github.com/liulab-dfci/MAGeCK)
• 10x Genomics Cell Ranger (version 7.1.0)
• Seurat (version 5.3.1)
• SeuratObject (version 5.2.0)
• edgeR (version 3.40.2)
• MAST (version 1.36.0)
• AtoMx
• Mesmer (https://github.com/vanvalenlab/publication-figures/tree/master/2021-Greenwald_Miller_et_al-Mesmer)

Code to reproduce results and figures presented in this study will be made available as a GitHub repository in https://github.com/Jerby-Lab/High_content_CRISPR_immunomodulators DOI: 10.6084/m9.figshare.31119718

For manuscripts utilizing custom algorithms or software that are central to the research but not yet described in published literature, software must be made available to editors and reviewers. We strongly encourage code deposition in a community repository (e.g. GitHub). See the Nature Portfolio guidelines for submitting code & software for further information.

Data

Policy information about availability of data

All manuscripts must include a data availability statement. This statement should provide the following information, where applicable:

- Accession codes, unique identifiers, or web links for publicly available datasets
- A description of any restrictions on data availability
- For clinical datasets or third party data, please ensure that the statement adheres to our policy

The data collected in this study are provided via a figshare repository, including the CRISPRa screen (10.6084/m9.figshare.31128976), Perturb-seq (10.6084/m9.figshare.31119196) and in situ Perturb-seq (10.6084/m9.figshare.31119634, 10.6084/m9.figshare.31119607) data. Gene expression TCGA data was obtained from the International Cancer Genome Consortium (ICGC) repository (https://docs.icgc-argo.org/)33. Previously published CRISPRa Perturb-seq data were downloaded from the Gene Expression Omnibus (GEO; GSE133344)37.

Research involving human participants, their data, or biological material

Policy information about studies with human participants or human data. See also policy information about sex, gender (identity/presentation), and sexual orientation and race, ethnicity and racism.

Reporting on sex and gender	Primary CD8 T cells were derived from deidentified blood samples of both male and female donors.<br><br>All cell lines were obtained from vendors and authenticated. Below we provide additional information about origin of the cell lines used in this study.<br>- A375: 54-year-old female melanoma patient of European descent.<br>- CaSki: 40-year-old female epidermoid carcinoma patient of European descent.<br>- MCF7: 69-year-old female caucasian suffering from a breast adenocarcinoma.
Reporting on race, ethnicity, or other socially relevant groupings	A375 and CaSki cell lines were derived from patients of European descent.<br>MCF7 cell line was derived from Caucasian patient.
Population characteristics	N/A
Recruitment	N/A
Ethics oversight	Blood samples were not collected specifically for this study, but were obtained from the Stanford Blood Center. The Center has an approved IRB (Institutional Review Board) protocol that is managed by Stanford University.

Note that full information on the approval of the study protocol must also be provided in the manuscript.

Field-specific reporting

Please select the one below that is the best fit for your research. If you are not sure, read the appropriate sections before making your selection.

☒ Life sciences ☐ Behavioural & social sciences ☐ Ecological, evolutionary & environmental sciences

For a reference copy of the document with all sections, see nature.com/documents/nr-reporting-summary-flat.pdf

Life sciences study design

All studies must disclose on these points even when the disclosure is negative.

Sample size	Sample size of screens was determined based on the required coverage to ensure robust statistical testing. No statistical method was used to predetermine sample size. Experiments testing specific targets were performed in replicates and with different cell lines and T cells derived from different donors to test for statistical significance and generalizability.
Data exclusions	No data were excluded from the analyses other than in downstream Perturb-seq analyses that were performed only with cells that passes quality control criteria as described in the Methods section
Replication	Every in vitro experiment was performed with at least 3 replicates, and/or with multiple biological replicates. Results were validated with different methods (e.g., both ORF and CRISPR activation; scRNA-seq and spatial transcriptomics). In vivo experiments were performed such that tens of thousands of cells were sequenced per mouse, providing a highly controlled setting to determine the effects of each perturbation while eliminating inter-mouse variation.
Randomization	The pooled CRISPR and ORF screens that were performed were randomized by default. Readouts for other experiments were performed in an automated manner. There was no comparison across groups in any of the in vivo experiments conducted in this study.
Blinding	Investigators were not blinded to experimental group allocation in experiments validating or investigating specific hits or treatments. The bulk of the study was performed via multiplexed experiments and screens, which are inherently randomized, such that investigators are blinded to the allocation of cells to experimental groups.

Reporting for specific materials, systems and methods

We require information from authors about some types of materials, experimental systems and methods used in many studies. Here, indicate whether each material, system or method listed is relevant to your study. If you are not sure if a list item applies to your research, read the appropriate section before selecting a response.

Materials & experimental systems

n/a	Involved in the study	
☐	☒	Antibodies
☐	☒	Eukaryotic cell lines
☒	☐	Palaeontology and archaeology
☐	☒	Animals and other organisms
☒	☐	Clinical data
☒	☐	Dual use research of concern
☒	☐	Plants

Methods

n/a	Involved in the study	
☒	☐	ChIP-seq
☐	☒	Flow cytometry
☒	☐	MRI-based neuroimaging

Antibodies

Antibodies used	The following antibodies were used:<br>• Alpha tubulin, 1:1,000 dilution (Cell Signaling Technology, Cat#2144S, Lot 7)<br>• Anti-rabbit IgG, HRP-linked, 1:1,000 dilution (Cell Signaling Technology, Cat#7074S, Lot 30)<br>• Anti-mouse IgG HRP-linked, 1:1,000 dilution (Cell Signaling Technology, Cat#7076S, Lot 36)<br>• HPC4-tag, 1:50 dilution (Cell Signaling Technology, Cat#68083S, Lot 1)<br>• HA-tag (6E2) Mouse, 1:50 dilution (Cell Signaling Technology, Cat#2367S, Lot 5)<br>• Anti-rabbit IgG (H+L), F(ab')2 fragment (Alexa Fluor 647 conjugate), 1:500 dilution (Cell Signaling Technology, Cat#4414S, Lot 24)<br>• Anti-mouse IgG (H+L), F(ab')2 fragment (Alexa Fluor 488 conjugate), 1:500 dilution (Cell Signaling Technology, Cat#4408S, Lot 22)<br>• NY-ESO-1 (E978), 1:500 dilution (Santa Cruz Biotechnology, Cat#sc-53869, Lot H0621)<br>• CD2 Monoclonal Antibody (RPA-2.10), 1:20 dilution (eBioscience, Cat#17-0029-42, Lot 2219334)<br>• Caspase-3, 1:1,000 dilution (Cell Signaling Technology, Cat#9662S, Lot 19)<br>• HPV16 E7, (NM2), 1:50 dilution (Santa Cruz Biotechnology, Cat#sc-65711, Lot J1223)<br>• β-actin (13E5) Rabbit Monoclonal Antibody 1:5,000 dilution (Cell Signaling Technology Cat#4970S, Lot 18)<br>• DAPI, 1:10,000 dilution (Thermo Fisher Scientific Cat#62248)<br>• APC anti-human HLA-A2 Antibody, Clone BB7.2, 1:100 dilution (BioLegend, Cat#343307, Lot B356099)<br>• APC Mouse IgG2b, k Isotype Ctrl Antibody, Clone MPC-11, 1:100 dilution (BioLegend Cat#400321, Lot B319961)<br>• Alexa Fluor 488 anti-mouse TCR β chain Antibody, Clone H57-597, 1:500 dilution (BioLegend Cat#109215, LotB349876)<br>• Purified anti-c-Myc Antibody, Clone 9E10, 1:1,000 dilution (BioLegend Cat#626801, Lot B423723)<br>• Human TruStain FcX™ (Fc Receptor Blocking Solution), 1:50 dilution (BioLegend, Cat#422302, Lot B446604)<br>• Purified anti-mouse CD16/32 Antibody, Clone 93, 1:50 dilution (BioLegend, Cat#101302, Lot B423718)<br>• NY-ESO-1 Rabbit Monoclonal Antibody (D1Q2U), Alexa Fluor 647 Conjugate, Clone D1Q2U, 1:50 dilution (Cell Signaling Technology, Cat#66920S, Lot 1)
Validation	All antibodies were validated for the specific application by the manufacturer and validation data is available on the manufacturer's website.

- Alpha tubulin Antibody, 1:1,000 dilution, (Cell Signaling Technology, Cat#2144S, Lot 7) https://www.cellsignal.com/products/primary-antibodies/a-tubulin-antibody/2144
- Anti-rabbit IgG, HRP-linked Antibody, 1:1,000 dilution, (Cell Signaling Technology, Cat#7074S, Lot 30) https://www.cellsignal.com/products/secondary-antibodies/anti-rabbit-igg-hrp-linked-antibody/7074
- Anti-mouse IgG HRP-linked Antibody, 1:1,000 dilution, (Cell Signaling Technology, Cat#7076S, Lot 36) https://www.cellsignal.com/products/secondary-antibodies/anti-rabbit-igg-hrp-linked-antibody/7074
- HPC4-Tag Antibody, 1:50 dilution, (Cell Signaling Technology, Cat#68083S, Lot 1) https://www.cellsignal.com/products/primary-antibodies/hpc4-tag-antibody/68083
- HA-tag (6E2) Mouse mAb, 1:50 dilution, (Cell Signaling Technology, Cat#2367S, Lot 5) https://www.cellsignal.com/products/primary-antibodies/ha-tag-6e2-mouse-mab/2367
- Anti-rabbit IgG (H+L), F(ab')2 fragment (Alexa Fluor 647 conjugate), 1:500 dilution, (Cell Signaling Technology, Cat#4414S, Lot 24) https://www.cellsignal.com/products/secondary-antibodies/anti-rabbit-igg-h-l-f-ab-2-fragment-alexa-fluor-647-conjugate/4414
- Anti-mouse IgG (H+L), F(ab')2 fragment (Alexa Fluor 488 conjugate), 1:500 dilution, (Cell Signaling Technology, Cat#4408S, Lot 22) https://www.cellsignal.com/products/secondary-antibodies/anti-mouse-igg-h-l-f-ab-2-fragment-alexa-fluor-488-conjugate/4408
- NY-ESO-1 Antibody (E978), 1:500 dilution, (Santa Cruz Biotechnology, Cat#sc-53869, Lot H0621) https://www.scbt.com/p/ny-eso-1-antibody-e978
- CD2 Monoclonal Antibody (RPA-2.10), 1:20 dilution, (eBioscience, Cat#17-0029-42) https://www.thermofisher.com/antibody/product/CD2-Antibody-clone-RPA-2-10-Monoclonal/17-0029-42
- Caspase-3, 1:1,000 dilution, (Cell Signaling Technology, Cat#9662S) https://www.cellsignal.com/products/primary-antibodies/caspase-3-antibody/9662
- HPV16 E7 (NM2), 1:50 dilution, (Santa Cruz Biotechnology, Cat#sc-65711) https://www.scbt.com/p/hpv16-e7-antibody-nm2
- β-actin (13E5) Rabbit Monoclonal Antibody, 1:5,000 dilution, (Cell Signaling Technology Cat#4970S) https://www.cellsignal.com/products/primary-antibodies/b-actin-13e5-rabbit-mab/4970
- DAPI, 1:10,000 dilution, (Thermo Fisher Scientific Cat#62248) https://www.thermofisher.com/order/catalog/product/62248
- APC anti-human HLA-A2 Antibody, Clone BB7.2, 1:100 dilution (BioLegend, Cat#343307, Lot B356099) https://www.biolegend.com/en-us/products/apc-anti-human-hla-a2-antibody-8181
- APC Mouse IgG2b, k Isotype Ctrl Antibody, Clone MPC-11, 1:100 dilution (BioLegend Cat#400321, Lot B319961) https://www.biolegend.com/en-us/products/apc-mouse-igg2b-kappa-isotype-ctrl-1410
- Alexa Fluor 488 anti-mouse TCR β chain Antibody, Clone H57-597, 1:500 dilution (BioLegend Cat#109215, LotB349876) https://www.biolegend.com/en-us/products/alexa-fluor-488-anti-mouse-tcr-beta-chain-antibody-2713
- Purified anti-c-Myc Antibody, Clone 9E10, 1:1,000 dilution (BioLegend Cat#626801, Lot B423723) https://www.biolegend.com/en-us/products/purified-anti-c-myc-antibody-2873
- Human TruStain FcX™ (Fc Receptor Blocking Solution), 1:50 dilution (BioLegend, Cat#422302, Lot B446604) https://www.biolegend.com/en-us/products/human-trustain-fcx-fc-receptor-blocking-solution-6462
- Purified anti-mouse CD16/32 Antibody, Clone 93, 1:50 dilution (BioLegend, Cat#101302, Lot B423718) https://www.biolegend.com/en-us/products/purified-anti-mouse-cd16-32-antibody-190

Eukaryotic cell lines

Policy information about cell lines and Sex and Gender in Research

Cell line source(s)	The cell lines used in this study are: LentiX 293T (Takara Bio, 632180), MCF7 (Sigma, 86012803), A375 (ATCC, CRL-1619), CaSki (ATCC, CRL-1550), MC38 (Sigma-Aldrich, SCC172), and B16-OVA MO4 (Sigma-Aldrich, SCC420). In addition, primary CD8+ T cells were derived from PBMCs (Stanford Blood Center) and confirmed via flow cytometry and cytotoxicity co-culture assays to be functional CD8+ T cells.
Authentication	All cell lines were authenticated through ATCC using short tandem repeat profiling.
Mycoplasma contamination	All cells were regularly tested for mycoplasma (Boca Scientific, 25235) and confirmed negative before use in experiments.
Commonly misidentified lines (See ICLAC register)	None

Animals and other research organisms

Policy information about studies involving animals; ARRIVE guidelines recommended for reporting animal research, and Sex and Gender in Research

Laboratory animals	Immunocompromised mouse experiments were performed in NSG and hIL-2 NOG mice (Mus musculus). Male NOD.Cg-Prkdcscid Il2rgtm1Wjl/SzJ (n = 18) and female NOD.Cg-Prkdcscid Il2rgtm1Sug Tg(CMV-IL2)4-2Jic/JicTac (n = 4) mice were obtained from The Jackson Laboratory (Cat# 005557) or Taconic (Cat# 13440-F), respectively. Animals were 6-8 weeks of age at the time of tumor cell injection.<br><br>Immunocompetent mouse experiments were performed in C57BL/6 mice (Mus musculus). Male C57BL/6J (n = 7) mice were obtained from The Jackson Laboratory (Cat# 000664), and male and female C57BL/6NCrl (n = 10 and 6, respectively) mice were obtained from Charles River Laboratories (Cat# 027). Animals were 9-14 weeks of age at the time of tumor cell injection.<br><br>All mice were housed under controlled environmental conditions, in 12-h light/12-h dark cycles at room temperatures ranging between 20-26 °C and humidities between 30-70%. Mice were fed 2018 Teklad 18% protein rodent diet and water ad libitum.
Wild animals	None
Reporting on sex	The sex of the mice used in each experiment are reported in the Methods section for each of the in vivo experiments conducted.

Field-collected samples: None

Ethics oversight: Mice were housed at Stanford University, an AAALAC accredited animal care and use program. For all animal experiments, protocols were approved by Stanford's Administrative Panel on Laboratory Animal Care (APLAC-34218).

Note that full information on the approval of the study protocol must also be provided in the manuscript.

Plants

Seed stocks: N/A

Novel plant genotypes: N/A

Authentication: N/A

Flow Cytometry

Plots

Confirm that:

☒ The axis labels state the marker and fluorochrome used (e.g. CD4-FITC).

☒ The axis scales are clearly visible. Include numbers along axes only for bottom left plot of group (a 'group' is an analysis of identical markers).

☒ All plots are contour plots with outliers or pseudocolor plots.

☒ A numerical value for number of cells or percentage (with statistics) is provided.

Methodology

Sample preparation: For cells from in vitro experiments, adherent cells were detached with trypsin, quenched with media containing FBS, then washed twice with PBS. Cells were stained with antibodies following the protocols outlined in the methods section, then filtered with a 0.45 uM strainer and resuspended in PBS + 5% FBS for sorting.

Instrument: For analysis and sorting, experiments were performed on a Sony Biotechnology SH800, BD FACSAria Fusion, BD FACSAria II, or BD Influx flow cytometer.

Software: FlowJo version 10.10.

Cell population abundance: Cell purity was analyzed when generating the monoclonal A375 cell line containing dCas9-VPR for use with the CRISPRa screens and CRISPRa-related experiments. The purity was determined to be 99.99%.

Gating strategy: For all experiments, cells were first gated by FSC-A vs SSC-A, then FSC-A vs FSC-W or FSC-A vs FSC-H then SSC-A vs SSC-H to obtain single cells. Gates for all experiments were determined by using an appropriate negative control. Supplementary Fig. 11 shows the gating strategy in detail.

☒ Tick this box to confirm that a figure exemplifying the gating strategy is provided in the Supplementary Information.
