# Supplementary figures and images for: High-content CRISPR activation screens identify synthetically lethal RNA-based mechanisms to sensitize cancer cells to targeted T cell cytotoxicity
Source: Nat Genet. 2026 Apr 7;58(4):841–53. doi: 10.1038/s41588-026-02561-7 (PMC13083246; doi:10.1038/s41588-026-02561-7)

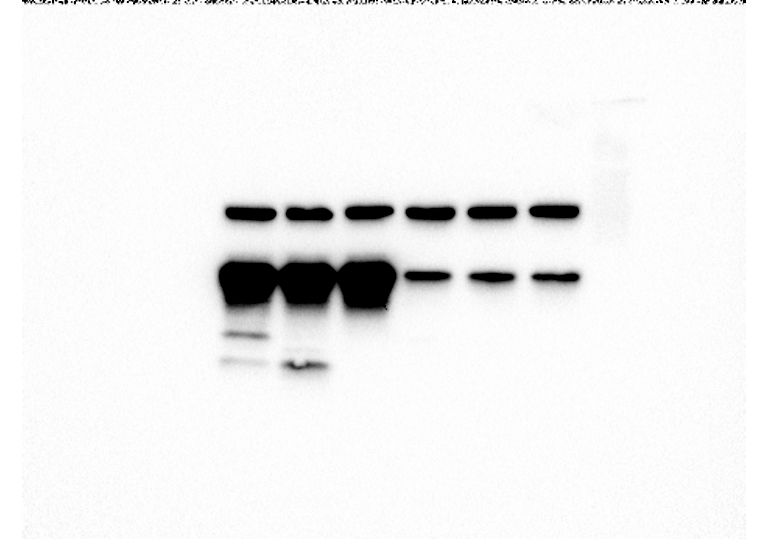

Supplement: Supplementary file 4 — Unprocessed western blot. [file 41588_2026_2561_MOESM4_ESM.jpg]

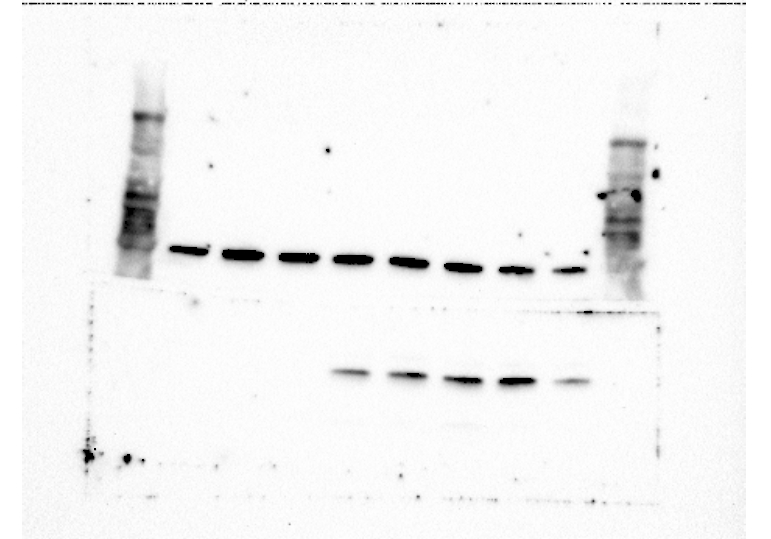

Supplement: Supplementary file 6 — Unprocessed western blot. [file 41588_2026_2561_MOESM6_ESM.jpg]

$\beta$ -Actin

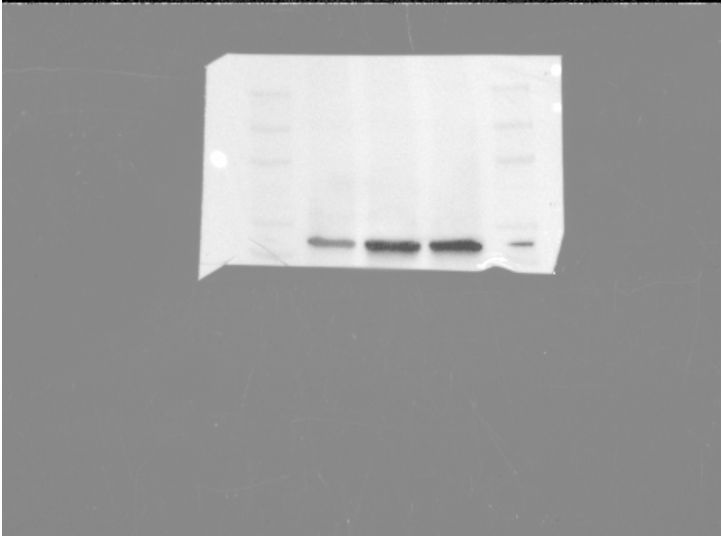

HPV16 E7

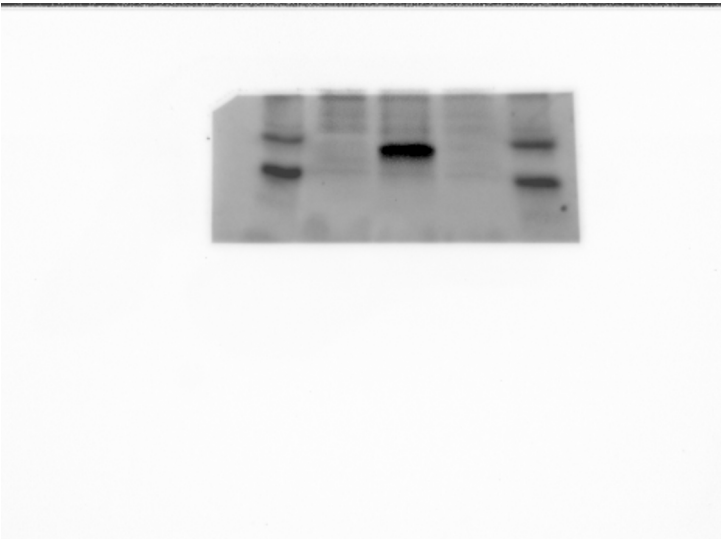

Supplement: Supplementary file 7 — Unprocessed western blot. [file 41588_2026_2561_MOESM7_ESM.pdf]

MYC

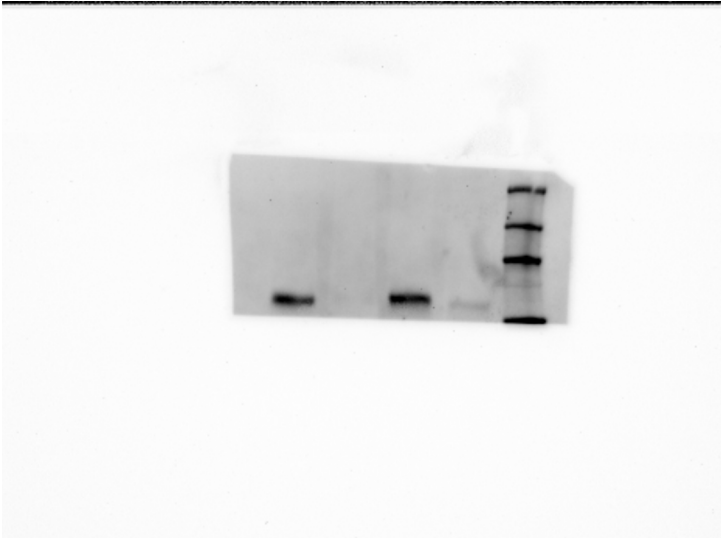

$\beta$ -Actin

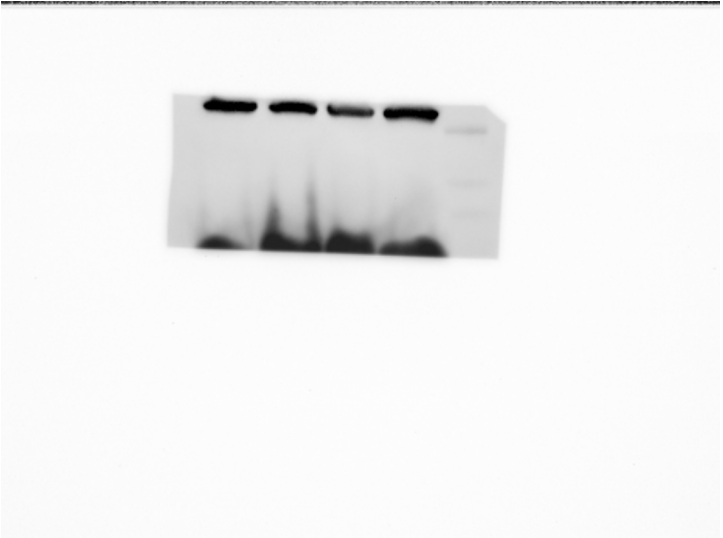

Supplement: Supplementary file 8 — Unprocessed western blot. [file 41588_2026_2561_MOESM8_ESM.pdf]
